# Global, regional, and national burden of pneumococcal disease among children and adolescents aged <20 years from 1990 to 2021: a predictive analysis

**DOI:** 10.3389/fpubh.2025.1675854

**Published:** 2025-12-05

**Authors:** Gan-min Wang, Wei Tao, Xiang-yang Pang, Yi Xin, Zi-han Gou, Yao Wang

**Affiliations:** 1 Department of Anesthesiology, The Thirteenth People’s Hospital of Chongqing, Chongqing, China; 2 Department of Anesthesiology, The First Affiliated Hospital of Chongqing Medical University, Chongqing, China; 3 Department of Anesthesiology, Stomatological Hospital Affiliated of Chongqing Medical University, Chongqing, China; 4 Department of Anesthesiology, The People's Hospital of Kaizhou District Chongqing, Chongqing, China

**Keywords:** pneumococcal disease, children and adolescents, GBD 2021, ASMR, ASDR, health inequality, prediction

## Abstract

**Background:**

Pneumococcal infections remain a leading cause of preventable deaths among children and adolescents aged <20 years. Despite a global decrease in burden, significant regional and socioeconomic disparities, as well as long-term trends, are not fully understood.

**Methods:**

We used data from the Global Burden of Disease Study 2021 (GBD 2021) to evaluate mortality and disability-adjusted life years (DALYs) attributable to pneumococcal infections among those aged 0–19 years across global, regional, and 204 national settings from 1990 to 2021. Temporal trends were assessed using estimated annual percentage change (EAPC), and Spearman correlation examined associations with the sociodemographic index (SDI). Decomposition analysis quantified the contributions of demographic and epidemiological drivers. Health inequality was assessed via the slope index of inequality (SII) and concentration index (CI). A Bayesian age-period-cohort (BAPC) model projected future burden to 2036.

**Results:**

In 2021, pneumococcal infections caused 179,354 deaths (95% *UI*: 142,347–217,280) and 15,757,828 DALYs (95% *UI*: 12,500,395–19,088,138) among individuals aged 0–19 years, with the highest burden in children under 5. From 1990 to 2021, global age-standardized mortality rate (ASMR) dropped from 36.18 to 6.80 per 100,000 (EAPC –4.89, 95% *CI*: −5.23 to −4.54). High-middle SDI regions had the largest decline (EAPC –8.63%), while Oceania had the smallest (EAPC –2.20%). Epidemiological changes were the main drivers of burden reduction, partly offset by population growth. The SII for ASMR and age-standardized DALY rate (ASDR) decreased from −83.91 to −11.19 and from −7,395.42 to −975.38, respectively, indicating a reduction in absolute inequality, while the CI for both increased from 0.47 to 0.55, suggesting a growing relative concentration of burden in high-SDI countries despite the persistently high absolute burden in low-SDI regions. BAPC projections indicate the global ASMR will fall to 1.59 per 100,000 (95% *UI*: 0.82–2.35) by 2036.

**Conclusion:**

Over the past 30 years, the global burden of pneumococcal disease among children and adolescents has significantly declined. However, high burdens persist in low-SDI regions and among children under 5, with increasing relative inequalities. Strengthening vaccination coverage, healthcare systems, and interventions for high-risk populations is essential to further reduce the global burden.

## 1 Introduction

*Streptococcus pneumoniae* is one of the leading pathogens responsible for preventable deaths and disease burdens in children and adolescents worldwide. It causes severe invasive diseases such as pneumonia, meningitis, and sepsis (1, 2). Among children and adolescents aged <20 years, the risk of severe illness and death following pneumococcal infection is significantly greater than in adults due to their immature immune system. Furthermore, susceptibility and disease spectra vary across different age groups associated with pneumococcal infection (3, 4). Global data show that despite significant progress in vaccination and comprehensive health interventions over the past two decades, pneumococcal infections still cause hundreds of thousands of deaths in children and adolescents annually (5). Additionally, pneumococcal infections have long-term impacts on the health, education, and future productivity of children and adolescents, posing a major public health challenge (6).

Although some studies have assessed the pneumococcal burden in different countries and regions, long-term trend analyses in the under 20 population remain scarce (7). This group is often not prioritized in disease control strategies, with a lack of continuous monitoring and targeted catch-up vaccination programs, as the focus of vaccination has mainly been on infants (8). The rising issue of antibiotic resistance, serotype replacement, vaccination gaps, and the ongoing impact of conflicts and epidemics on health systems contribute to the uncertainty in future disease burden trends (9). In the context of global health equity and sustainable development goals, understanding the dynamic evolution, health inequalities, and potential improvements in pneumococcal disease burden over the past 30 years among the under 20 population is essential for developing evidence-based prevention strategies, optimizing resource allocation, and promoting health equity.

This study uses data from the Global Burden of Disease Study 2021 (GBD 2021) database to systematically quantify global, regional, and national trends in pneumococcal-related mortality and disability-adjusted life years (DALYs) from 1990 to 2021 for children and adolescents. We analyze the relationship with the sociodemographic index (SDI), assess the contributions of population growth, aging, and epidemiological changes, explore inequalities in burden, identify high-burden countries with potential for improvement, and use the Bayesian age-period-cohort (BAPC) model to predict trends through 2036. Through this research, we aim to provide the latest evidence for global pneumococcal prevention and control in children and adolescents, advancing the achievement of health equity goals.

## 2 Materials and methods

### 2.1 Data sources

This study is an observational, secondary data-based analysis of global trends in disease burden, covering the period from January 1, 1990, to December 31, 2021. This study aimed to assess the mortality and DALYs attributable to *Streptococcus pneumoniae* infections among children and adolescents aged 0–19 years at the global, regional, and national levels across 204 countries and territories. All data were obtained from the publicly available GBD 2021 database^1^ (10). GBD 2021 employed a systematic approach to address data sparsity and heterogeneity, primarily utilizing the Bayesian regularized trimmed meta-regression (MR-BRT) model to adjust for uncertainty and to trim outliers. The DisMod-MR 2.1 model was used to ensure internal consistency among epidemiological parameters, thereby improving data quality and the reliability of estimates while minimizing potential bias from data anomalies. In addition, GBD studies are supported by a global network of collaborators who contribute data, review inputs, and participate in the analytical process, thereby increasing the accuracy and robustness of the results (11).

### 2.2 Indicator definitions

The study indicators include the number of deaths, DALYs, ASMR, and ASDR (12). DALYs are the sum of years of life lost (YLLs) and years lived with disability (YLDs). Both the ASMR and ASDR were directly age-standardized on the basis of the GBD global standard population, reported per 100,000 people. The SDI is a composite measure of a country’s income, education, and fertility level, ranging from 0 to 1, and is categorized into five levels as low, low-middle, middle, high-middle, and high (13).

### 2.3 Statistical analysis

#### 2.3.1 Trend estimation

To quantify the changes in disease burden from 1990 to 2021, the estimated annual percentage change (EAPC) was used (14). First, the natural logarithms of the ASMR and ASDR for each year were taken, and linear regression models (ln(rate) = *α* + *β* × year) were applied. The EAPC was then calculated as EAPC = (*e*^*β* − 1) × 100%, with 95% confidence intervals (CIs) reported. A significant decline was defined when both the EAPC and its 95% CI were less than 0 (15).

#### 2.3.2 SDI correlation analysis

We extracted the ASMRs and ASDRs for 204 countries/regions in 1990 and 2021 and paired them with the SDI values for the same years. Spearman’s rank correlation coefficient (*ρ*) was used to analyze the monotonic correlation between the SDI and disease burden, with a significance level set at two-sided *p* < 0.05 (16). The analysis was performed via R software (v4.5.1, R Foundation for Statistical Computing) with the “stats” package.

#### 2.3.3 Decomposition analysis

According to the GBD decomposition framework, changes in deaths and DALYs were attributed to three factors, namely population size changes, population age structure changes and epidemiological changes. The contribution of each factor was expressed as a percentage. A Shapley value-based method was used to distribute the bidirectional and three-way interaction terms to ensure the robustness of the estimates (17). The analysis was performed via the R package deSolve (v1.35).

#### 2.3.4 Health inequality analysis

Health inequality was measured via the slope index of inequality (SII) and concentration index (CI) (18). The SII was calculated through a weighted least squares regression of the ASMR and ASDR against the SDI ranks to estimate the burden gap between the highest and lowest SDI groups. The CI was based on the Lorenz curve with values ranging from −1 to 1, where positive values indicate a higher concentration of burden in high-SDI countries (19). The analysis was conducted via the R package ineq (v0.2–13, functions Concentration. Index and SII).

#### 2.3.5 Frontier analysis

We used data envelopment analysis (DEA) and free disposal hull (FDH) models to estimate the efficiency frontier, with the SDI as the input variable and the ASDR as the output variable (20). DEA assumes variable returns to scale, with the direction of input minimization. FDH was used for robustness verification. The effective gap was defined as the difference between the observed values and the minimum frontier value, reflecting the potential for improvement (21). The analysis was performed via the R package Benchmarking (v0.30).

#### 2.3.6 BAPC predictive analysis

For future trend prediction, the BAPC model was used (22). The model used ASMR and ASDR data for 1990–2021, stratified by age (0–4, 5–9, 10–14, 15–19 years) and sex, with a second-order random walk (RW2) prior to smooth long-term trends. Posterior distributions were estimated via the integrated nested Laplace approximation (INLA). The median and 95% uncertainty intervals of the predicted output were provided (23). The analysis was performed via the R software BAPC package (v1.0.0) and INLA (v23.06.09).

All data processing and statistical analysis were performed via R (v4.5.1, R Foundation). Trend analysis was conducted via joinpoint, decomposition and inequality analysis via R packages, and maps and interactive results were created via Tableau Desktop (v2023.2).

## 3 Results

### 3.1 Global pneumococcal burden in children and adolescents in 2021

In 2021, pneumococcal infections led to 179,354 deaths (95% *UI*: 142,347–217,280) among children and adolescents aged <20 years globally (children defined as 0–9 years, adolescents as 10–19 years). Of these, 94,933 deaths (95% *UI*: 74,125–116,417) were male, and 84,421 deaths (95% *UI*: 67,891–100,800) were female. During the same period, the total number of DALYs due to pneumococcal infections was 15,757,828 (95% *UI*: 12,500,395–19,088,138), with 8,346,905 DALYs (95% *UI*: 6,508,339–10,228,242) for males and 7,410,922 DALYs (95% *UI*: 5,947,266–8,850,289) for females (Supplementary Figure S1; Supplementary Table S1).

**Figure 1:**
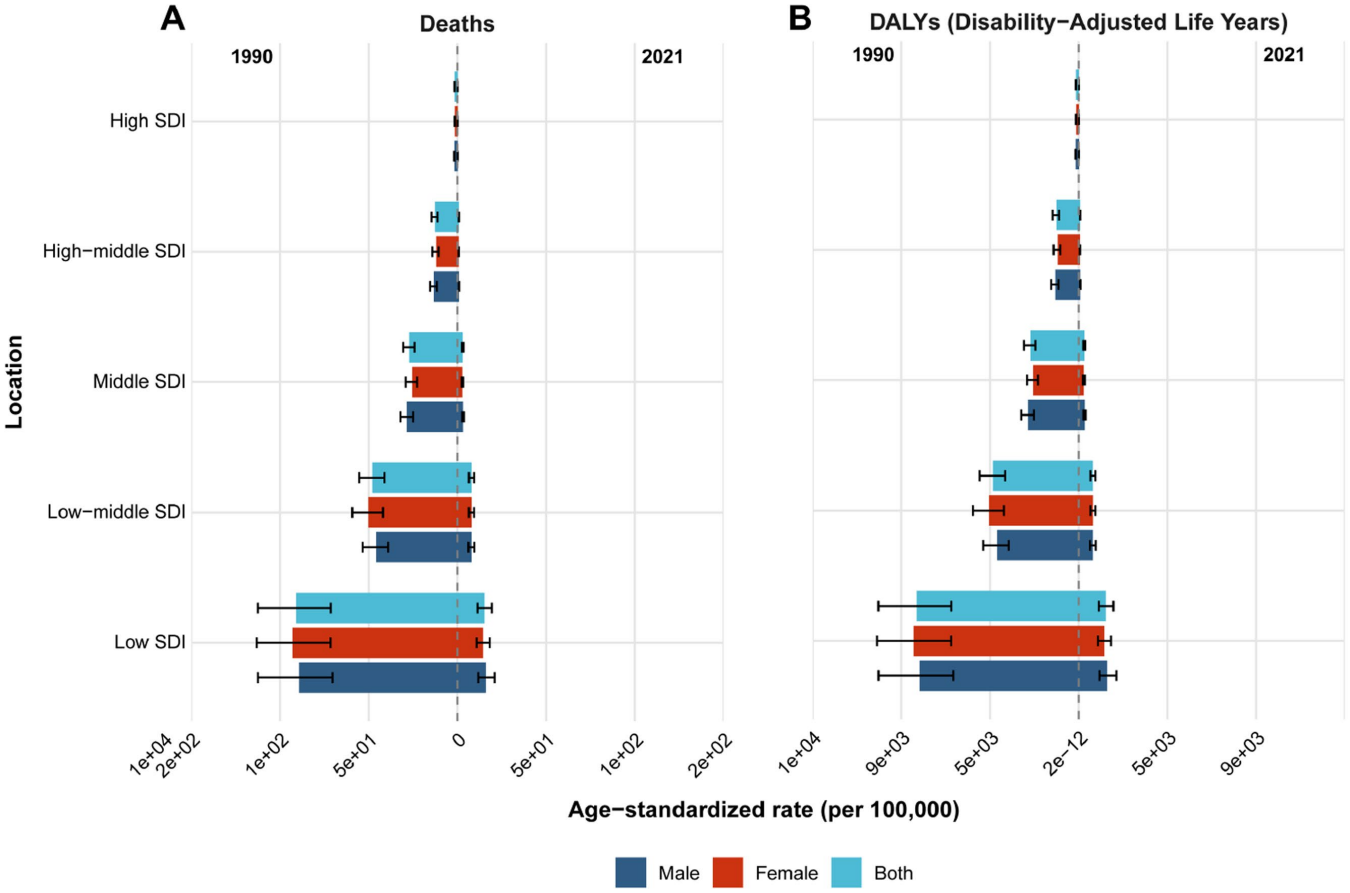
Age-standardized mortality rate (ASMR) **(A)** and age-standardized DALY rate (ASDR) **(B)** of pneumococcal disease among individuals aged <20 years in 1990 and 2021 across sociodemographic index (SDI) regions.

**Figure 2:**
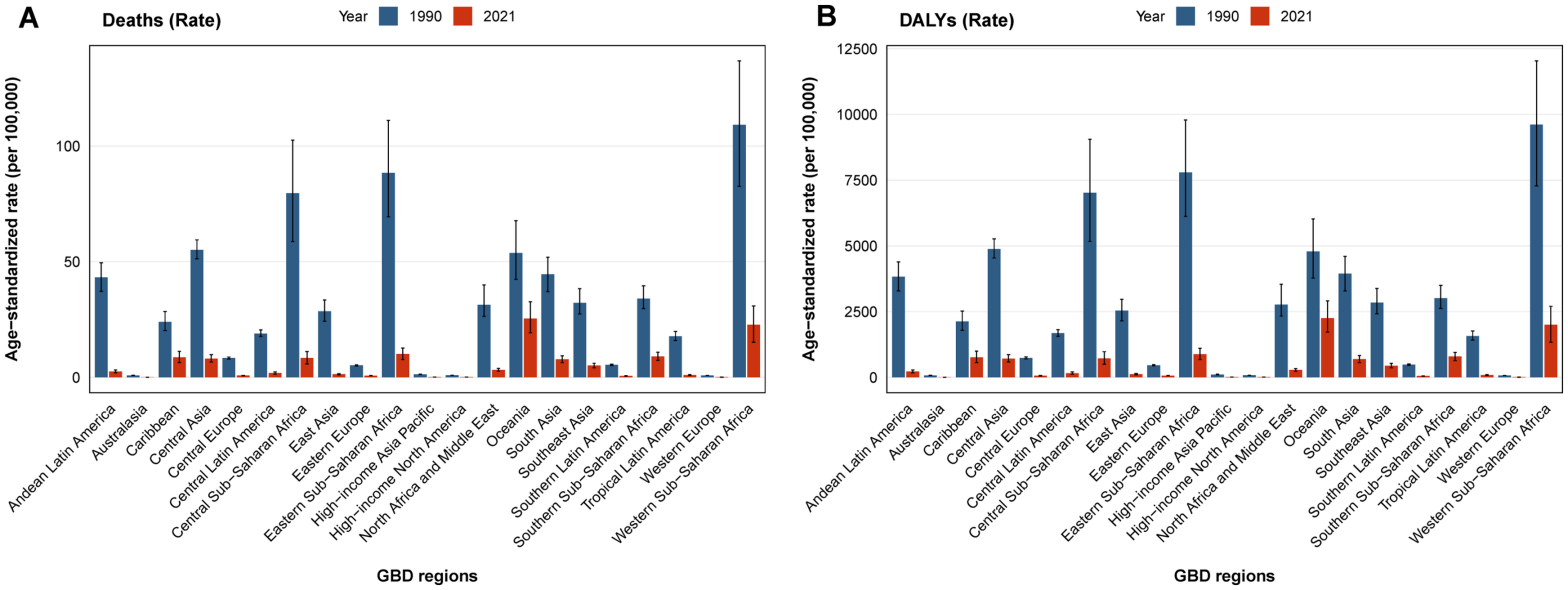
ASMR **(A)** and ASDR **(B)** of pneumococcal disease among individuals aged <20 years in 1990 and 2021 across 21 GBD regions.

In 2021, the global age-standardized mortality rate (ASMR) for children and adolescents was estimated at 6.80 per 100,000 (95% *UI*: 5.40–8.24), and the age-standardized DALY rate (ASDR) was 597.83 per 100,000 (95% *UI*: 474.25–724.18) (Table 1). Regionally, the highest ASMRs were observed in low-SDI regions (15.65 per 100,000, 95% *UI*: 11.57–20.00) and Oceania (25.37 per 100,000, 95% *UI*: 19.32–32.67). The highest ASDRs were also found in low-SDI regions (1,373.17 per 100,000, 95% *UI*: 1,014.99–1,754.14) and Oceania (2,255.63 per 100,000, 95% *UI*: 1,718.20–2,905.44) (Figures 1, 2; Table 1).

**Table 1:** Global and regional estimates of deaths and DALYs from pneumococcal disease among individuals aged <20 years in 1990 and 2021, including counts, ASRs per 100,000 population, and EAPCs.

Location	1990		2021		EAPC_95%*CI*
	Numbers (95% *UI*)	ASR per 100,000 (95% *UI*)	Numbers (95% *UI*)	ASR per 100,000 (95% *UI*)	
Deaths					
Global	817,218 (702,428–942,591)	36.18 (31.10–41.73)	179,354 (142,347–217,280)	6.80 (5.40–8.24)	−4.89 (−5.23 to −4.54)
High SDI	4,102 (3,791–4,519)	1.63 (1.51–1.80)	364 (333–393)	0.16 (0.14–0.17)	−7.47 (−7.57 to −7.37)
High-middle SDI	47,812 (42,359–55,328)	12.92 (11.44–14.95)	2,573 (2,219–2,987)	0.85 (0.73–0.98)	−8.63 (−8.77 to −8.48)
Middle SDI	212,667 (190,531–239,603)	27.82 (24.92–31.34)	23,266 (19,920–27,156)	3.11 (2.66–3.62)	−6.43 (−6.66 to −6.20)
Low-middle SDI	290,806 (250,230–336,389)	49.20 (42.34–56.92)	61,546 (50,633–72,749)	8.05 (6.62–9.52)	−5.22 (−5.60 to −4.84)
Low SDI	261,333 (205,203–322,769)	93.47 (73.40–115.45)	91,442 (67,603–116,816)	15.65 (11.57–20.00)	−5.50 (−5.89 to −5.10)
Andean Latin America	8,192 (7,051–9,395)	43.22 (37.19–49.56)	599 (458–757)	2.53 (1.93–3.20)	−8.70 (−9.08 to −8.32)
Australasia	51 (47–54)	0.81 (0.75–0.86)	5 (4–6)	0.07 (0.06–0.07)	−7.75 (−8.05 to −7.44)
Caribbean	3,625 (3,053–4,292)	24.01 (20.22–28.43)	1,317 (965–1,720)	8.63 (6.32–11.27)	−2.99 (−3.18 to −2.80)
Central Asia	17,391 (16,174–18,770)	55.07 (51.22–59.44)	2,789 (2,294–3,381)	8.06 (6.62–9.77)	−5.86 (−6.30 to −5.42)
Central Europe	3,272 (3,100–3,453)	8.33 (7.90–8.79)	179 (157–199)	0.76 (0.67–0.85)	−7.05 (−7.28 to −6.81)
Central Latin America	15,646 (14,557–16,940)	18.93 (17.62–20.50)	1,540 (1,207–1,979)	1.81 (1.42–2.32)	−7.37 (−7.56 to −7.18)
Central Sub-Saharan Africa	24,652 (18,188–31,796)	79.55 (58.69–102.61)	6,118 (4,228–8,214)	8.32 (5.75–11.17)	−6.90 (−7.60 to −6.20)
East Asia	131,614 (111,457–153,962)	28.60 (24.22–33.46)	4,557 (3,665–5,562)	1.32 (1.06–1.61)	−10.33 (−10.60 to −10.06)
Eastern Europe	3,474 (3,319–3,632)	5.16 (4.93–5.40)	333 (308–356)	0.72 (0.67–0.77)	−5.35 (−6.00 to −4.70)
Eastern Sub-Saharan Africa	98,043 (76,972–123,159)	88.41 (69.41–111.06)	23,010 (17,603–28,931)	10.11 (7.73–12.71)	−6.94 (−7.37 to −6.51)
High-income Asia Pacific	631 (577–701)	1.25 (1.15–1.39)	35 (33–38)	0.12 (0.11–0.12)	−7.33 (−7.84 to −6.81)
High-income North America	719 (694–745)	0.88 (0.85–0.91)	101 (93–109)	0.11 (0.10–0.12)	−6.88 (−7.22 to −6.54)
North Africa and Middle East	55,330 (46,638–70,630)	31.30 (26.38–39.96)	7,502 (6,294–9,035)	3.17 (2.66–3.82)	−6.42 (−6.83 to −6.01)
Oceania	1,810 (1,426–2,279)	53.77 (42.35–67.71)	1,621 (1,234–2,086)	25.37 (19.32–32.67)	−2.20 (−2.65 to −1.74)
South Asia	241,482 (201,284–281,589)	44.52 (37.11–51.91)	53,240 (43,353–63,904)	7.79 (6.34–9.35)	−4.94 (−5.30 to −4.57)
Southeast Asia	70,780 (60,303–84,334)	32.19 (27.42–38.35)	11,642 (9,565–14,022)	5.08 (4.17–6.12)	−5.62 (−5.89 to −5.35)
Southern Latin America	1,049 (997–1,108)	5.41 (5.15–5.72)	115 (100–130)	0.59 (0.51–0.67)	−6.55 (−6.94 to −6.15)
Southern Sub-Saharan Africa	9,005 (7,859–10,473)	34.03 (29.70–39.58)	2,837 (2,282–3,382)	9.07 (7.30–10.82)	−3.91 (−4.59 to −3.22)
Tropical Latin America	12,264 (11,052–13,763)	17.71 (15.96–19.87)	652 (531–789)	0.98 (0.80–1.19)	−8.95 (−9.36 to −8.53)
Western Europe	790 (768–810)	0.80 (0.78–0.82)	74 (68–79)	0.08 (0.07–0.09)	−7.45 (−7.71 to −7.20)
Western Sub-Saharan Africa	117,397 (88,771–147,085)	109.21 (82.58–136.83)	61,088 (40,705–82,776)	22.75 (15.16–30.82)	−4.60 (−5.08 to −4.13)
DALYs					
Global	72,235,581 (62,151,695–83,264,012)	3,198.28 (2,751.81–3,686.57)	15,757,828 (12,500,395–19,088,138)	597.83 (474.25–724.18)	−4.90 (−5.25 to −4.55)
High SDI	357,831 (330,576–394,769)	142.38 (131.54–157.08)	31,287 (28,668–33,738)	13.44 (12.32–14.50)	−7.51 (−7.61 to −7.41)
High-middle SDI	4,232,248 (3,747,771–4,899,073)	1,143.34 (1,012.46–1,323.48)	225,572 (194,185–261,675)	74.36 (64.01–86.26)	−8.66 (−8.80 to −8.51)
Middle SDI	18,820,176 (16,867,571–21,216,807)	2,461.59 (2,206.20–2,775.06)	2,040,491 (1,742,951–2,379,640)	272.36 (232.64–317.63)	−6.45 (−6.69 to −6.22)
Low-middle SDI	25,737,708 (22,156,497–29,781,377)	4,354.77 (3,748.83–5,038.95)	5,423,882 (4,461,171–6,421,352)	709.57 (583.63–840.06)	−5.23 (−5.61 to −4.84)
Low SDI	23,043,555 (18,112,515–28,469,337)	8,242.31 (6,478.56–10,183.03)	8,022,111 (5,929,659–10,247,792)	1,373.17 (1,014.99–1,754.14)	−5.51 (−5.91 to −5.11)
Andean Latin America	724,765 (623,312–831,671)	3,823.39 (3,288.19–4,387.35)	52,026 (39,747–65,851)	219.76 (167.90–278.16)	−8.76 (−9.14 to −8.37)
Australasia	4,455 (4,151–4,769)	71.01 (66.17–76.03)	434 (375–498)	5.75 (4.97–6.61)	−7.72 (−8.03 to −7.42)
Caribbean	321,097 (270,269–380,114)	2,126.61 (1,789.98–2,517.48)	116,478 (85,173–152,322)	763.16 (558.05–998.01)	−3.00 (−3.18 to −2.81)
Central Asia	1,541,155 (1,433,107–1,663,820)	4,880.18 (4,538.04–5,268.61)	245,453 (201,283–297,970)	708.90 (581.33–860.57)	−5.88 (−6.32 to −5.44)
Central Europe	289,238 (274,056–305,384)	736.57 (697.91–777.68)	15,307 (13,451–17,139)	64.98 (57.10–72.76)	−7.13 (−7.37 to −6.90)
Central Latin America	1,383,518 (1,286,756–1,498,124)	1,674.33 (1,557.23–1,813.02)	134,303 (105,169–172,927)	157.47 (123.31–202.76)	−7.42 (−7.61 to −7.22)
Central Sub-Saharan Africa	2,174,570 (1,602,120–2,807,221)	7,017.41 (5,170.10–9,059.00)	530,855 (365,449–717,421)	721.65 (496.80–975.28)	−6.95 (−7.66 to −6.23)
East Asia	11,658,588 (9,878,722–13,647,391)	2,533.71 (2,146.90–2,965.92)	402,393 (323,236–490,801)	116.65 (93.70–142.28)	−10.34 (−10.61 to −10.06)
Eastern Europe	308,149 (294,034–322,382)	458.04 (437.06–479.20)	28,762 (26,637–30,736)	62.31 (57.71–66.59)	−5.41 (−6.06 to −4.75)
Eastern Sub-Saharan Africa	8,642,230 (6,790,298–10,859,942)	7,793.00 (6,123.05–9,792.78)	2,006,842 (1,531,855–2,524,468)	881.80 (673.09–1,109.24)	−6.97 (−7.41 to −6.54)
High-income Asia Pacific	53,929 (49,198–59,885)	107.16 (97.76–118.99)	3,060 (2,868–3,273)	9.94 (9.31–10.63)	−7.29 (−7.80 to −6.78)
High-income North America	62,832 (60,600–65,147)	76.88 (74.15–79.71)	8,643 (7,934–9,366)	9.65 (8.86–10.46)	−6.94 (−7.28 to −6.59)
North Africa and Middle East	4,894,200 (4,121,212–6,256,424)	2,768.65 (2,331.37–3,539.26)	656,141 (549,705–788,618)	277.45 (232.44–333.47)	−6.45 (−6.87 to −6.04)
Oceania	161,063 (127,031–202,732)	4,784.44 (3,773.50–6,022.25)	144,053 (109,731–185,553)	2,255.63 (1,718.20–2,905.44)	−2.20 (−2.66 to −1.74)
South Asia	21,393,482 (17,829,288–24,944,698)	3,944.02 (3,286.94–4,598.71)	4,713,494 (3,835,655–5,660,246)	689.62 (561.18–828.13)	−4.93 (−5.29 to −4.57)
Southeast Asia	6,244,898 (5,319,507–7,439,628)	2,839.87 (2,419.04–3,383.17)	1,020,527 (837,998–1,230,315)	445.12 (365.50–536.62)	−5.64 (−5.92 to −5.36)
Southern Latin America	92,977 (88,352–98,280)	479.78 (455.91–507.14)	9,646 (8,403–11,011)	49.44 (43.07–56.44)	−6.71 (−7.11 to −6.30)
Southern Sub-Saharan Africa	795,930 (694,356–926,082)	3,007.97 (2,624.11–3,499.84)	247,459 (199,112–295,663)	791.50 (636.86–945.68)	−3.95 (−4.63 to −3.26)
Tropical Latin America	1,087,915 (980,193–1,220,760)	1,570.73 (1,415.20–1,762.53)	56,359 (45,953–68,390)	84.64 (69.01–102.71)	−9.04 (−9.46 to −8.61)
Western Europe	68,322 (66,423–70,039)	69.47 (67.54–71.22)	6,344 (5,859–6,830)	6.92 (6.39–7.45)	−7.45 (−7.70 to −7.20)
Western Sub-Saharan Africa	10,332,269 (7,826,217–12,938,720)	9,611.93 (7,280.59–12,036.66)	5,359,248 (3,584,258–7,257,871)	1,995.43 (1,334.54–2,702.35)	−4.61 (−5.09 to −4.13)

The EAPC was calculated using 31 years of data from 1990 to 2021. The table only includes data for 1990 and 2021 for comparative analysis. UI, uncertainty interval; ASR, age-standardized rate per 100,000; EAPC, estimated annual percentage change of ASR; CI, confidence interval.

**Figure 3:**
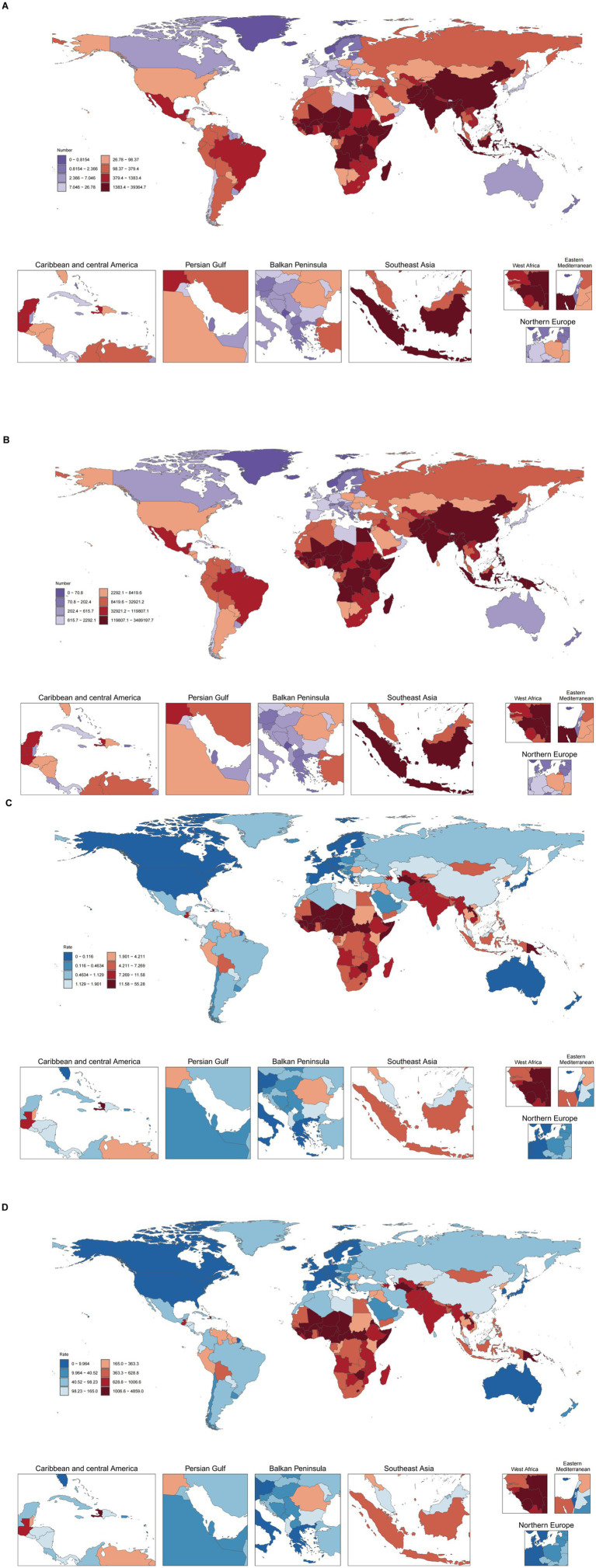
Numbers of deaths **(A)**, disability-adjusted life years (DALYs) **(B)**, ASMRs **(C)**, and ASDRs **(D)** from pneumococcal disease among individuals aged <20 years in 2021 across 204 countries.

At the national level, India had the largest burden, with 39,365 deaths (95% *UI*: 30,988–48,955) and 3,489,198 DALYs (95% *UI*: 2,744,611–4,342,836) (Figure 3; Supplementary Table S2). Spearman correlation analysis revealed a significant negative correlation between the ASMR (*ρ* = −0.8728, *p* < 0.001) and the ASDR (*ρ* = −0.8724, p < 0.001) and the SDI across the 204 countries (Supplementary Figure S2). Chad had the highest burden, with an ASMR of 55.28 per 100,000 (95% *UI*: 40.29–70.30) and an ASDR of 4,858.98 per 100,000 (95% *UI*: 3,543.27–6,184.59) (Figure 3; Supplementary Table S2).

**Figure 4:**
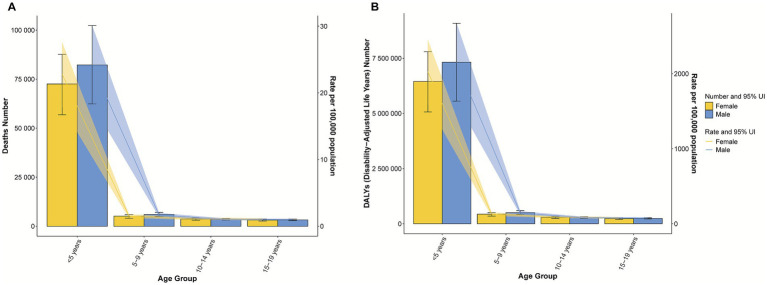
Numbers of deaths and ASMRs **(A)** and DALYs and ASDRs **(B)** of pneumococcal disease across different age groups (<20 years) in 2021.

Among the different age groups, children aged <5 years had the highest mortality rates and DALYs burdens. The ASMR and ASDR for this group peaked at 23.52 per 100,000 (95% *UI*: 18.23–28.83) and 2,092.13 per 100,000 (95% *UI*: 1,624.93–2,562.94), respectively (Supplementary Table S3). Additionally, the ASMR and ASDR for males were consistently greater than those for females (Figure 4; Supplementary Table S1). These results provide a foundation for subsequent trend and inequality analyses.

### 3.2 Trends in the global pneumococcal burden among children and adolescents, 1990–2021

**Figure 5:**
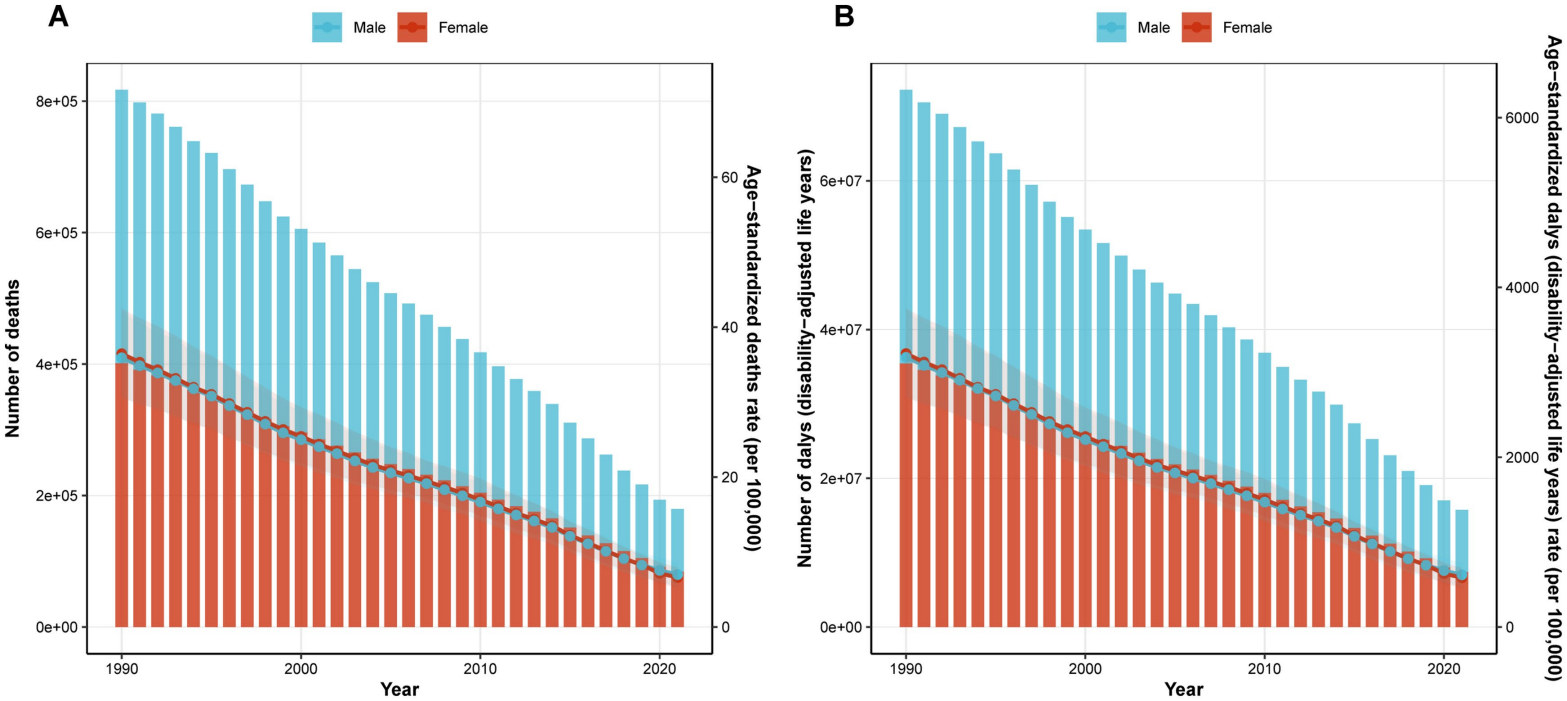
Trends in ASMR **(A)** and ASDR **(B)** of pneumococcal disease among individuals aged <20 years globally from 1990 to 2021.

From 1990 to 2021, the global pneumococcal burden among children and adolescents aged <20 years continued to decrease (Figure 5). The EAPC of the ASMR was −4.89% (95% *CI*: −5.23 to −4.54), decreasing from 36.18 per 100,000 (95% *UI*: 31.10–41.73) in 1990 to 6.80 per 100,000 (95% *UI*: 5.40–8.24) in 2021. Similarly, the EAPC of the ASDR was −4.90% (95% *CI*: −5.25 to −4.55), dropping from 3,198.28 per 100,000 (95% *UI*: 2,751.81–3,686.57) to 597.83 per 100,000 (95% *UI*: 474.25–724.18) (Table 1).

**Figure 6:**
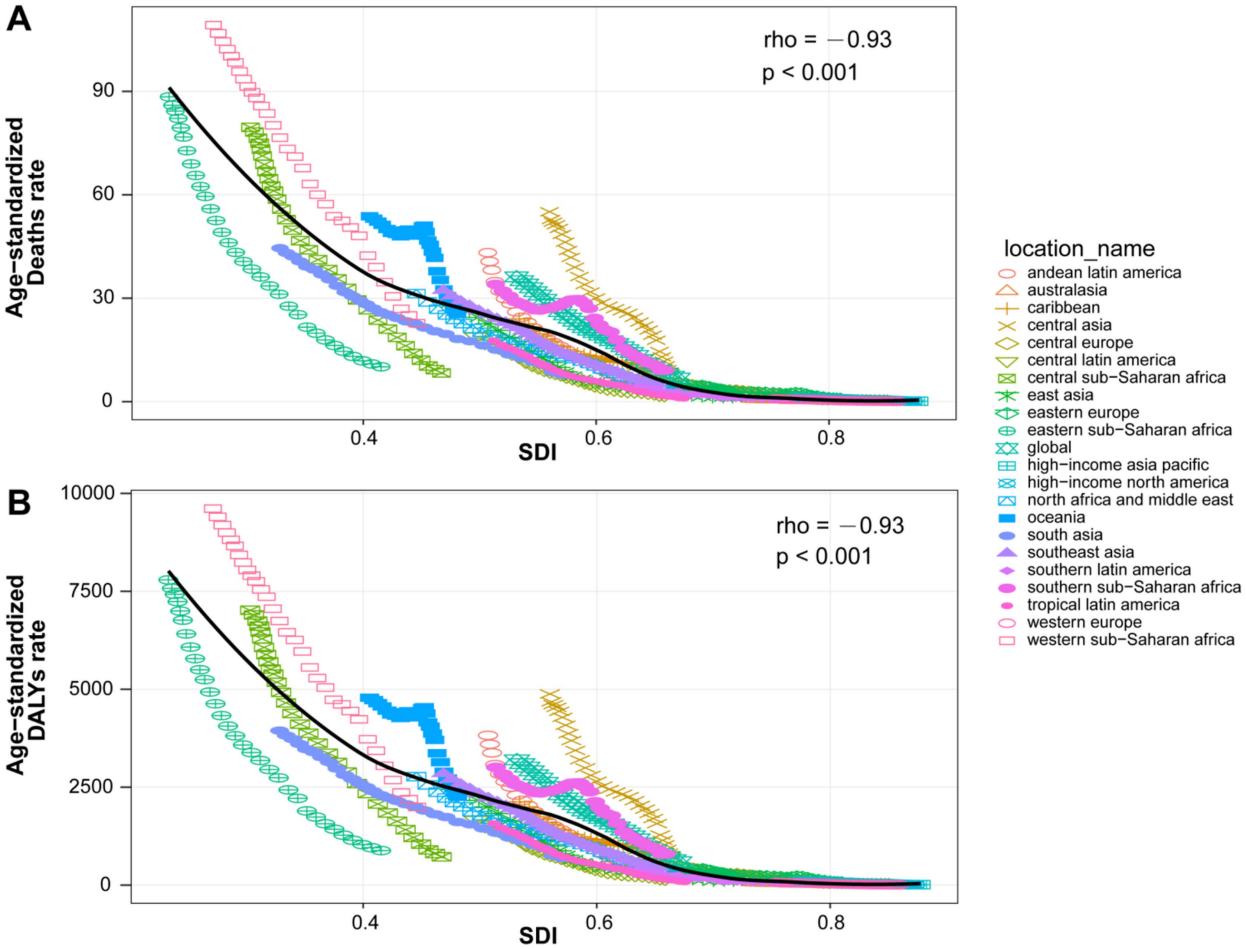
Correlations between the ASMRs **(A)** and ASDRs **(B)** of pneumococcal disease and the SDI across 21 regions from 1990 and 2021.

Regional analysis revealed the largest decline in burden in high-middle-SDI regions, with EAPCs of −8.63% (95% *CI*: −8.77 to 8.48) for the ASMR and −8.66% (95% *CI*: −8.80 to 8.51) for the ASDR (Supplementary Figure S3). Spearman’s correlation analysis revealed a significant negative correlation between the ASMR (*ρ* = −0.93, *p* < 0.001) and the ASDR (*ρ* = −0.93, *p* < 0.001) with the SDI across the 21 geographic regions (Figure 6). Overall, improvements in the SDI were closely related to reductions in disease burden.

Among regions, East Asia showed the most significant decrease, with EAPCs for the ASMR and ASDR of −10.33% (95% *CI*: −10.60 to −10.06) and −10.34% (95% *CI*: −10.61 to −10.06), respectively. In contrast, Oceania had the smallest reduction, with an EAPC of −2.20% for both (Supplementary Figure S4; Table 1). This difference suggests disparities in intervention coverage and health system performance across regions.

**Figure 7:**
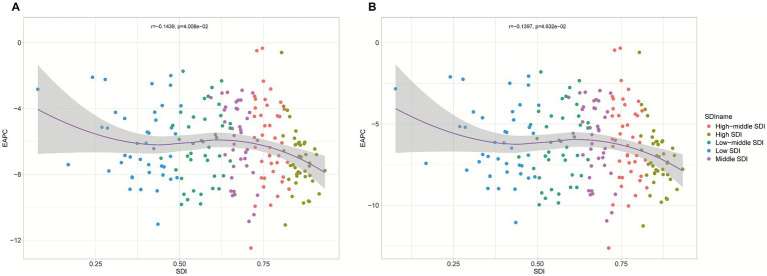
Estimated annual percentage change (EAPCs) in the ASMRs **(A)** and ASDRs **(B)** of pneumococcal disease in relation to the SDI across 204 countries from 1990 to 2021.

**Figure 8:**
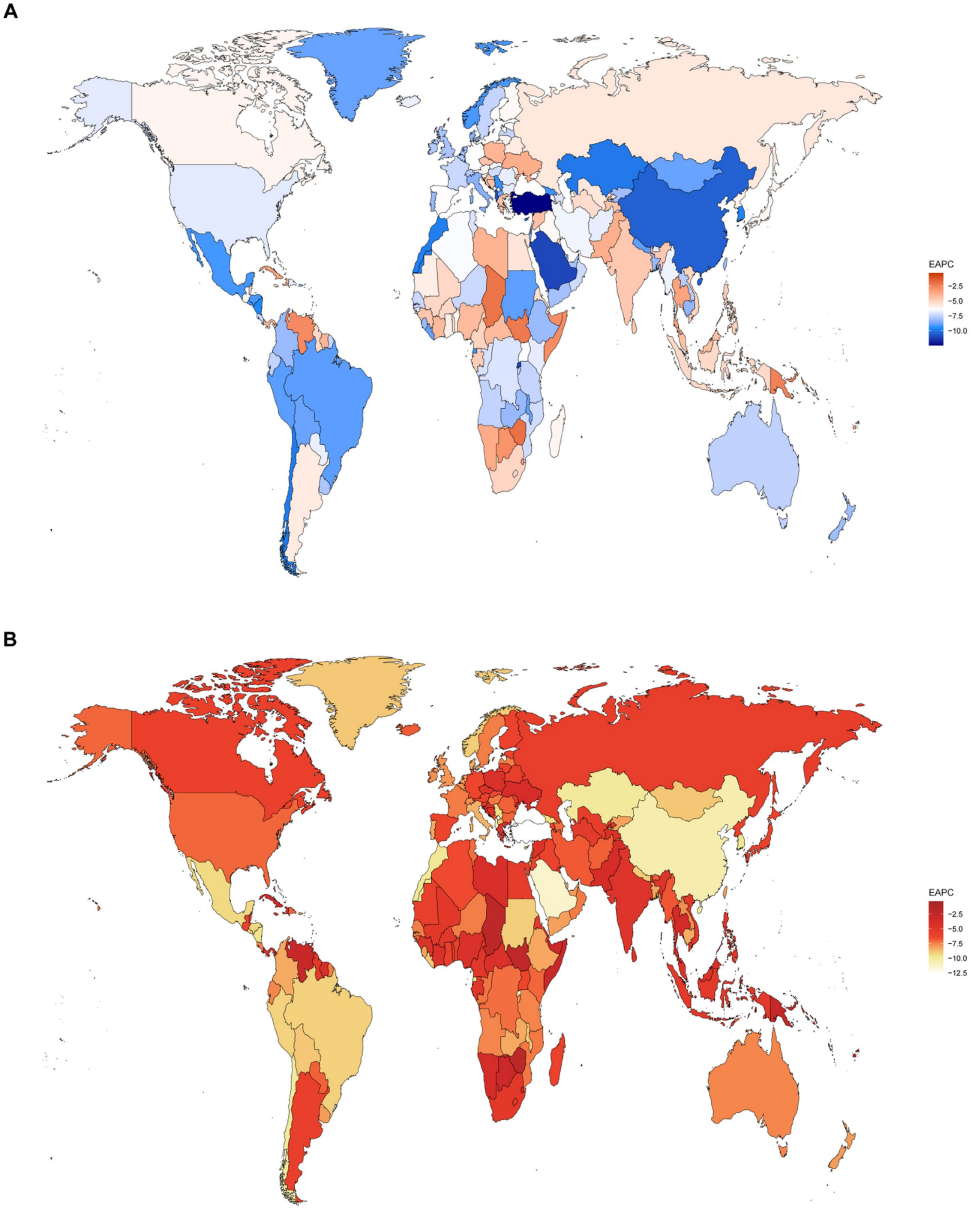
EAPCs in the ASMRs **(A)** and ASDRs **(B)** of pneumococcal disease among 204 countries from 1990 to 2021.

Further analysis of 204 countries and regions revealed a nonlinear relationship between the EAPC and the SDI, where the burden decrease accelerated as the SDI increased when it was below 0.25, fluctuated when the SDI ranged from 0.25 to 0.75, and accelerated again when the SDI exceeded 0.75 (Figure 7). Turkey showed the fastest decline, with EAPCs for the ASMR and ASDR of −12.48% (95% *CI*: −12.97 to −11.99) and −12.63% (95% *CI*: −13.12 to −12.13), respectively. Dominica had the slowest decline, with EAPCs of −0.33% (95% *CI*: −0.71 to 0.05) and −0.34% (95% *CI*: −0.72 to 0.05) (Figure 8; Supplementary Table S2).

Age stratification revealed the most significant decline in the burden for children aged <5 years, with an EAPC of −5.02% (95% *CI*: −5.40 to −4.63) for both the ASMR and ASDR (Supplementary Figure S5; Supplementary Table S3). Gender stratification revealed that the decline was slightly greater in females than in males, suggesting more significant improvements in this group during the study period (Supplementary Table S1). These trends highlight substantial differences in burden reduction across regions and groups.

### 3.3 Global and regional decomposition analysis

The decomposition analysis from 1990 to 2021 revealed that epidemiological changes were the primary drivers of burden reduction at both the global and regional levels. On a global scale, epidemiological improvements led to a significant 131.28% reduction in mortality and a 131.12% reduction in DALYs. However, population growth significantly offset these reductions, contributing to a 38.75% increase in mortality and a 38.63% increase in DALYs. In comparison, the effect of aging was relatively small, contributing a −7.46% reduction in mortality and a −7.51% reduction in DALYs.

**Figure 9:**
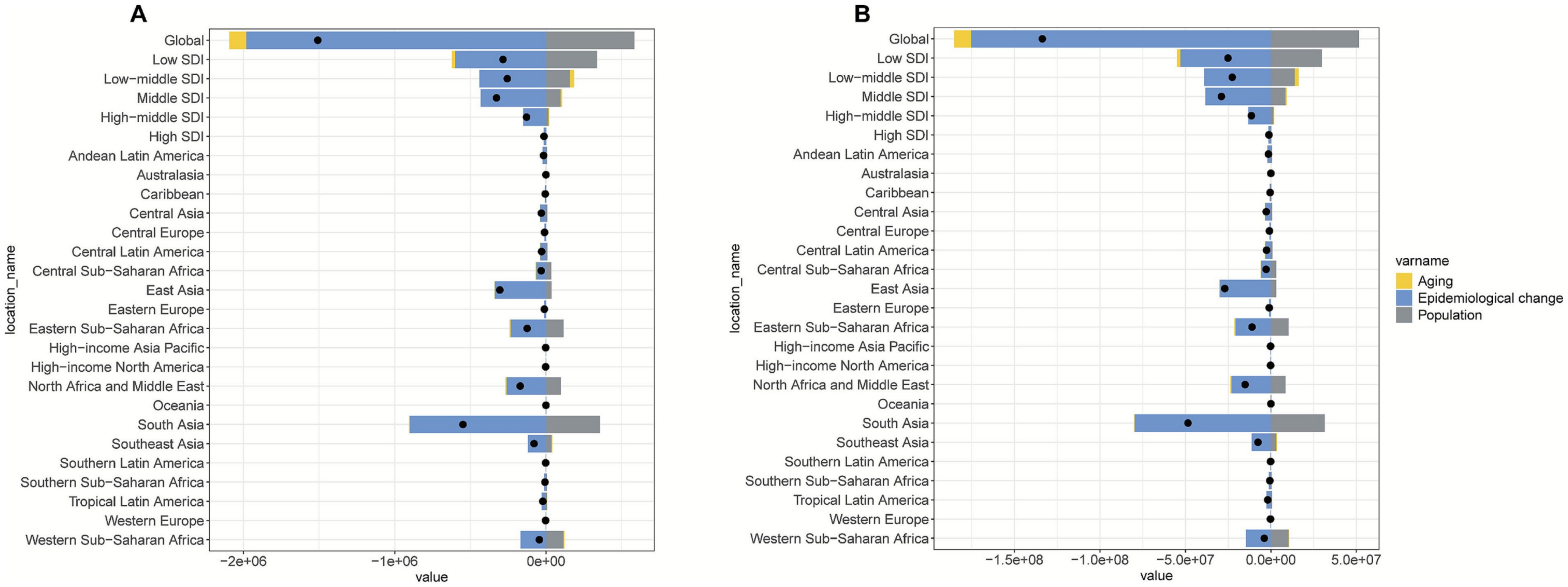
Contributions of population growth, population aging, and epidemiological changes to reductions in mortality **(A)** and DALYs **(B)** due to pneumococcal disease among individuals aged <20 years from 1990 to 2021.

At the regional level, the largest reductions were observed in low-SDI regions and South Asia. In low-SDI regions, epidemiological changes led to a remarkable 211.18% reduction in mortality and a 210.75% reduction in DALYs. Similarly, in South Asia, epidemiological changes resulted in a 164.03% reduction in mortality and a 164.09% reduction in DALYs. These regional findings emphasize that epidemiological changes have played a central role in driving the most substantial reductions in disease burden, both globally and regionally (Figure 9).

Gender stratification analysis revealed that epidemiological changes caused male mortality to decrease by 131.37% and DALYs to decrease by 131.21%, while for females, epidemiological changes resulted in a 131.17% reduction in mortality and a 131.02% reduction in DALYs. The impact of population growth was similar for both genders, with no significant gender differences in how population growth influenced disease burden (Supplementary Figure S6). These findings underscore the significant role of epidemiological improvements in reducing the disease burden across both sexes.

### 3.4 Health inequality analysis

**Figure 10:**
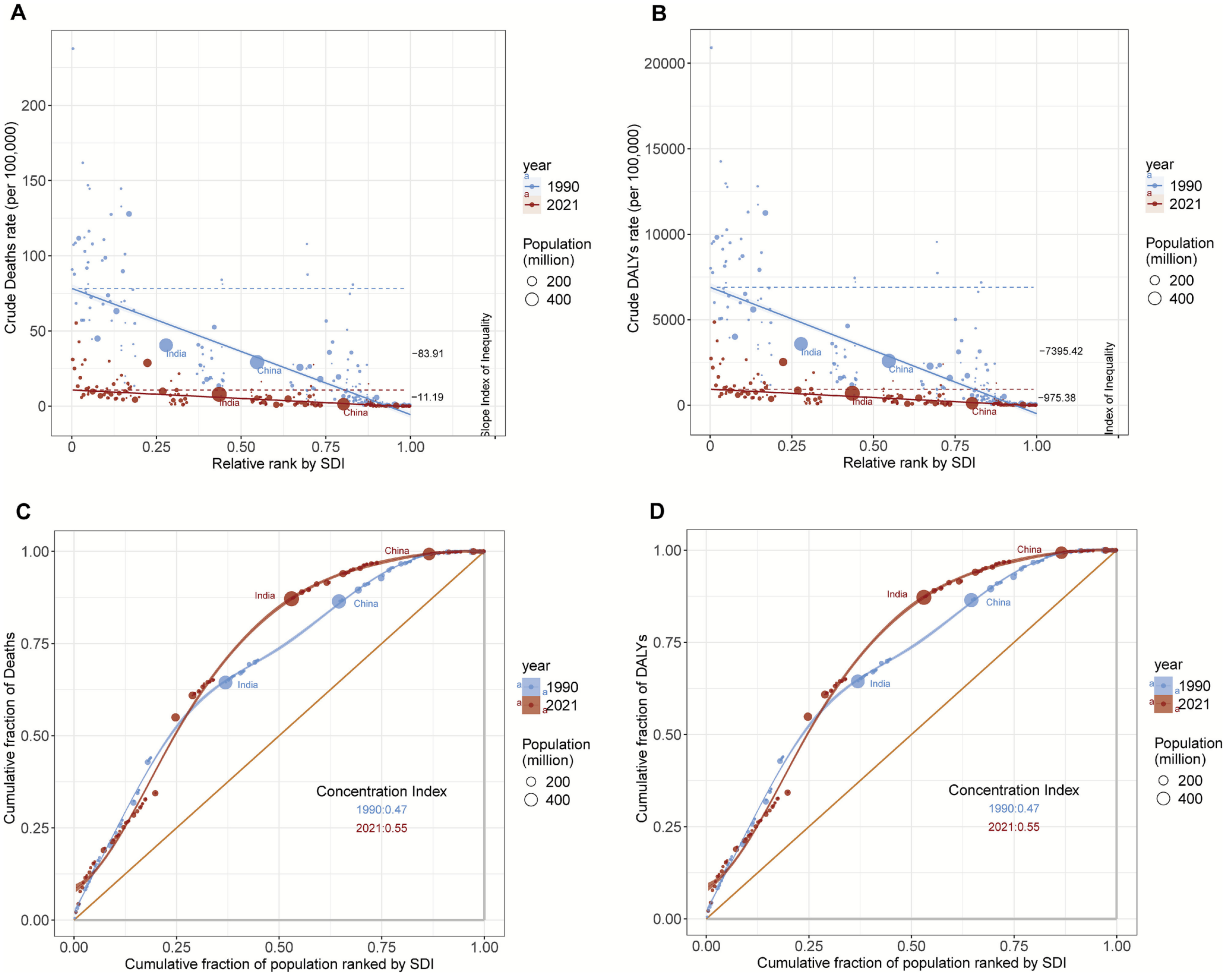
Changes in the slope index of inequality (SII) for the ASMR **(A)** and ASDR **(B)** and the concentration index **(CI)** for the ASMR **(C)** and ASDR **(D)** of pneumococcal disease from 1990 to 2021.

Given the significant correlation between SDI and pneumococcal mortality and DALYs (Figure 6; Supplementary Figure S2), we further analyzed global health inequality related to pneumococcal infections from 1990 to 2021. The SII was used to assess absolute inequality, whereas the CI was used to measure relative inequality. The results revealed that absolute health inequality improved from 1990 to 2021. The SII for the ASMR decreased from −83.91 to −11.19, and the SII for the ASDR decreased from −7,395.42 to −975.38, indicating a greater reduction in the absolute burden for low-SDI countries. However, the CI for both the ASMR and ASDR increased from 0.47 to 0.55, suggesting that the relative proportion of the burden in high-SDI countries increased. This may be due primarily to the greater reduction in burden in low-SDI countries, resulting in a greater relative share of remaining cases in high-SDI countries. Additionally, improvements in burdens within high-SDI countries were limited for immigrant children and low-income families (Figure 10).

### 3.5 Frontier analysis

**Figure 11:**
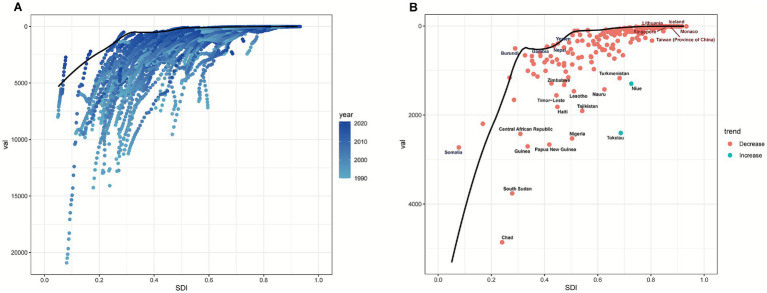
Frontier efficiency analysis of the ASDR and SDI among 204 countries from 1990 to 2021. **(A)** Distribution of countries on the ASDR-SDI frontier. **(B)** ASDR-SDI frontier in 2021 and potential improvement space. The black dots indicate countries with the largest efficiency gaps, the blue dots indicate countries with low SDIs and low gaps, and the red dots indicate countries with high SDIs and high gaps.

On the basis of the ASMR and ASDR of pneumococcal disease from 1990 to 2021 across 204 countries and territories, frontier efficiency analysis revealed that disparities in disease burden gradually decreased as SDI levels increased (Figure 11; Supplementary Figure S7). The results were consistent for both the ASMR and ASDR, with the 15 countries and territories showing the largest efficiency gaps being Chad, South Sudan, Guinea, Papua New Guinea, the Central African Republic, Nigeria, Haiti, Tokelau, Tajikistan, Timor-Leste, Lesotho, Nauru, Zimbabwe, Turkmenistan, and Niue. The boundary countries with low SDI values (<0.5) included Somalia, Burundi, Gambia, Nepal, and Yemen, indicating substantial potential for improvement even under resource-limited conditions. Notably, countries and regions with high SDIs (>0.85) but still significant potential for improvement included Singapore, Taiwan (China), Lithuania, Iceland, and Monaco. This may reflect disparities in the coverage or monitoring of high-risk populations within some high-income countries.

### 3.6 BAPC burden prediction

**Figure 12:**
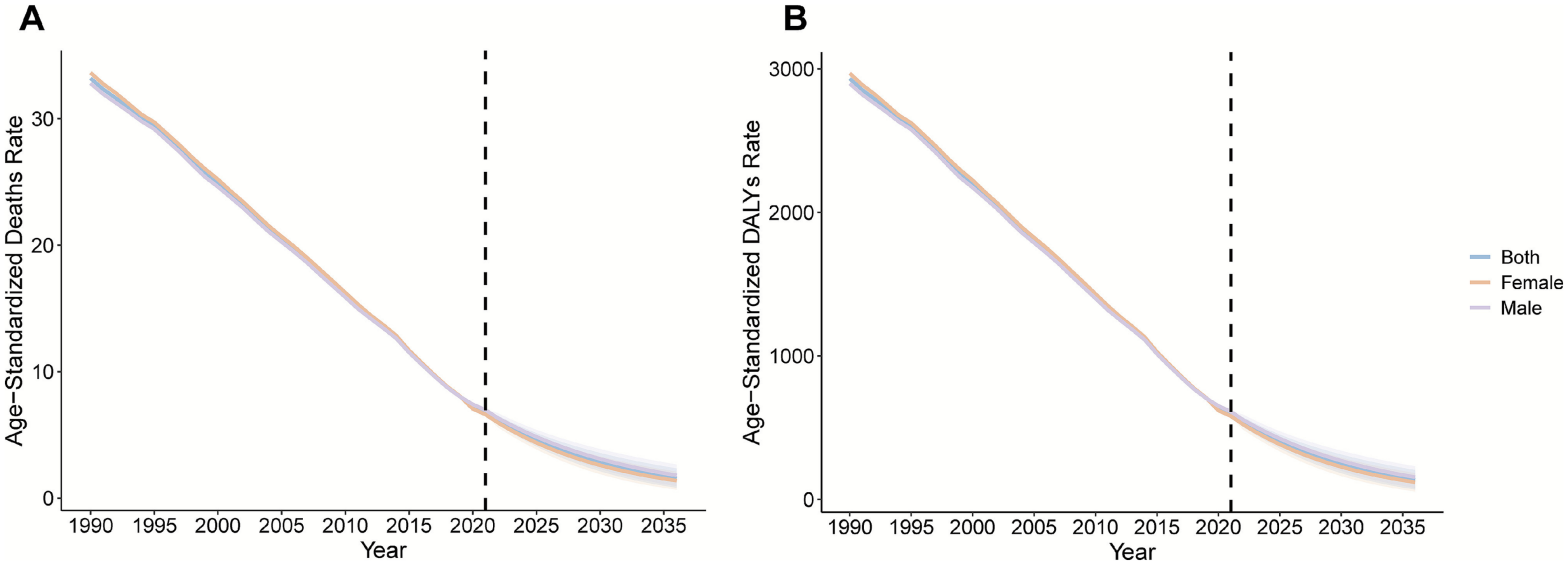
Projected trends in the ASMRs **(A)** and ASDRs **(B)** of pneumococcal disease among individuals aged <20 years globally from 2022 to 2036 based on the Bayesian age-period-cohort (BAPC) model.

Model projections show that the global pneumococcal burden in children and adolescents will continue to decrease from 2022 to 2036 (Figure 12). By 2036, the estimated ASMR is expected to decrease to 1.59 per 100,000 (95% *UI*: 0.82–2.35), and the ASDR will decrease to 137.37 per 100,000 (95% *UI*: 69.41–205.33). Stratified by sex, the burden for males is projected to remain greater than that for females. The male ASMR is projected to be 1.80 per 100,000 (95% *UI*: 0.95–2.66), and for females, it will be 1.38 per 100,000 (95% *UI*: 0.67–2.09). The male ASDR will be 155.58 per 100,000 (95% *UI*: 79.99–231.17), and the female ASDR will be 120.10 per 100,000 (95% *UI*: 56.14–184.06) (Supplementary Table S4). These projections should be interpreted in the context of future vaccine strategies and changes in healthcare systems.

## 4 Discussion

This study systematically evaluated the spatiotemporal changes in the global, regional, and national pneumococcal burdens among children and adolescents aged <20 years from 1990 to 2021 and predicted future trends through 2036 via the BAPC model. Although there has been a significant decline in global pneumococcal-related mortality and DALYs over the past 30 years, the absolute level of the disease burden remains high, especially in countries with low SDI values and among children aged <5 years. Compared with previous studies, this research further extends our understanding of the long-term evolution of pneumococcal disease under the global burden of disease (GBD) framework and highlights the continuing major challenge it poses to child health.

This study revealed that from 1990 to 2021, the global ASMR and ASDR for pneumococcal infections in children and adolescents decreased by approximately 81%, which is consistent with trends observed in GBD 2019, and the World Health Organization (WHO) reported a decline in childhood pneumonia mortality (5). Several studies suggest that the significant decline in the disease burden is closely related to the widespread introduction of the pneumococcal conjugate vaccine (PCV), improved access to antimicrobial treatments, better nutrition, and comprehensive child healthcare interventions (24, 25). However, the ASMR in low-SDI countries was significantly higher than the global average. In addition to insufficient vaccine coverage and weak healthcare systems, structural barriers, such as vaccine cold chain disruptions, heavy reliance on external aid, community hesitancy about vaccine safety, and conflicts and humanitarian crises that disrupt vaccination services, may limit the sustained reduction in disease burden in these countries (26, 27). In contrast, high-SDI countries have advanced across key domains, leading to a reduction in pneumococcal burden. A key factor in this progress is the expansion of vaccine coverage, which has proven pivotal in preventing pneumococcal infections, thereby significantly lowering both mortality rates and DALYs (28). Equally important are the robust health systems in these countries, underpinned by strong infrastructure, skilled professionals, and equitable access to medical services, all of which facilitate timely diagnosis, treatment, and management of pneumococcal disease (29). Adequate nutrition further strengthens host immunity, reducing the severity and frequency of infections, particularly in children and adolescents (30). In addition, broader access to effective antimicrobial therapies and specialized pediatric care plays a crucial role in further alleviating the disease burden (31).

This study also revealed significant sex differences. In 2021, male children and adolescents had higher mortality rates and DALYs than females did, and this difference is expected to persist until 2036. Previous studies have shown that male infants exhibit increased morbidity and mortality rates in respiratory infections, possibly due to delayed immune system maturation, respiratory tract anatomical features, and hormonal differences (32, 33). In addition to biological mechanisms, potential delays in medical care and cultural gender preferences in some regions may make boys more likely to develop severe illness (34). Cohort studies in South Asia and Sub-Saharan Africa suggest that male infants often experience more severe disease and later-stage hospitalizations, which may be closely related to family factors, such as early disease recognition, healthcare decision-making, and caregiving (35). Compared with other respiratory pathogens, such as respiratory syncytial virus and influenza virus, the direction and magnitude of sex differences exhibit similar patterns, indicating a cross-pathogen characteristic of pediatric infectious diseases (36). Future research could further validate the impact mechanisms of healthcare delays, healthcare behaviors, and gender biases through household-level tracking data and health utilization surveys in different cultural and economic contexts and explore customized preventive interventions for high-risk male infant populations.

Health inequality analysis reveals that, although absolute disparities between countries have decreased, this reduction is primarily attributed to progress in low-SDI countries, facilitated by international aid, global vaccination programs, and the expansion of basic health services (37, 38). However, pneumococcal disease burden has become increasingly concentrated in high-SDI countries. This trend may be attributed to the faster reduction in disease burden in low-SDI countries, resulting in a rise in relative inequality, as a greater proportion of remaining cases are now concentrated in high-SDI countries (39). Furthermore, the heightened sensitivity of death registration and monitoring systems in high-income countries, compared to low-SDI countries, may further contribute to the increase in the CI (7, 40). Some studies suggest that vulnerable populations in high-SDI countries, such as immigrant children, ethnic minorities, and low-income families, have not benefited proportionally from health interventions, thus “inflating” their relative contribution to global disease burden estimates. These groups encounter barriers, including cultural, linguistic, and economic challenges, that prevent them from fully benefiting from health interventions (41). Despite the critical role of global vaccination initiatives and international aid in reducing the burden in low-SDI countries, health inequalities within high-SDI countries remain significant, with the needs of marginalized populations still inadequately addressed. This suggests that current policies still lack targeted interventions for these groups in high-SDI countries. Therefore, future policies must prioritize the inclusion of these marginalized groups to ensure equitable benefits from global health programs.

Frontier analysis further indicates that although high-SDI countries such as Singapore, Lithuania, and Iceland generally have low burden levels, they still exhibit a substantial gap from the theoretical minimum burden. This gap suggests that in high-income countries, factors such as vaccine hesitancy, declining vaccination compliance, vaccination gaps among immigrant populations, and an increasing proportion of children with chronic underlying conditions may act as bottlenecks for further reducing the disease burden (42, 43). While Singapore has an immunization coverage rate close to 90%, the vaccination rate among foreign workers and children from low-income immigrant families has consistently been much lower than that among local residents (44). In the Middle East and North Africa, migration and refugee movements have led to the exclusion of some communities from national immunization systems (45). To address the residual burden in these high-SDI countries, future efforts should focus on strengthening community health education, culturally adapted communication, targeted catch-up vaccination, and follow-up services, especially those focused on immigrant children and minority groups. Through the precise identification of high-risk populations and the reduction of information barriers, further reductions in potentially preventable burdens can be achieved.

Moreover, the predictive analysis in this study revealed that by 2036, the global ASMR is expected to decrease to 1.59 per 100,000, and the ASDR is expected to decrease to 137.37 per 100,000, with a continuing downward trend. This reaffirms the effectiveness of current prevention measures. However, it should be noted that the BAPC model extrapolates on the basis of historical trends, and future developments such as the continued evolution of serotype replacement, intensification of antibiotic resistance, fluctuations in global vaccine supply and demand, and the incorporation of next-generation vaccines such as PCV20 and PPSV23 into national immunization programs may have profound impacts on these projected trends (46–48). Previous studies have shown that in some middle- and high-income countries, serotype replacement has led to a rebound in invasive pneumococcal disease burden 5–10 years after vaccine introduction, and the dynamics of resistance transmission could also alter the effectiveness of antimicrobial treatments (49). Additionally, uncertainties around the cost, supply chain, and coverage speed of new-generation vaccines directly affect accessibility in middle- and low-income countries, which could impact global burden reduction (50). Therefore, future research should integrate serotype replacement simulations, vaccine strategy modeling, and resistance monitoring results to dynamically assess the long-term evolution of disease burden under various scenarios, providing scientific support for evidence-based and sustainable immunization programs in different countries.

This study, which is based on the GBD 2021 database, covers over 30 years of time series data and 204 countries, ensuring the global representativeness of the estimates. The use of multiple methods, including EAPC trend estimation, decomposition analysis, health inequality assessment, frontier efficiency analysis, and Bayesian prediction, provides a comprehensive analysis of disease burden changes from different perspectives, with a particular focus on children and adolescents, a high-risk population. However, there are limitations in this study. The GBD estimates rely on data from various sources, and some low-income countries have limited monitoring data, which may introduce bias. Age stratification was not further refined to the month level, potentially underestimating the heterogeneity in the burden among infants and toddlers. While the BAPC model is stable, it has limited sensitivity to future policy changes, serotype replacement, and the introduction of new vaccines, and the prediction results should be interpreted with caution, particularly in scenario analyses.

Based on our findings, several key policy interventions are critical to reducing the global pneumococcal disease burden, particularly among high-risk populations. Ensuring universal vaccination coverage is fundamental to pneumococcal disease prevention, particularly through catch-up immunization for children aged <5 years in low-SDI regions with historically low vaccination rates. Such programs can reduce pneumococcal mortality by improving vaccination coverage among under-immunized populations. Strengthening serotype and antibiotic resistance surveillance is essential for adapting vaccination strategies to emerging strains and ensuring their long-term effectiveness, thereby further reducing mortality rates. In high-SDI countries, addressing health inequities among marginalized populations, including immigrants and socioeconomically disadvantaged groups, is critical, as these populations face significant barriers to healthcare access, exacerbating both disease burden and mortality. Implementing these interventions, as emphasized by our findings, has the potential to significantly reduce pneumococcal mortality and promote global health equity.

Overall, this study revealed that while the pneumococcal disease burden has significantly declined over the past 30 years, it remains a major threat to the health of children in low-SDI countries and among children aged <5 years. Global health actions should continue to focus on improving vaccine accessibility while addressing structural barriers such as cold chain support, financial sustainability, vaccine hesitancy, and interventions in conflict zones to reduce both relative and absolute inequalities and help achieve the global goal of significantly reducing preventable child deaths.

## 5 Conclusion

Over the past 30 years, the global pneumococcal burden among children and adolescents under 20 has significantly decreased. However, the mortality rate and DALYs remain high in low-SDI countries, and relative inequalities have increased. Moving forward, it is crucial to continue expanding high-quality vaccination coverage, strengthen healthcare system resilience, and implement targeted interventions for high-risk populations to further reduce the burden of pneumococcal disease.

## Data Availability

The datasets presented in this study can be found in online repositories. The names of the repository/repositories and accession number(s) can be found in the article/Supplementary material.

## Footnotes

1
http://ghdx.healthdata.org/gbd-2021
