# Supplementary material for: Global, regional, and national burden of pneumococcal disease among children and adolescents aged <20 years from 1990 to 2021: a predictive analysis
Source: Front Public Health. 2025 Dec 5;13:1675854. doi: 10.3389/fpubh.2025.1675854 (PMC12714602; doi:10.3389/fpubh.2025.1675854)
Supplement: Supplementary file 1 [file Data_Sheet_1.docx]

Supplementary Material

# Supplementary Figures and Tables

## Supplementary Figures

**
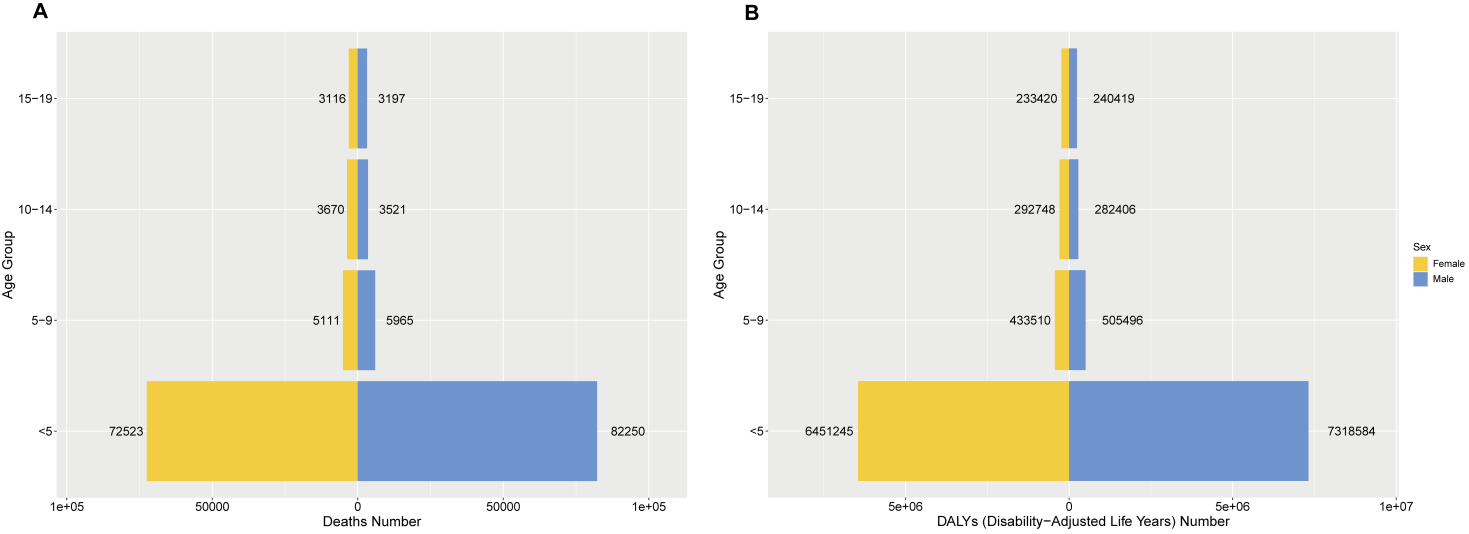
**

**Supplementary Figure S1. Sex and age distributions of deaths (A) and DALYs (B) due to pneumococcal disease among individuals aged <20 years globally in 2021.**

**
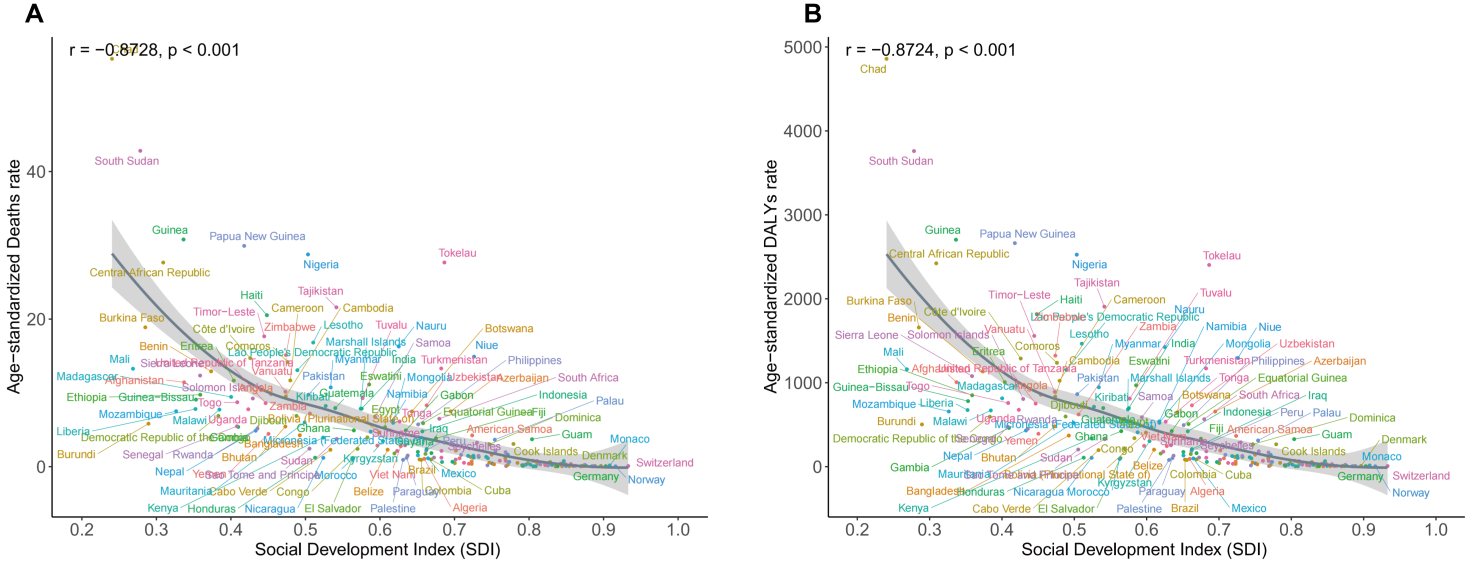
**

**Supplementary Figure S2. Associations between the SDI and the ASMRs (A) and ASDRs (B) of pneumococcal disease among individuals aged <20 years in 2021 across 204 countries.**

**
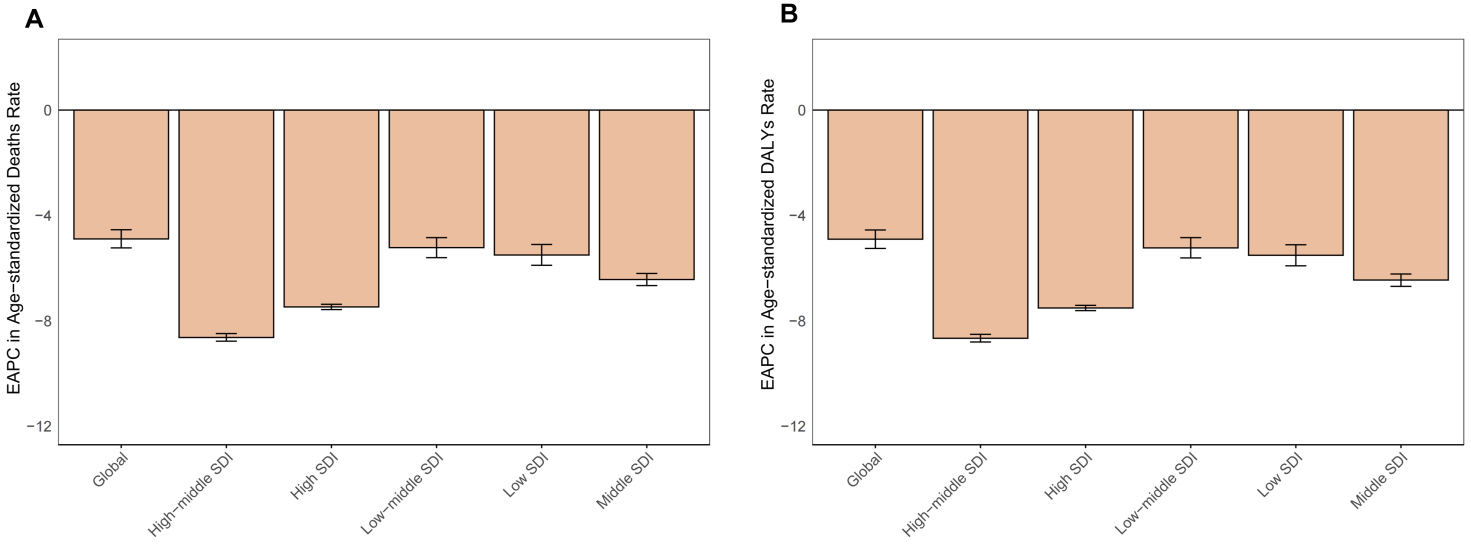
**

**Supplementary Figure S3. EAPCs in the ASMRs (A) and ASDRs (B) of pneumococcal disease from 1990-2021 by SDI region.**

**
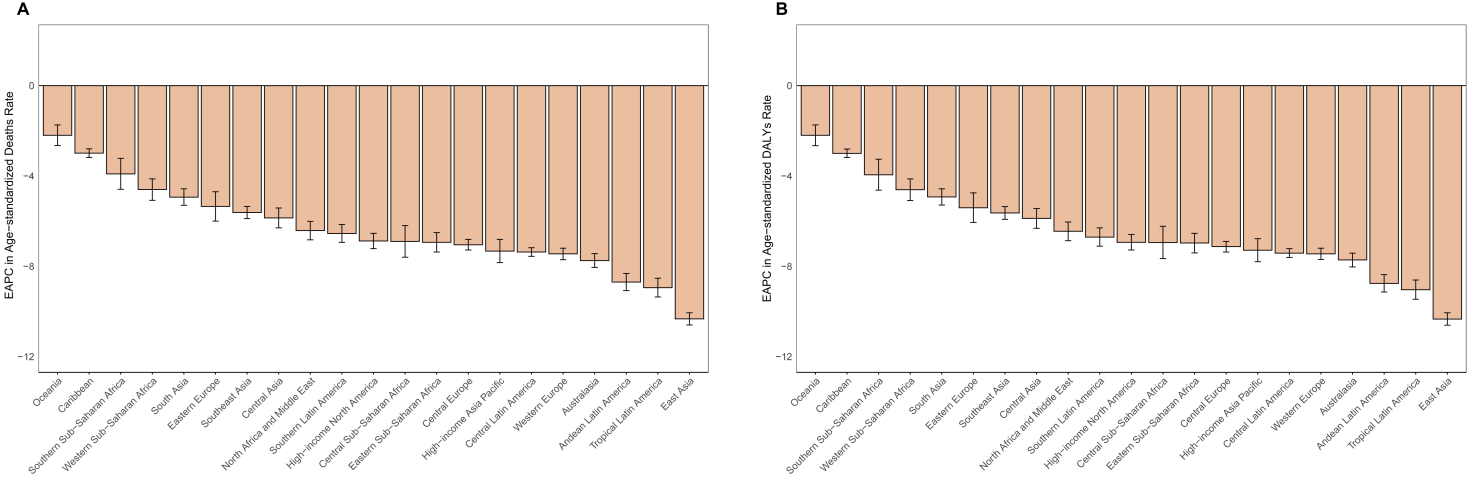
**

**Supplementary Figure S4. EAPCs in the ASMRs (A) and ASDRs (B) of pneumococcal disease from 1990-2021 across 21 global regions.**

**
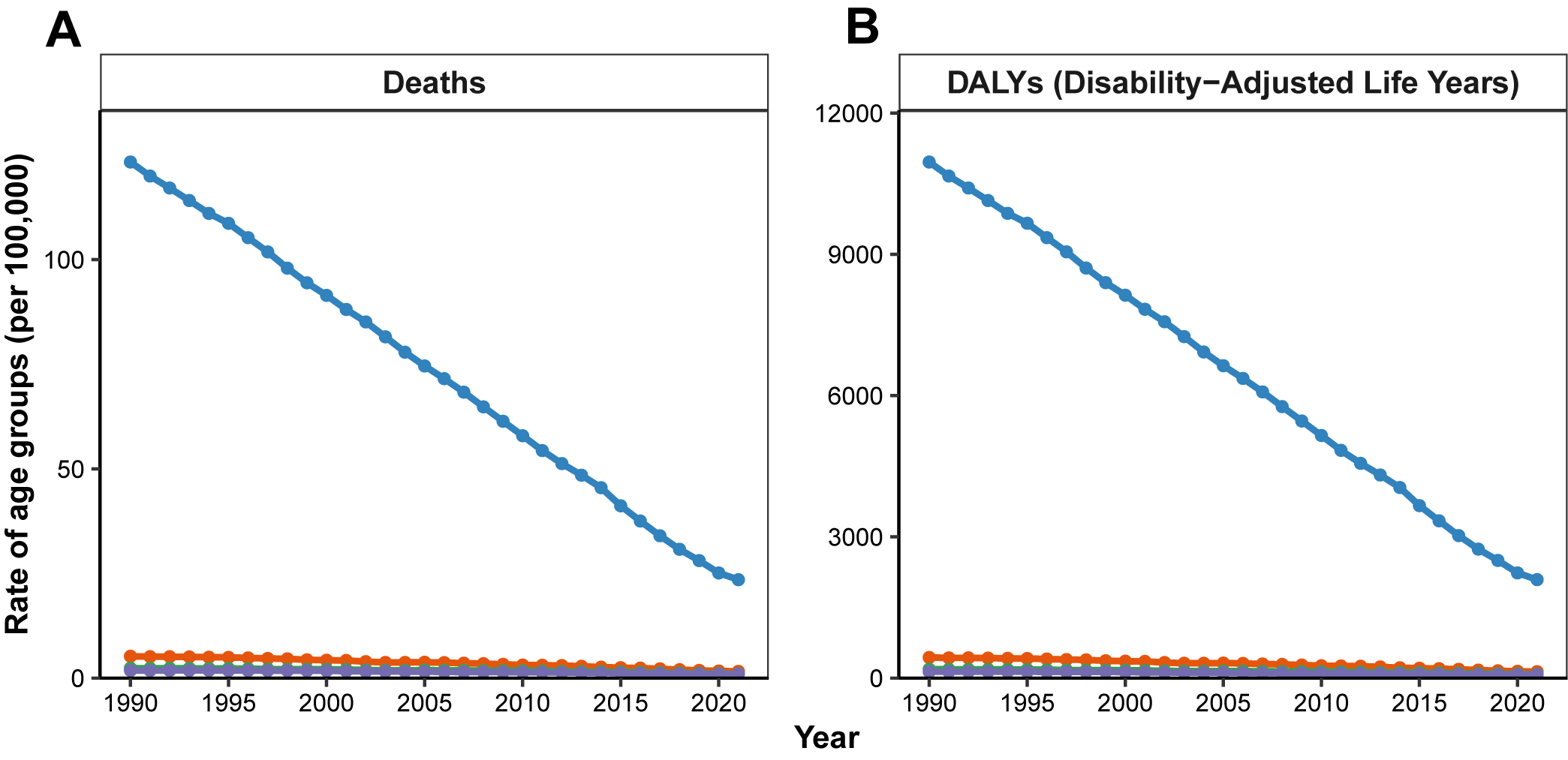
**

**Supplementary Figure S5.** **Trends in the ASMRs (A) and ASDRs (B) of pneumococcal disease by age group (<20 years) from 1990-2021.**

**
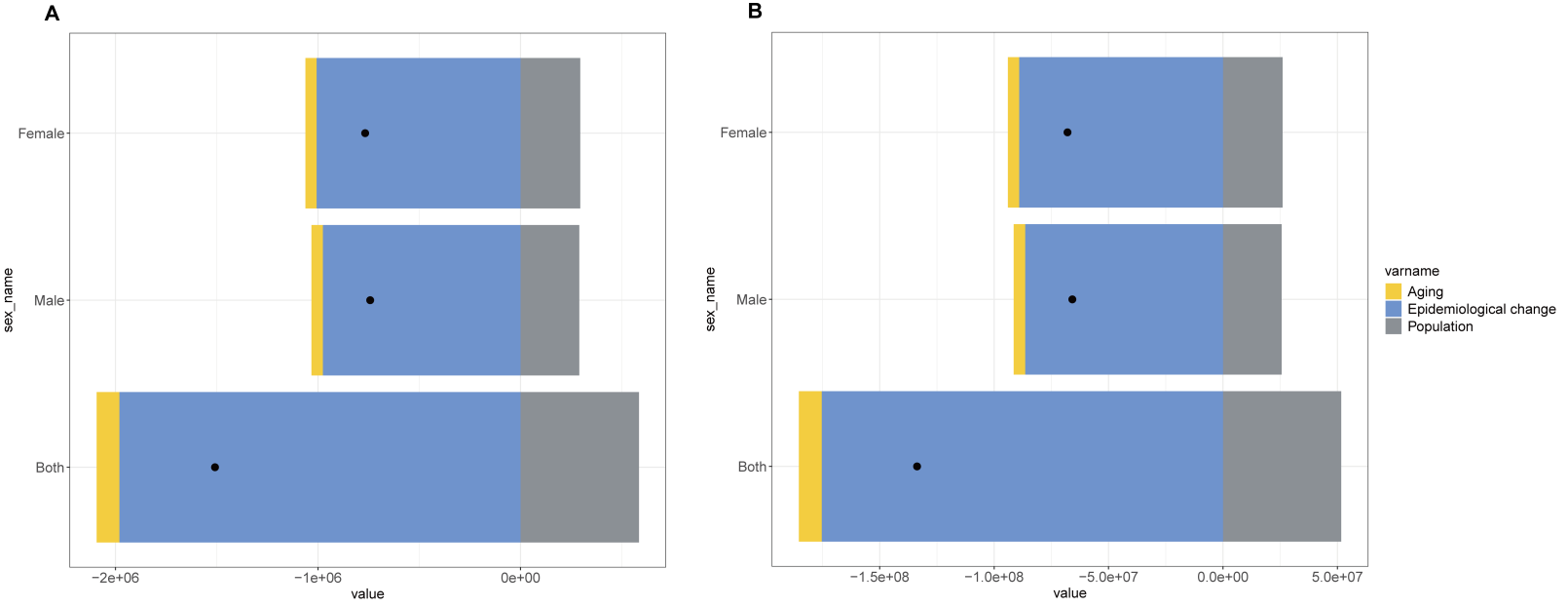
**

**Supplementary Figure S6. Decomposition of contributing factors for the reduction in deaths (A) and DALYs (B) due to pneumococcal disease among individuals aged <20 years globally by sex from 1990-2021.**

**
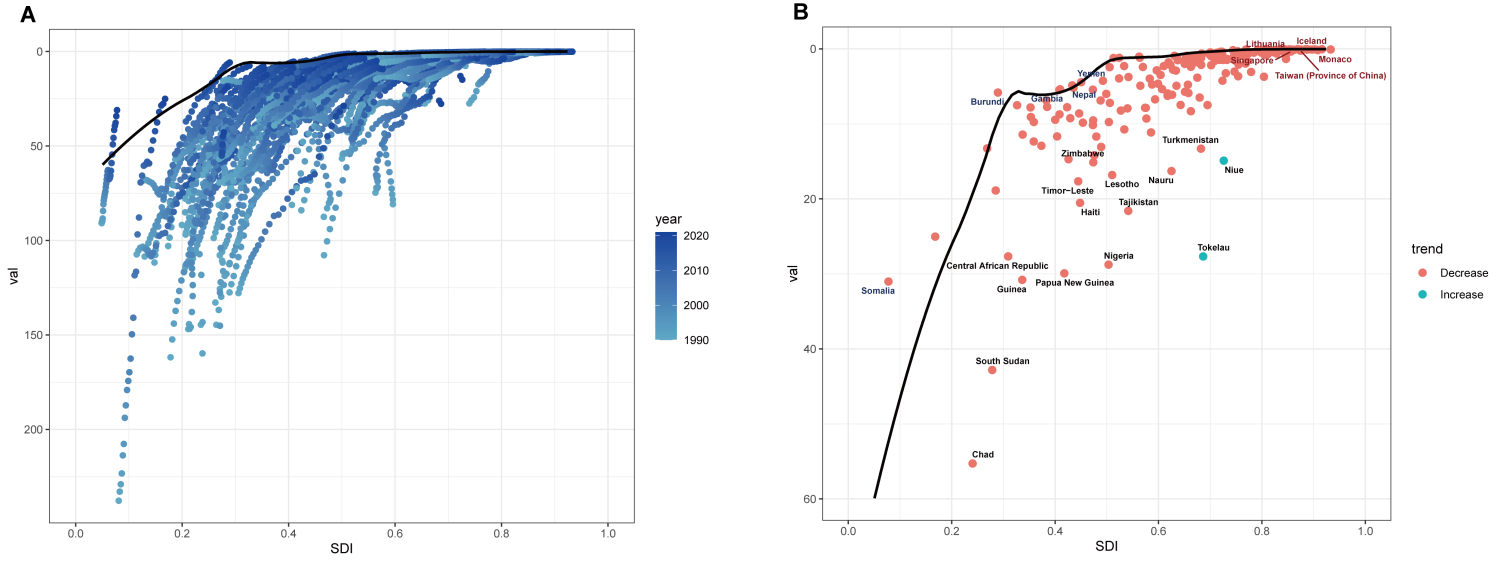
**

**Supplementary Figure S7**. **Frontier efficiency analysis of the ASMRs and SDIs among 204 countries from 1990-2021.** (A) Distribution of countries on the ASMR-SDI frontier. (B) ASMR-SDI frontier in 2021 and potential improvement space. The black dots indicate countries with the largest efficiency gaps, the blue dots indicate countries with low SDIs and low gaps, and the red dots indicate countries with high SDIs and high gaps.

## Supplementary Tables

**Supplementary Table S1. Global deaths and DALYs from pneumococcal disease among children and adolescents aged <20 years by sex in 1990 and 2021, including counts, age-standardized rates per 100,000 population, and EAPCs.**

|  | **1990** | | **2021** | |  |
| --- | --- | --- | --- | --- | --- |
| **location** | **Counts (95% *UI*)** | **ASR per 100,000 (95% *UI*)** | **Counts (95% *UI*)** | **ASR per 100,000 (95% *UI*)** | **EAPC_95%*CI*** |
| **Deaths** |  |  |  |  |  |
| Both | 817218 (702428 to 942591) | 36.18 (31.10 to 41.73) | 179354 (142347 to 217280) | 6.80 (5.40 to 8.24) | -4.89 (-5.23 to 4.54) |
| Female | 401456 (336533 to 467187) | 36.46 (30.56 to 42.43) | 84421 (67891 to 100800) | 6.61 (5.32 to 7.89) | -4.92 (-5.30 to 4.55) |
| Male | 415761 (354282 to 486929) | 35.92 (30.61 to 42.07) | 94933 (74125 to 116417) | 6.99 (5.46 to 8.57) | -4.85 (-5.18 to 4.53) |
| **DALYs** |  |  |  |  |  |
| Both | 72235581 (62151695 to 83264012) | 3198.28 (2751.81 to 3686.57) | 15757828 (12500395 to 19088138) | 597.83 (474.25 to 724.18) | -4.90 (-5.25 to 4.55) |
| Female | 35465175 (29754759 to 41274023) | 3220.60 (2702.03 to 3748.10) | 7410922 (5947266 to 8850289) | 580.18 (465.60 to 692.87) | -4.94 (-5.31 to 4.56) |
| Male | 36770406 (31342942 to 43042645) | 3177.05 (2708.10 to 3718.98) | 8346905 (6508339 to 10228242) | 614.42 (479.08 to 752.91) | -4.87 (-5.19 to 4.54) |
| The EAPC was calculated using 31 years of data from 1990-2021. The table only includes data for 1990 and 2021 for comparative analysis. UI, uncertainty interval. ASR, age-standardized rate per 100,000. EAPC, estimated annual percentage change of ASR. CI, confidence interval. | | | | | |

**Supplementary Table S2. Country-specific estimates of deaths and DALYs from pneumococcal disease among children and adolescents aged <20 years in 1990 and 2021, including counts, age-standardized rates per 100,000 population, and EAPCs.**

|  | **1990** | | **2021** | |  |
| --- | --- | --- | --- | --- | --- |
| **location** | **Counts (95% *UI*)** | **ASR per 100,000 (95% *UI*)** | **Counts (95% *UI*)** | **ASR per 100,000 (95% *UI*)** | **EAPC_95%*CI*** |
| **Deaths** |  |  |  |  |  |
| Afghanistan | 5737 (4294 to 7542) | 102.94 (77.05 to 135.32) | 2032 (1487 to 2736) | 11.43 (8.36 to 15.39) | -6.85 (-7.50 to -6.19) |
| Albania | 449 (376 to 525) | 31.08 (25.97 to 36.28) | 7 (5 to 9) | 1.13 (0.81 to 1.47) | -10.86 (-11.11 to -10.62) |
| Algeria | 1846 (1369 to 2624) | 13.66 (10.13 to 19.41) | 175 (137 to 227) | 1.07 (0.83 to 1.38) | -6.51 (-7.17 to -5.85) |
| American Samoa | 3 (2 to 3) | 10.76 (8.98 to 12.82) | 1 (1 to 1) | 4.25 (3.12 to 5.50) | -3.26 (-3.41 to -3.10) |
| Andorra | 0 (0 to 0) | 0.64 (0.44 to 0.92) | 0 (0 to 0) | 0.03 (0.02 to 0.04) | -9.74 (-10.28 to -9.20) |
| Angola | 6218 (4707 to 7748) | 107.84 (81.63 to 134.37) | 1837 (1263 to 2555) | 9.84 (6.76 to 13.68) | -7.55 (-8.22 to -6.87) |
| Antigua and Barbuda | 1 (1 to 1) | 2.61 (2.16 to 3.13) | 0 (0 to 0) | 1.62 (1.42 to 1.85) | -2.34 (-2.83 to -1.84) |
| Argentina | 664 (621 to 715) | 5.10 (4.76 to 5.49) | 98 (85 to 112) | 0.72 (0.62 to 0.82) | -5.77 (-6.27 to -5.27) |
| Armenia | 318 (281 to 365) | 23.97 (21.18 to 27.52) | 17 (13 to 21) | 2.18 (1.74 to 2.72) | -6.49 (-7.04 to -5.95) |
| Australia | 35 (32 to 38) | 0.67 (0.62 to 0.73) | 3 (3 to 4) | 0.06 (0.05 to 0.07) | -7.59 (-8.01 to -7.17) |
| Austria | 10 (9 to 10) | 0.52 (0.48 to 0.56) | 1 (1 to 1) | 0.07 (0.06 to 0.07) | -6.11 (-6.37 to -5.85) |
| Azerbaijan | 2529 (2171 to 2899) | 80.81 (69.35 to 92.61) | 228 (176 to 285) | 7.50 (5.77 to 9.36) | -7.04 (-7.49 to -6.59) |
| Bahamas | 5 (4 to 6) | 4.94 (4.15 to 5.97) | 1 (1 to 1) | 0.79 (0.61 to 1.05) | -6.09 (-6.61 to -5.56) |
| Bahrain | 12 (10 to 14) | 5.82 (4.89 to 7.13) | 1 (1 to 2) | 0.32 (0.27 to 0.38) | -8.23 (-8.58 to -7.88) |
| Bangladesh | 38157 (32111 to 44037) | 63.22 (53.20 to 72.96) | 2598 (2007 to 3312) | 4.26 (3.29 to 5.43) | -8.20 (-8.92 to -7.48) |
| Barbados | 2 (2 to 3) | 2.70 (2.27 to 3.16) | 0 (0 to 1) | 0.55 (0.39 to 0.76) | -5.62 (-6.38 to -4.85) |
| Belarus | 118 (101 to 138) | 3.77 (3.22 to 4.41) | 10 (8 to 13) | 0.51 (0.40 to 0.66) | -5.79 (-6.07 to -5.51) |
| Belgium | 22 (20 to 23) | 0.88 (0.82 to 0.95) | 2 (2 to 3) | 0.10 (0.08 to 0.11) | -7.85 (-8.18 to -7.52) |
| Belize | 14 (12 to 16) | 13.46 (12.02 to 15.24) | 4 (3 to 5) | 2.30 (1.88 to 2.77) | -5.76 (-6.16 to -5.37) |
| Benin | 2745 (2048 to 3626) | 95.66 (71.35 to 126.36) | 971 (607 to 1453) | 12.92 (8.08 to 19.33) | -6.12 (-6.67 to -5.57) |
| Bermuda | 0 (0 to 0) | 1.54 (1.29 to 1.81) | 0 (0 to 0) | 0.44 (0.32 to 0.55) | -4.10 (-4.36 to -3.84) |
| Bhutan | 152 (76 to 209) | 44.55 (22.40 to 61.34) | 14 (9 to 19) | 5.44 (3.68 to 7.44) | -6.91 (-7.22 to -6.59) |
| Bolivia (Plurinational State of) | 2814 (2040 to 3615) | 84.01 (60.92 to 107.95) | 211 (161 to 268) | 4.65 (3.53 to 5.90) | -8.86 (-9.26 to -8.46) |
| Bosnia and Herzegovina | 22 (17 to 28) | 1.51 (1.15 to 1.87) | 3 (2 to 3) | 0.38 (0.30 to 0.47) | -4.48 (-5.01 to -3.94) |
| Botswana | 162 (125 to 204) | 21.90 (16.90 to 27.59) | 69 (49 to 95) | 7.48 (5.29 to 10.41) | -3.01 (-3.70 to -2.33) |
| Brazil | 12053 (10826 to 13548) | 17.94 (16.11 to 20.16) | 620 (504 to 744) | 0.97 (0.79 to 1.16) | -9.02 (-9.43 to -8.60) |
| Brunei Darussalam | 3 (2 to 3) | 2.33 (1.96 to 2.75) | 1 (1 to 1) | 0.64 (0.49 to 0.83) | -3.86 (-4.01 to -3.71) |
| Bulgaria | 229 (214 to 245) | 9.70 (9.07 to 10.36) | 15 (12 to 17) | 1.15 (0.96 to 1.34) | -6.88 (-7.64 to -6.11) |
| Burkina Faso | 6138 (4494 to 7790) | 108.49 (79.44 to 137.70) | 2418 (1562 to 3317) | 18.88 (12.19 to 25.90) | -5.19 (-5.83 to -4.54) |
| Burundi | 2456 (1646 to 3208) | 77.84 (52.17 to 101.67) | 424 (264 to 624) | 5.83 (3.62 to 8.58) | -7.80 (-8.65 to -6.94) |
| Cabo Verde | 30 (23 to 40) | 15.53 (11.58 to 20.34) | 4 (3 to 6) | 2.29 (1.72 to 2.93) | -7.11 (-7.68 to -6.53) |
| Cambodia | 7258 (5986 to 8911) | 127.48 (105.13 to 156.52) | 632 (498 to 796) | 9.53 (7.51 to 12.01) | -8.30 (-8.7 to -7.89) |
| Cameroon | 3784 (2766 to 5139) | 63.74 (46.58 to 86.55) | 1976 (1314 to 2664) | 11.69 (7.77 to 15.76) | -5.03 (-5.84 to -4.21) |
| Canada | 49 (45 to 52) | 0.64 (0.59 to 0.68) | 7 (6 to 7) | 0.08 (0.07 to 0.09) | -6.05 (-6.28 to -5.82) |
| Central African Republic | 1679 (1181 to 2272) | 112.14 (78.89 to 151.78) | 801 (538 to 1124) | 27.67 (18.59 to 38.82) | -4.23 (-4.58 to -3.88) |
| Chad | 3785 (2821 to 4777) | 107.36 (80.02 to 135.52) | 6053 (4412 to 7698) | 55.28 (40.29 to 70.30) | -2.10 (-2.43 to -1.76) |
| Chile | 345 (324 to 367) | 6.54 (6.14 to 6.95) | 13 (11 to 14) | 0.26 (0.23 to 0.28) | -9.87 (-10.21 to -9.54) |
| China | 130093 (110122 to 152612) | 29.23 (24.74 to 34.29) | 4388 (3542 to 5374) | 1.31 (1.06 to 1.61) | -10.45 (-10.74 to -10.17) |
| Colombia | 1802 (1561 to 2062) | 11.97 (10.37 to 13.70) | 134 (100 to 180) | 0.92 (0.69 to 1.24) | -8.21 (-8.51 to -7.91) |
| Comoros | 183 (134 to 238) | 69.35 (50.86 to 90.02) | 45 (34 to 57) | 14.18 (10.81 to 18.19) | -5.17 (-5.40 to -4.93) |
| Congo | 459 (323 to 616) | 34.57 (24.32 to 46.43) | 99 (69 to 130) | 3.97 (2.77 to 5.22) | -7.14 (-7.99 to -6.29) |
| Cook Islands | 1 (1 to 2) | 14.57 (11.65 to 17.86) | 0 (0 to 0) | 3.08 (2.21 to 3.68) | -7.83 (-8.74 to -6.91) |
| Costa Rica | 64 (57 to 72) | 4.54 (4.05 to 5.10) | 6 (5 to 8) | 0.47 (0.40 to 0.56) | -7.48 (-7.89 to -7.07) |
| Croatia | 25 (22 to 27) | 1.87 (1.68 to 2.05) | 4 (3 to 5) | 0.49 (0.39 to 0.60) | -4.49 (-4.87 to -4.10) |
| Cuba | 118 (111 to 126) | 3.24 (3.04 to 3.45) | 24 (20 to 27) | 0.98 (0.82 to 1.14) | -3.59 (-4.10 to -3.09) |
| Cyprus | 2 (2 to 3) | 0.88 (0.68 to 1.20) | 0 (0 to 0) | 0.04 (0.03 to 0.05) | -9.34 (-9.69 to -9.00) |
| Czechia | 80 (74 to 85) | 2.63 (2.44 to 2.81) | 14 (11 to 16) | 0.62 (0.52 to 0.72) | -4.49 (-4.88 to -4.10) |
| Côte d'Ivoire | 4692 (3411 to 6155) | 67.82 (49.31 to 88.97) | 2116 (1429 to 3022) | 14.71 (9.94 to 21.01) | -4.22 (-4.89 to -3.55) |
| Democratic People's Republic of Korea | 1315 (982 to 1715) | 16.88 (12.60 to 22.01) | 159 (104 to 220) | 2.41 (1.57 to 3.33) | -6.02 (-6.36 to -5.68) |
| Democratic Republic of the Congo | 15968 (10919 to 21954) | 73.75 (50.43 to 101.40) | 3296 (2177 to 4748) | 6.88 (4.54 to 9.91) | -7.09 (-7.86 to -6.30) |
| Denmark | 12 (11 to 13) | 0.96 (0.89 to 1.04) | 1 (1 to 1) | 0.09 (0.08 to 0.10) | -8.41 (-8.86 to -7.95) |
| Djibouti | 90 (65 to 117) | 40.09 (29.12 to 52.11) | 36 (24 to 52) | 6.88 (4.62 to 9.93) | -5.54 (-6.40 to -4.67) |
| Dominica | 1 (1 to 1) | 3.66 (2.85 to 4.50) | 1 (0 to 1) | 3.03 (2.21 to 4.14) | -0.33 (-0.71 to 0.05) |
| Dominican Republic | 621 (501 to 735) | 17.72 (14.30 to 20.98) | 70 (47 to 94) | 1.79 (1.22 to 2.41) | -6.88 (-7.10 to -6.66) |
| Ecuador | 834 (761 to 908) | 16.83 (15.35 to 18.32) | 115 (87 to 153) | 1.73 (1.31 to 2.31) | -7.52 (-8.45 to -6.58) |
| Egypt | 14671 (12118 to 17783) | 52.54 (43.40 to 63.69) | 2493 (1934 to 3182) | 5.36 (4.15 to 6.84) | -5.58 (-6.11 to -5.05) |
| El Salvador | 500 (410 to 604) | 18.16 (14.89 to 21.91) | 28 (20 to 38) | 1.18 (0.85 to 1.60) | -9.22 (-9.70 to -8.73) |
| Equatorial Guinea | 195 (136 to 260) | 81.22 (56.61 to 108.29) | 45 (24 to 74) | 5.89 (3.17 to 9.63) | -9.29 (-9.67 to -8.91) |
| Eritrea | 1488 (1108 to 1881) | 75.64 (56.33 to 95.65) | 375 (266 to 515) | 11.67 (8.27 to 16.00) | -5.43 (-5.96 to -4.90) |
| Estonia | 16 (14 to 17) | 3.40 (3.10 to 3.71) | 1 (1 to 1) | 0.41 (0.35 to 0.47) | -6.21 (-6.81 to -5.59) |
| Eswatini | 176 (143 to 215) | 36.94 (29.94 to 45.06) | 60 (44 to 79) | 11.14 (8.18 to 14.76) | -3.26 (-4.32 to -2.18) |
| Ethiopia | 32902 (24138 to 42767) | 111.66 (81.91 to 145.13) | 5575 (4158 to 7058) | 9.75 (7.27 to 12.34) | -8.20 (-8.60 to -7.80) |
| Fiji | 41 (32 to 51) | 11.31 (8.88 to 14.19) | 13 (10 to 18) | 3.84 (2.83 to 5.15) | -3.49 (-4.27 to -2.71) |
| Finland | 8 (8 to 9) | 0.66 (0.60 to 0.71) | 1 (1 to 1) | 0.10 (0.09 to 0.12) | -6.22 (-6.56 to -5.89) |
| France | 95 (90 to 101) | 0.59 (0.56 to 0.63) | 11 (9 to 12) | 0.07 (0.06 to 0.08) | -7.42 (-7.73 to -7.11) |
| Gabon | 133 (94 to 179) | 26.25 (18.54 to 35.15) | 41 (25 to 61) | 4.92 (3.03 to 7.34) | -4.81 (-5.31 to -4.31) |
| Gambia | 321 (245 to 421) | 57.19 (43.60 to 74.86) | 68 (51 to 86) | 5.39 (4.02 to 6.83) | -7.89 (-8.44 to -7.33) |
| Georgia | 494 (429 to 562) | 27.48 (23.86 to 31.29) | 13 (10 to 16) | 1.35 (1.08 to 1.71) | -9.27 (-9.81 to -8.72) |
| Germany | 136 (128 to 143) | 0.78 (0.74 to 0.83) | 11 (10 to 12) | 0.07 (0.06 to 0.08) | -6.46 (-6.80 to -6.11) |
| Ghana | 3230 (2377 to 4256) | 39.11 (28.78 to 51.53) | 802 (524 to 1097) | 4.91 (3.21 to 6.73) | -5.94 (-6.51 to -5.37) |
| Greece | 13 (12 to 14) | 0.46 (0.44 to 0.49) | 2 (2 to 2) | 0.11 (0.10 to 0.13) | -4.44 (-4.95 to -3.93) |
| Greenland | 1 (1 to 2) | 7.63 (6.06 to 9.76) | 0 (0 to 0) | 0.49 (0.38 to 0.63) | -8.89 (-9.21 to -8.58) |
| Grenada | 3 (3 to 4) | 7.70 (6.33 to 9.25) | 1 (0 to 1) | 1.84 (1.48 to 2.26) | -3.99 (-4.30 to -3.69) |
| Guam | 3 (3 to 4) | 5.65 (4.78 to 6.56) | 2 (1 to 2) | 3.72 (2.82 to 4.74) | -0.59 (-0.98 to -0.21) |
| Guatemala | 2701 (2455 to 3011) | 54.99 (49.99 to 61.31) | 525 (410 to 674) | 7.94 (6.20 to 10.19) | -6.12 (-6.66 to -5.58) |
| Guinea | 5319 (3913 to 7100) | 161.82 (119.02 to 216.00) | 2299 (1514 to 3216) | 30.79 (20.28 to 43.08) | -4.60 (-4.84 to -4.37) |
| Guinea-Bissau | 562 (393 to 738) | 95.51 (66.87 to 125.42) | 102 (72 to 141) | 9.08 (6.42 to 12.61) | -6.94 (-7.68 to -6.20) |
| Guyana | 40 (33 to 49) | 10.57 (8.62 to 12.90) | 6 (4 to 8) | 2.01 (1.48 to 2.68) | -5.19 (-5.81 to -4.55) |
| Haiti | 2591 (2093 to 3216) | 77.42 (62.54 to 96.12) | 1151 (814 to 1525) | 20.53 (14.51 to 27.19) | -3.82 (-4.03 to -3.60) |
| Honduras | 583 (490 to 691) | 21.48 (18.06 to 25.46) | 53 (35 to 73) | 1.22 (0.80 to 1.68) | -9.21 (-9.64 to -8.78) |
| Hungary | 58 (53 to 63) | 2.01 (1.82 to 2.18) | 4 (3 to 5) | 0.20 (0.17 to 0.25) | -7.29 (-7.55 to -7.02) |
| Iceland | 1 (1 to 1) | 0.85 (0.76 to 0.95) | 0 (0 to 0) | 0.12 (0.10 to 0.14) | -6.80 (-7.10 to -6.49) |
| India | 166270 (133215 to 199995) | 40.51 (32.46 to 48.73) | 39365 (30988 to 48955) | 7.86 (6.19 to 9.78) | -4.65 (-4.97 to -4.32) |
| Indonesia | 22569 (19016 to 27191) | 25.80 (21.74 to 31.09) | 4309 (3331 to 5540) | 4.78 (3.69 to 6.14) | -5.22 (-5.41 to -5.02) |
| Iran (Islamic Republic of) | 4261 (3505 to 5830) | 13.52 (11.12 to 18.50) | 172 (141 to 205) | 0.66 (0.55 to 0.79) | -6.66 (-7.67 to -5.64) |
| Iraq | 1954 (1526 to 2560) | 18.97 (14.82 to 24.85) | 422 (317 to 550) | 2.40 (1.80 to 3.12) | -6.17 (-6.65 to -5.68) |
| Ireland | 14 (13 to 15) | 1.05 (0.97 to 1.13) | 1 (1 to 1) | 0.09 (0.08 to 0.11) | -7.80 (-8.57 to -7.02) |
| Israel | 18 (17 to 20) | 0.90 (0.83 to 0.97) | 2 (2 to 2) | 0.06 (0.05 to 0.06) | -8.17 (-8.58 to -7.75) |
| Italy | 96 (93 to 100) | 0.71 (0.68 to 0.74) | 6 (5 to 6) | 0.05 (0.05 to 0.06) | -8.45 (-8.76 to -8.15) |
| Jamaica | 38 (33 to 44) | 3.45 (3.04 to 4.00) | 5 (4 to 7) | 0.63 (0.46 to 0.84) | -5.46 (-5.80 to -5.11) |
| Japan | 251 (243 to 260) | 0.75 (0.73 to 0.78) | 23 (22 to 25) | 0.11 (0.10 to 0.12) | -6.14 (-7.00 to -5.27) |
| Jordan | 220 (184 to 261) | 10.48 (8.80 to 12.47) | 72 (59 to 90) | 1.47 (1.19 to 1.82) | -6.35 (-6.60 to -6.10) |
| Kazakhstan | 1786 (1615 to 1983) | 26.89 (24.30 to 29.85) | 84 (70 to 103) | 1.26 (1.03 to 1.53) | -9.94 (-10.94 to -8.92) |
| Kenya | 5623 (4547 to 7711) | 40.77 (32.97 to 55.91) | 969 (788 to 1196) | 3.94 (3.20 to 4.86) | -6.89 (-7.60 to -6.19) |
| Kiribati | 14 (11 to 18) | 39.40 (29.84 to 49.41) | 4 (3 to 6) | 8.23 (5.96 to 10.93) | -5.11 (-5.50 to -4.72) |
| Kuwait | 33 (29 to 37) | 4.77 (4.24 to 5.36) | 8 (7 to 10) | 0.76 (0.62 to 0.90) | -5.36 (-5.98 to -4.74) |
| Kyrgyzstan | 1072 (950 to 1214) | 50.77 (44.98 to 57.50) | 98 (83 to 114) | 3.48 (2.95 to 4.04) | -8.11 (-8.78 to -7.43) |
| Lao People's Democratic Republic | 3281 (2521 to 4336) | 144.62 (111.13 to 191.12) | 391 (276 to 533) | 13.07 (9.24 to 17.81) | -7.73 (-8.10 to -7.35) |
| Latvia | 22 (21 to 24) | 2.95 (2.76 to 3.15) | 1 (1 to 1) | 0.24 (0.20 to 0.28) | -7.46 (-8.27 to -6.65) |
| Lebanon | 145 (107 to 198) | 10.84 (7.96 to 14.81) | 19 (14 to 26) | 1.15 (0.86 to 1.57) | -7.21 (-7.67 to -6.76) |
| Lesotho | 278 (230 to 338) | 33.25 (27.53 to 40.42) | 141 (105 to 183) | 16.82 (12.55 to 21.76) | -1.73 (-2.43 to -1.03) |
| Liberia | 1975 (1399 to 2663) | 144.59 (102.41 to 194.94) | 218 (136 to 336) | 7.81 (4.86 to 12.05) | -8.91 (-9.32 to -8.49) |
| Libya | 155 (115 to 204) | 6.72 (4.98 to 8.83) | 25 (19 to 33) | 1.22 (0.91 to 1.59) | -3.97 (-4.36 to -3.57) |
| Lithuania | 25 (22 to 27) | 2.22 (2.02 to 2.42) | 1 (1 to 2) | 0.27 (0.24 to 0.31) | -6.05 (-6.97 to -5.13) |
| Luxembourg | 1 (1 to 1) | 0.71 (0.63 to 0.80) | 0 (0 to 0) | 0.05 (0.04 to 0.06) | -9.62 (-10.25 to -8.99) |
| Madagascar | 4681 (3959 to 5410) | 69.60 (58.86 to 80.44) | 1413 (1032 to 1852) | 9.45 (6.91 to 12.40) | -6.10 (-6.72 to -5.49) |
| Malawi | 6482 (4832 to 8410) | 116.31 (86.69 to 150.91) | 819 (548 to 1088) | 7.74 (5.17 to 10.27) | -8.92 (-9.67 to -8.17) |
| Malaysia | 527 (409 to 643) | 6.31 (4.90 to 7.71) | 136 (115 to 164) | 1.32 (1.12 to 1.60) | -4.41 (-4.99 to -3.83) |
| Maldives | 26 (21 to 32) | 19.99 (16.22 to 25.15) | 2 (2 to 3) | 1.53 (1.16 to 1.94) | -7.19 (-7.81 to -6.57) |
| Mali | 3380 (2401 to 4562) | 68.34 (48.54 to 92.24) | 1889 (1347 to 2563) | 13.27 (9.47 to 18.01) | -5.14 (-6.03 to -4.23) |
| Malta | 1 (1 to 1) | 0.85 (0.74 to 0.95) | 0 (0 to 0) | 0.29 (0.23 to 0.35) | -3.61 (-4.01 to -3.21) |
| Marshall Islands | 5 (4 to 6) | 18.24 (14.17 to 22.53) | 2 (1 to 2) | 7.86 (5.46 to 10.35) | -3.39 (-4.21 to -2.56) |
| Mauritania | 467 (353 to 581) | 41.22 (31.15 to 51.24) | 140 (106 to 178) | 6.00 (4.54 to 7.62) | -5.87 (-6.70 to -5.04) |
| Mauritius | 21 (19 to 23) | 4.81 (4.46 to 5.23) | 4 (4 to 5) | 1.47 (1.27 to 1.66) | -3.05 (-3.28 to -2.82) |
| Mexico | 8439 (7671 to 9402) | 19.46 (17.69 to 21.68) | 487 (362 to 655) | 1.13 (0.84 to 1.52) | -9.23 (-9.44 to -9.01) |
| Micronesia (Federated States of) | 16 (12 to 20) | 27.75 (21.60 to 34.19) | 2 (1 to 3) | 4.75 (3.35 to 6.13) | -6.34 (-6.56 to -6.13) |
| Monaco | 0 (0 to 0) | 0.77 (0.57 to 1.03) | 0 (0 to 0) | 0.17 (0.14 to 0.20) | -6.80 (-7.41 to -6.19) |
| Mongolia | 1224 (1039 to 1421) | 107.80 (91.56 to 125.22) | 78 (58 to 103) | 5.94 (4.41 to 7.83) | -8.88 (-9.16 to -8.59) |
| Montenegro | 5 (4 to 7) | 2.48 (1.86 to 3.27) | 0 (0 to 1) | 0.27 (0.20 to 0.36) | -6.87 (-7.52 to -6.20) |
| Morocco | 3390 (2642 to 4230) | 27.17 (21.17 to 33.89) | 135 (85 to 190) | 1.04 (0.66 to 1.47) | -9.80 (-10.60 to -8.98) |
| Mozambique | 6641 (4736 to 9672) | 87.72 (62.55 to 127.75) | 1334 (977 to 1827) | 7.52 (5.50 to 10.29) | -7.29 (-8.04 to -6.53) |
| Myanmar | 17146 (12416 to 22319) | 89.72 (64.97 to 116.79) | 2230 (1663 to 2866) | 10.74 (8.01 to 13.80) | -6.72 (-7.33 to -6.11) |
| Namibia | 191 (146 to 240) | 25.01 (19.21 to 31.54) | 67 (46 to 96) | 6.26 (4.25 to 8.92) | -3.74 (-4.44 to -3.04) |
| Nauru | 1 (1 to 2) | 28.25 (22.18 to 34.89) | 1 (1 to 1) | 16.29 (11.68 to 21.84) | -2.32 (-3.47 to -1.15) |
| Nepal | 9498 (7490 to 11267) | 91.78 (72.38 to 108.87) | 607 (448 to 817) | 4.88 (3.60 to 6.56) | -9.00 (-9.44 to -8.56) |
| Netherlands | 31 (29 to 33) | 0.80 (0.75 to 0.86) | 3 (3 to 4) | 0.09 (0.08 to 0.10) | -8.07 (-8.64 to -7.49) |
| New Zealand | 16 (15 to 17) | 1.45 (1.33 to 1.57) | 1 (1 to 2) | 0.11 (0.10 to 0.13) | -8.10 (-8.46 to -7.73) |
| Nicaragua | 621 (526 to 723) | 27.62 (23.39 to 32.15) | 31 (22 to 43) | 1.19 (0.85 to 1.64) | -9.54 (-9.96 to -9.12) |
| Niger | 11533 (8317 to 14866) | 237.72 (171.43 to 306.42) | 3902 (2518 to 5705) | 25.03 (16.16 to 36.60) | -7.41 (-7.85 to -6.97) |
| Nigeria | 62023 (47339 to 77567) | 127.83 (97.57 to 159.87) | 36756 (22614 to 52193) | 28.77 (17.70 to 40.85) | -4.22 (-4.71 to -3.73) |
| Niue | 0 (0 to 0) | 13.14 (10.49 to 16.43) | 0 (0 to 0) | 14.91 (13.29 to 16.74) | -3.42 (-4.60 to -2.23) |
| North Macedonia | 74 (59 to 92) | 10.57 (8.44 to 13.24) | 2 (1 to 2) | 0.43 (0.32 to 0.55) | -8.76 (-9.50 to -8.02) |
| Northern Mariana Islands | 1 (0 to 1) | 3.97 (3.01 to 5.14) | 0 (0 to 0) | 1.63 (1.30 to 2.02) | -2.81 (-3.19 to -2.43) |
| Norway | 8 (7 to 8) | 0.70 (0.66 to 0.75) | 1 (1 to 1) | 0.05 (0.04 to 0.05) | -9.19 (-9.60 to -8.77) |
| Oman | 110 (81 to 163) | 11.02 (8.11 to 16.24) | 10 (8 to 12) | 0.65 (0.53 to 0.80) | -7.34 (-8.10 to -6.57) |
| Pakistan | 27405 (22517 to 32519) | 44.95 (36.94 to 53.34) | 10656 (8037 to 13864) | 9.76 (7.36 to 12.69) | -3.83 (-4.39 to -3.28) |
| Palau | 1 (1 to 1) | 14.98 (11.07 to 19.45) | 0 (0 to 0) | 3.66 (2.90 to 4.54) | -5.05 (-5.43 to -4.67) |
| Palestine | 160 (121 to 210) | 13.44 (10.14 to 17.62) | 22 (17 to 28) | 0.92 (0.72 to 1.18) | -8.09 (-8.78 to -7.38) |
| Panama | 73 (63 to 85) | 6.75 (5.82 to 7.83) | 24 (19 to 32) | 1.60 (1.26 to 2.09) | -4.24 (-4.87 to -3.61) |
| Papua New Guinea | 1488 (1157 to 1900) | 69.91 (54.34 to 89.24) | 1466 (1103 to 1903) | 29.92 (22.51 to 38.84) | -2.49 (-2.93 to -2.04) |
| Paraguay | 211 (166 to 259) | 10.20 (8.03 to 12.53) | 32 (22 to 44) | 1.19 (0.81 to 1.66) | -6.81 (-7.48 to -6.13) |
| Peru | 4544 (3873 to 5277) | 42.67 (36.36 to 49.55) | 273 (189 to 372) | 2.19 (1.51 to 2.97) | -9.04 (-9.28 to -8.81) |
| Philippines | 11463 (9660 to 13950) | 35.76 (30.14 to 43.52) | 2563 (2070 to 3161) | 5.72 (4.62 to 7.06) | -4.92 (-5.29 to -4.54) |
| Poland | 288 (270 to 307) | 2.31 (2.17 to 2.47) | 34 (30 to 38) | 0.44 (0.38 to 0.49) | -4.03 (-4.48 to -3.58) |
| Portugal | 59 (55 to 62) | 1.98 (1.85 to 2.09) | 3 (3 to 4) | 0.18 (0.16 to 0.20) | -8.22 (-8.67 to -7.77) |
| Puerto Rico | 22 (20 to 24) | 1.67 (1.52 to 1.83) | 1 (1 to 1) | 0.15 (0.13 to 0.18) | -8.31 (-8.94 to -7.67) |
| Qatar | 6 (5 to 8) | 4.21 (3.15 to 5.47) | 2 (1 to 2) | 0.26 (0.20 to 0.33) | -8.52 (-8.80 to -8.24) |
| Republic of Korea | 344 (290 to 416) | 2.16 (1.82 to 2.61) | 7 (6 to 9) | 0.09 (0.07 to 0.11) | -9.65 (-9.92 to -9.39) |
| Republic of Moldova | 205 (181 to 234) | 12.98 (11.45 to 14.77) | 10 (8 to 13) | 1.47 (1.14 to 1.87) | -5.94 (-6.36 to -5.51) |
| Romania | 1735 (1621 to 1838) | 23.12 (21.60 to 24.49) | 79 (70 to 90) | 1.96 (1.72 to 2.22) | -6.84 (-7.32 to -6.35) |
| Russian Federation | 2586 (2494 to 2681) | 5.72 (5.52 to 5.93) | 241 (222 to 259) | 0.71 (0.66 to 0.77) | -5.66 (-6.49 to -4.82) |
| Rwanda | 4582 (3213 to 5962) | 110.72 (77.63 to 144.06) | 333 (247 to 427) | 5.18 (3.83 to 6.63) | -11.03 (-12.03 to -10.01) |
| Saint Kitts and Nevis | 1 (1 to 1) | 5.61 (4.99 to 6.35) | 0 (0 to 0) | 2.10 (1.63 to 2.69) | -3.45 (-3.75 to -3.14) |
| Saint Lucia | 3 (2 to 3) | 4.06 (3.32 to 4.82) | 1 (0 to 1) | 1.24 (0.90 to 1.69) | -4.03 (-4.19 to -3.87) |
| Saint Vincent and the Grenadines | 3 (2 to 3) | 4.88 (3.97 to 5.92) | 1 (0 to 1) | 1.51 (1.19 to 1.94) | -3.91 (-4.21 to -3.62) |
| Samoa | 16 (12 to 22) | 17.95 (12.87 to 23.78) | 7 (4 to 10) | 6.73 (4.40 to 9.66) | -3.34 (-3.48 to -3.20) |
| San Marino | 0 (0 to 0) | 0.60 (0.46 to 0.80) | 0 (0 to 0) | 0.06 (0.04 to 0.08) | -7.31 (-7.56 to -7.07) |
| Sao Tome and Principe | 39 (30 to 47) | 55.37 (42.49 to 67.55) | 2 (2 to 3) | 2.45 (1.71 to 3.29) | -9.83 (-10.49 to -9.17) |
| Saudi Arabia | 928 (698 to 1298) | 11.31 (8.50 to 15.81) | 31 (23 to 40) | 0.30 (0.23 to 0.39) | -11.08 (-11.43 to -10.72) |
| Senegal | 2907 (2237 to 3622) | 65.36 (50.30 to 81.45) | 444 (345 to 548) | 5.48 (4.25 to 6.75) | -7.41 (-8.23 to -6.59) |
| Serbia | 137 (107 to 185) | 4.71 (3.70 to 6.38) | 4 (3 to 6) | 0.24 (0.18 to 0.30) | -9.34 (-9.85 to -8.83) |
| Seychelles | 2 (2 to 2) | 6.19 (5.28 to 7.17) | 1 (1 to 1) | 3.25 (2.60 to 4.10) | -0.48 (-0.89 to -0.07) |
| Sierra Leone | 3239 (2270 to 4352) | 146.86 (102.93 to 197.29) | 562 (393 to 794) | 12.35 (8.64 to 17.44) | -8.16 (-8.82 to -7.50) |
| Singapore | 33 (30 to 35) | 3.54 (3.29 to 3.79) | 4 (3 to 4) | 0.36 (0.32 to 0.41) | -6.90 (-7.29 to -6.51) |
| Slovakia | 103 (93 to 113) | 5.86 (5.28 to 6.44) | 8 (7 to 10) | 0.75 (0.61 to 0.91) | -6.58 (-6.98 to -6.17) |
| Slovenia | 15 (13 to 16) | 2.61 (2.36 to 2.84) | 2 (1 to 2) | 0.41 (0.35 to 0.46) | -5.81 (-6.03 to -5.58) |
| Solomon Islands | 72 (53 to 95) | 37.20 (27.21 to 48.82) | 31 (24 to 39) | 9.24 (7.17 to 11.79) | -4.71 (-5.31 to -4.10) |
| Somalia | 4300 (2855 to 5907) | 90.86 (60.32 to 124.83) | 3966 (2590 to 5577) | 31.02 (20.26 to 43.62) | -2.83 (-3.23 to -2.43) |
| South Africa | 6281 (5345 to 7588) | 35.65 (30.33 to 43.06) | 1293 (1031 to 1534) | 6.49 (5.18 to 7.70) | -5.11 (-5.85 to -4.36) |
| South Sudan | 3183 (2393 to 4228) | 96.85 (72.81 to 128.67) | 2349 (1625 to 3544) | 42.81 (29.62 to 64.59) | -2.23 (-2.67 to -1.79) |
| Spain | 78 (74 to 83) | 0.70 (0.67 to 0.74) | 7 (7 to 8) | 0.08 (0.08 to 0.09) | -6.37 (-6.81 to -5.92) |
| Sri Lanka | 331 (289 to 372) | 4.58 (3.99 to 5.13) | 66 (48 to 89) | 0.96 (0.70 to 1.29) | -4.60 (-5.13 to -4.07) |
| Sudan | 7995 (5937 to 11762) | 72.38 (53.75 to 106.48) | 802 (588 to 1105) | 3.75 (2.75 to 5.17) | -9.00 (-9.71 to -8.29) |
| Suriname | 17 (13 to 21) | 10.11 (7.67 to 12.50) | 5 (4 to 8) | 2.90 (2.05 to 4.05) | -4.08 (-4.28 to -3.89) |
| Sweden | 12 (11 to 13) | 0.56 (0.52 to 0.60) | 1 (1 to 1) | 0.05 (0.04 to 0.05) | -7.50 (-7.90 to -7.09) |
| Switzerland | 15 (14 to 16) | 0.94 (0.87 to 1.03) | 1 (1 to 2) | 0.08 (0.07 to 0.10) | -7.77 (-7.91 to -7.62) |
| Syrian Arab Republic | 1150 (859 to 1526) | 15.65 (11.68 to 20.75) | 116 (88 to 147) | 2.13 (1.61 to 2.70) | -4.43 (-5.24 to -3.61) |
| Taiwan (Province of China) | 206 (195 to 218) | 2.81 (2.66 to 2.98) | 10 (9 to 11) | 0.24 (0.22 to 0.27) | -8.14 (-8.51 to -7.76) |
| Tajikistan | 2515 (2148 to 2884) | 87.49 (74.74 to 100.34) | 962 (673 to 1280) | 21.60 (15.11 to 28.72) | -4.52 (-4.86 to -4.17) |
| Thailand | 1512 (1182 to 1910) | 6.65 (5.20 to 8.40) | 292 (247 to 341) | 2.14 (1.81 to 2.50) | -3.49 (-3.71 to -3.26) |
| Timor-Leste | 540 (444 to 675) | 132.83 (109.06 to 166.07) | 121 (91 to 154) | 17.66 (13.32 to 22.46) | -7.02 (-7.42 to -6.61) |
| Togo | 1223 (922 to 1564) | 56.82 (42.82 to 72.64) | 365 (244 to 500) | 8.73 (5.85 to 11.97) | -5.49 (-6.27 to -4.70) |
| Tokelau | 0 (0 to 0) | 16.71 (12.49 to 21.33) | 0 (0 to 0) | 27.67 (20.07 to 33.58) | -2.89 (-4.56 to -1.19) |
| Tonga | 8 (6 to 10) | 15.51 (12.05 to 19.03) | 3 (2 to 4) | 6.12 (4.57 to 8.13) | -3.06 (-3.49 to -2.62) |
| Trinidad and Tobago | 21 (18 to 25) | 4.12 (3.45 to 4.82) | 3 (2 to 4) | 0.79 (0.60 to 1.06) | -5.03 (-5.62 to -4.45) |
| Tunisia | 629 (433 to 1002) | 15.77 (10.86 to 25.11) | 44 (29 to 62) | 1.22 (0.81 to 1.74) | -7.07 (-7.47 to -6.68) |
| Turkmenistan | 1408 (1244 to 1607) | 74.70 (66.01 to 85.22) | 261 (215 to 316) | 13.31 (10.95 to 16.13) | -5.30 (-5.68 to -4.91) |
| Tuvalu | 3 (3 to 5) | 81.17 (61.82 to 107.21) | 0 (0 to 1) | 9.29 (6.94 to 11.96) | -6.87 (-7.19 to -6.54) |
| Türkiye | 7048 (5306 to 9665) | 26.41 (19.88 to 36.22) | 130 (105 to 160) | 0.53 (0.42 to 0.65) | -12.48 (-12.97 to -11.99) |
| Uganda | 5883 (3898 to 8709) | 57.13 (37.85 to 84.57) | 1941 (1267 to 2719) | 7.78 (5.08 to 10.90) | -6.44 (-7.00 to -5.87) |
| Ukraine | 502 (433 to 573) | 3.34 (2.88 to 3.81) | 68 (57 to 79) | 0.80 (0.68 to 0.93) | -3.80 (-4.18 to -3.42) |
| United Arab Emirates | 43 (33 to 56) | 6.14 (4.71 to 7.85) | 5 (4 to 7) | 0.32 (0.26 to 0.40) | -7.99 (-8.63 to -7.34) |
| United Kingdom | 158 (152 to 163) | 1.06 (1.02 to 1.10) | 17 (15 to 18) | 0.11 (0.10 to 0.12) | -7.92 (-8.45 to -7.38) |
| United Republic of Tanzania | 14726 (10937 to 18713) | 98.68 (73.29 to 125.40) | 2657 (1892 to 3509) | 8.61 (6.13 to 11.37) | -7.49 (-8.13 to -6.85) |
| United States Virgin Islands | 1 (1 to 1) | 2.09 (1.56 to 2.67) | 0 (0 to 0) | 0.21 (0.14 to 0.29) | -7.26 (-7.49 to -7.03) |
| United States of America | 669 (645 to 694) | 0.90 (0.87 to 0.94) | 94 (86 to 102) | 0.12 (0.11 to 0.13) | -6.94 (-7.31 to -6.58) |
| Uruguay | 40 (37 to 44) | 3.73 (3.41 to 4.07) | 4 (3 to 4) | 0.40 (0.33 to 0.47) | -7.90 (-8.45 to -7.34) |
| Uzbekistan | 6044 (5575 to 6599) | 56.62 (52.22 to 61.81) | 1048 (861 to 1273) | 8.31 (6.83 to 10.09) | -5.64 (-6.42 to -4.85) |
| Vanuatu | 20 (14 to 26) | 23.78 (17.31 to 30.71) | 15 (11 to 20) | 10.16 (7.32 to 13.49) | -2.58 (-3.20 to -1.96) |
| Venezuela (Bolivarian Republic of) | 862 (809 to 918) | 9.50 (8.91 to 10.11) | 250 (184 to 336) | 2.86 (2.11 to 3.84) | -2.72 (-3.29 to -2.15) |
| Viet Nam | 6003 (4650 to 7309) | 17.83 (13.81 to 21.71) | 877 (654 to 1135) | 2.77 (2.07 to 3.59) | -5.05 (-5.44 to -4.66) |
| Yemen | 4804 (3519 to 7106) | 57.13 (41.86 to 84.52) | 777 (558 to 1025) | 4.45 (3.19 to 5.86) | -8.04 (-8.52 to -7.55) |
| Zambia | 4752 (3714 to 6237) | 101.10 (79.01 to 132.68) | 753 (501 to 1007) | 7.20 (4.79 to 9.64) | -8.25 (-9.11 to -7.39) |
| Zimbabwe | 1916 (1623 to 2239) | 31.82 (26.95 to 37.18) | 1208 (928 to 1493) | 15.13 (11.62 to 18.70) | -2.00 (-2.71 to -1.27) |
| **DALYs** |  |  |  |  |  |
| Afghanistan | 508506 (380307 to 668833) | 9124.52 (6824.15 to 12001.39) | 178788 (130496 to 241059) | 1005.74 (734.09 to 1356.04) | -6.88 (-7.53 to -6.22) |
| Albania | 39900 (33342 to 46590) | 2759.51 (2305.98 to 3222.20) | 613 (436 to 798) | 99.39 (70.58 to 129.25) | -10.96 (-11.22 to -10.70) |
| Algeria | 163107 (120911 to 232282) | 1206.72 (894.54 to 1718.50) | 15195 (11894 to 19550) | 92.38 (72.31 to 118.86) | -6.54 (-7.21 to -5.87) |
| American Samoa | 227 (190 to 271) | 948.88 (792.03 to 1132.72) | 70 (51 to 91) | 366.67 (269.10 to 476.84) | -3.32 (-3.47 to -3.16) |
| Andorra | 8 (5 to 11) | 55.80 (38.23 to 80.24) | 0 (0 to 0) | 2.51 (1.83 to 3.22) | -9.81 (-10.35 to -9.26) |
| Angola | 547531 (414035 to 682334) | 9495.82 (7180.61 to 11833.70) | 160120 (109934 to 222487) | 857.37 (588.65 to 1191.32) | -7.57 (-8.25 to -6.89) |
| Antigua and Barbuda | 55 (46 to 66) | 229.89 (190.58 to 276.67) | 33 (29 to 38) | 142.57 (124.23 to 163.33) | -2.33 (-2.82 to -1.84) |
| Argentina | 58795 (54929 to 63376) | 451.34 (421.66 to 486.51) | 8273 (7143 to 9527) | 60.34 (52.10 to 69.48) | -5.93 (-6.44 to -5.41) |
| Armenia | 28290 (24985 to 32498) | 2130.58 (1881.62 to 2447.44) | 1473 (1177 to 1833) | 193.03 (154.19 to 240.23) | -6.50 (-7.05 to -5.95) |
| Australia | 3047 (2792 to 3296) | 58.91 (53.99 to 63.73) | 303 (256 to 359) | 4.86 (4.10 to 5.76) | -7.58 (-8.00 to -7.16) |
| Austria | 852 (785 to 920) | 45.47 (41.91 to 49.12) | 101 (88 to 114) | 5.75 (5.03 to 6.49) | -6.09 (-6.36 to -5.82) |
| Azerbaijan | 224711 (192860 to 257654) | 7179.04 (6161.47 to 8231.49) | 20003 (15372 to 25013) | 656.52 (504.54 to 820.96) | -7.07 (-7.52 to -6.61) |
| Bahamas | 469 (394 to 568) | 435.96 (365.52 to 527.63) | 79 (60 to 105) | 68.49 (52.35 to 90.78) | -6.14 (-6.67 to -5.61) |
| Bahrain | 1026 (861 to 1260) | 512.97 (430.50 to 629.60) | 112 (94 to 132) | 27.70 (23.19 to 32.81) | -8.30 (-8.65 to -7.95) |
| Bangladesh | 3379552 (2846981 to 3902397) | 5599.06 (4716.73 to 6465.28) | 226025 (174358 to 289235) | 370.60 (285.88 to 474.24) | -8.25 (-8.97 to -7.52) |
| Barbados | 201 (169 to 235) | 236.99 (199.03 to 276.60) | 32 (23 to 44) | 48.83 (35.39 to 66.90) | -5.61 (-6.37 to -4.84) |
| Belarus | 10550 (9037 to 12337) | 336.18 (287.99 to 393.16) | 910 (713 to 1175) | 44.91 (35.20 to 57.99) | -5.81 (-6.09 to -5.53) |
| Belgium | 1890 (1756 to 2033) | 76.48 (71.04 to 82.24) | 211 (182 to 244) | 8.28 (7.14 to 9.61) | -7.82 (-8.14 to -7.50) |
| Belize | 1225 (1095 to 1387) | 1194.66 (1067.30 to 1352.45) | 342 (279 to 413) | 202.25 (165.13 to 244.23) | -5.79 (-6.19 to -5.40) |
| Benin | 241848 (180457 to 319520) | 8427.64 (6288.37 to 11134.28) | 85240 (53401 to 127494) | 1134.07 (710.47 to 1696.24) | -6.13 (-6.68 to -5.58) |
| Bermuda | 22 (18 to 26) | 137.47 (115.26 to 161.51) | 5 (3 to 6) | 39.15 (28.97 to 49.01) | -4.10 (-4.36 to -3.84) |
| Bhutan | 13449 (6798 to 18466) | 3948.47 (1995.77 to 5421.51) | 1225 (833 to 1672) | 480.36 (326.60 to 655.85) | -6.92 (-7.23 to -6.60) |
| Bolivia (Plurinational State of) | 249244 (180575 to 320308) | 7442.66 (5392.15 to 9564.71) | 18482 (14023 to 23428) | 406.48 (308.42 to 515.26) | -8.89 (-9.30 to -8.49) |
| Bosnia and Herzegovina | 1953 (1482 to 2407) | 131.78 (100.00 to 162.44) | 222 (175 to 271) | 33.36 (26.37 to 40.69) | -4.51 (-5.05 to -3.96) |
| Botswana | 14142 (10870 to 17888) | 1909.93 (1468.00 to 2415.75) | 5943 (4192 to 8305) | 648.12 (457.10 to 905.63) | -3.04 (-3.73 to -2.35) |
| Brazil | 1069268 (959917 to 1202263) | 1591.35 (1428.60 to 1789.28) | 53636 (43540 to 64682) | 83.92 (68.12 to 101.20) | -9.11 (-9.53 to -8.68) |
| Brunei Darussalam | 230 (193 to 274) | 200.13 (167.44 to 237.89) | 72 (55 to 94) | 55.68 (42.74 to 72.83) | -3.81 (-3.96 to -3.65) |
| Bulgaria | 20096 (18774 to 21462) | 850.33 (794.38 to 908.13) | 1275 (1062 to 1479) | 98.49 (82.03 to 114.29) | -6.95 (-7.73 to -6.17) |
| Burkina Faso | 541460 (396788 to 687548) | 9571.14 (7013.83 to 12153.47) | 212344 (137101 to 291751) | 1658.18 (1070.62 to 2278.27) | -5.20 (-5.85 to -4.55) |
| Burundi | 215539 (144048 to 281825) | 6831.39 (4565.52 to 8932.29) | 36594 (22573 to 54041) | 503.04 (310.30 to 742.88) | -7.84 (-8.70 to -6.97) |
| Cabo Verde | 2673 (1996 to 3504) | 1374.36 (1026.58 to 1802.00) | 379 (287 to 486) | 196.68 (148.65 to 251.95) | -7.20 (-7.77 to -6.62) |
| Cambodia | 642901 (529808 to 790103) | 11292.01 (9305.62 to 13877.48) | 55359 (43487 to 69727) | 834.83 (655.80 to 1051.52) | -8.33 (-8.74 to -7.91) |
| Cameroon | 333166 (243842 to 451876) | 5611.63 (4107.11 to 7611.11) | 173029 (115230 to 232886) | 1023.67 (681.72 to 1377.80) | -5.04 (-5.86 to -4.22) |
| Canada | 4229 (3938 to 4498) | 55.18 (51.38 to 58.68) | 564 (497 to 635) | 6.85 (6.04 to 7.71) | -6.03 (-6.26 to -5.80) |
| Central African Republic | 148294 (104119 to 201054) | 9905.17 (6954.50 to 13429.20) | 70104 (47201 to 98509) | 2422.40 (1630.99 to 3403.88) | -4.26 (-4.61 to -3.90) |
| Chad | 333334 (249064 to 420362) | 9455.54 (7065.07 to 11924.22) | 532037 (387973 to 677186) | 4858.98 (3543.27 to 6184.59) | -2.10 (-2.44 to -1.77) |
| Chile | 30604 (28676 to 32505) | 580.23 (543.66 to 616.26) | 1064 (957 to 1164) | 21.74 (19.56 to 23.78) | -10.04 (-10.37 to -9.71) |
| China | 11524182 (9760846 to 13527903) | 2589.46 (2193.24 to 3039.69) | 387655 (313122 to 474773) | 115.96 (93.66 to 142.02) | -10.46 (-10.74 to -10.17) |
| Colombia | 159144 (137789 to 182129) | 1057.24 (915.37 to 1209.94) | 11557 (8551 to 15618) | 79.38 (58.73 to 107.27) | -8.30 (-8.61 to -7.99) |
| Comoros | 16200 (11924 to 21016) | 6128.91 (4511.42 to 7950.96) | 3884 (2950 to 4981) | 1237.11 (939.64 to 1586.56) | -5.20 (-5.44 to -4.97) |
| Congo | 40349 (28398 to 54279) | 3041.37 (2140.55 to 4091.32) | 8440 (5874 to 11163) | 339.41 (236.19 to 448.89) | -7.22 (-8.08 to -6.35) |
| Cook Islands | 110 (88 to 135) | 1277.01 (1020.02 to 1568.13) | 14 (10 to 17) | 266.62 (190.99 to 318.48) | -7.92 (-8.86 to -6.98) |
| Costa Rica | 5710 (5088 to 6415) | 402.62 (358.79 to 452.32) | 561 (473 to 669) | 40.98 (34.59 to 48.90) | -7.54 (-7.96 to -7.13) |
| Croatia | 2159 (1946 to 2370) | 162.76 (146.70 to 178.71) | 344 (269 to 422) | 42.71 (33.40 to 52.42) | -4.49 (-4.88 to -4.10) |
| Cuba | 10447 (9818 to 11145) | 286.06 (268.84 to 305.18) | 2086 (1745 to 2455) | 86.88 (72.66 to 102.25) | -3.57 (-4.07 to -3.07) |
| Cyprus | 201 (154 to 275) | 77.47 (59.33 to 106.01) | 9 (7 to 11) | 3.19 (2.51 to 4.02) | -9.41 (-9.72 to -9.10) |
| Czechia | 6955 (6441 to 7437) | 228.49 (211.62 to 244.32) | 1189 (986 to 1384) | 53.56 (44.40 to 62.33) | -4.49 (-4.89 to -4.09) |
| Côte d'Ivoire | 414494 (301586 to 544262) | 5991.10 (4359.12 to 7866.76) | 185319 (125177 to 264469) | 1288.31 (870.21 to 1838.55) | -4.24 (-4.92 to -3.57) |
| Democratic People's Republic of Korea | 116461 (87065 to 152042) | 1495.14 (1117.74 to 1951.92) | 13868 (8963 to 19146) | 210.05 (135.76 to 289.99) | -6.07 (-6.41 to -5.73) |
| Democratic Republic of the Congo | 1409464 (963003 to 1937809) | 6510.17 (4448.02 to 8950.54) | 284775 (187477 to 413888) | 594.43 (391.33 to 863.94) | -7.15 (-7.93 to -6.35) |
| Denmark | 1050 (969 to 1138) | 84.00 (77.51 to 91.02) | 103 (89 to 119) | 7.93 (6.84 to 9.16) | -8.40 (-8.85 to -7.95) |
| Djibouti | 7908 (5741 to 10269) | 3526.23 (2559.68 to 4578.86) | 3157 (2122 to 4562) | 597.47 (401.54 to 863.50) | -5.57 (-6.43 to -4.70) |
| Dominica | 106 (82 to 130) | 322.80 (251.13 to 396.28) | 52 (38 to 71) | 266.20 (194.31 to 363.52) | -0.34 (-0.72 to 0.05) |
| Dominican Republic | 55053 (44449 to 65231) | 1571.97 (1269.20 to 1862.61) | 6081 (4102 to 8230) | 156.30 (105.43 to 211.53) | -6.93 (-7.16 to -6.71) |
| Ecuador | 73559 (67064 to 80148) | 1483.78 (1352.76 to 1616.70) | 9955 (7489 to 13336) | 150.47 (113.20 to 201.57) | -7.58 (-8.52 to -6.63) |
| Egypt | 1293753 (1067215 to 1569361) | 4633.33 (3822.03 to 5620.37) | 218922 (169525 to 280164) | 470.29 (364.18 to 601.85) | -5.58 (-6.11 to -5.04) |
| El Salvador | 44289 (36221 to 53456) | 1607.35 (1314.56 to 1940.05) | 2431 (1735 to 3302) | 102.05 (72.82 to 138.63) | -9.31 (-9.80 to -8.82) |
| Equatorial Guinea | 17178 (11971 to 22868) | 7157.96 (4988.49 to 9528.96) | 3907 (2109 to 6389) | 507.34 (273.80 to 829.64) | -9.35 (-9.72 to -8.97) |
| Eritrea | 130303 (96853 to 164715) | 6625.16 (4924.44 to 8374.80) | 32395 (22999 to 44403) | 1007.11 (715.01 to 1380.43) | -5.47 (-6.00 to -4.93) |
| Estonia | 1381 (1258 to 1511) | 300.59 (273.82 to 328.94) | 100 (86 to 117) | 35.84 (30.76 to 41.65) | -6.21 (-6.81 to -5.60) |
| Eswatini | 15517 (12562 to 18971) | 3252.21 (2632.91 to 3976.16) | 5184 (3794 to 6886) | 968.98 (709.22 to 1287.11) | -3.30 (-4.37 to -2.21) |
| Ethiopia | 2894748 (2123926 to 3762912) | 9823.61 (7207.75 to 12769.82) | 486439 (362953 to 616676) | 850.45 (634.56 to 1078.14) | -8.22 (-8.62 to -7.82) |
| Fiji | 3534 (2771 to 4444) | 985.27 (772.47 to 1239.03) | 1166 (858 to 1562) | 332.41 (244.71 to 445.57) | -3.50 (-4.28 to -2.70) |
| Finland | 718 (659 to 780) | 56.57 (51.95 to 61.45) | 100 (89 to 113) | 8.74 (7.77 to 9.80) | -6.27 (-6.60 to -5.94) |
| France | 8261 (7765 to 8759) | 51.18 (48.11 to 54.26) | 925 (821 to 1036) | 5.87 (5.21 to 6.58) | -7.39 (-7.70 to -7.08) |
| Gabon | 11753 (8304 to 15737) | 2312.70 (1634.15 to 3096.65) | 3508 (2170 to 5245) | 424.19 (262.44 to 634.24) | -4.87 (-5.38 to -4.36) |
| Gambia | 28168 (21521 to 36880) | 5013.90 (3830.81 to 6564.76) | 5850 (4397 to 7397) | 461.85 (347.11 to 584.00) | -7.96 (-8.51 to -7.40) |
| Georgia | 43594 (37815 to 49664) | 2426.18 (2104.54 to 2763.99) | 1084 (865 to 1381) | 116.69 (93.08 to 148.60) | -9.32 (-9.86 to -8.78) |
| Germany | 11724 (11092 to 12372) | 67.65 (64.00 to 71.39) | 946 (867 to 1025) | 5.93 (5.44 to 6.43) | -6.49 (-6.84 to -6.14) |
| Ghana | 283047 (208134 to 373498) | 3426.88 (2519.91 to 4521.98) | 69010 (45062 to 94884) | 423.01 (276.22 to 581.61) | -6.00 (-6.57 to -5.42) |
| Greece | 1120 (1053 to 1191) | 39.86 (37.47 to 42.38) | 186 (158 to 216) | 9.75 (8.31 to 11.36) | -4.39 (-4.90 to -3.88) |
| Greenland | 121 (96 to 155) | 675.24 (535.49 to 864.15) | 7 (5 to 8) | 43.02 (33.59 to 54.89) | -8.92 (-9.24 to -8.60) |
| Grenada | 287 (235 to 344) | 679.94 (557.94 to 816.88) | 48 (39 to 59) | 160.93 (129.18 to 197.37) | -4.02 (-4.32 to -3.71) |
| Guam | 268 (227 to 311) | 495.12 (419.44 to 574.91) | 159 (121 to 203) | 327.85 (249.38 to 418.21) | -0.59 (-0.97 to -0.21) |
| Guatemala | 236996 (215257 to 264462) | 4826.05 (4383.36 to 5385.35) | 46166 (35997 to 59347) | 697.90 (544.18 to 897.17) | -6.12 (-6.67 to -5.57) |
| Guinea | 468752 (344748 to 625430) | 14259.99 (10487.65 to 19026.33) | 201833 (132831 to 282655) | 2703.93 (1779.52 to 3786.68) | -4.61 (-4.85 to -4.38) |
| Guinea-Bissau | 49200 (34484 to 64676) | 8365.95 (5863.68 to 10997.50) | 8734 (6156 to 12134) | 780.84 (550.39 to 1084.85) | -6.99 (-7.74 to -6.24) |
| Guyana | 3571 (2906 to 4358) | 935.79 (761.60 to 1142.16) | 491 (362 to 655) | 174.99 (129.01 to 233.47) | -5.25 (-5.88 to -4.62) |
| Haiti | 229429 (185264 to 284703) | 6855.93 (5536.18 to 8507.68) | 101881 (71853 to 135272) | 1816.80 (1281.31 to 2412.24) | -3.82 (-4.04 to -3.60) |
| Honduras | 51513 (43268 to 61134) | 1898.31 (1594.49 to 2252.87) | 4648 (3052 to 6418) | 106.83 (70.15 to 147.51) | -9.23 (-9.66 to -8.80) |
| Hungary | 5128 (4668 to 5571) | 176.68 (160.84 to 191.95) | 332 (272 to 402) | 17.69 (14.51 to 21.46) | -7.32 (-7.58 to -7.06) |
| Iceland | 62 (55 to 69) | 73.01 (64.80 to 81.10) | 9 (7 to 10) | 9.78 (8.29 to 11.64) | -6.84 (-7.14 to -6.54) |
| India | 14718899 (11783490 to 17704648) | 3586.33 (2871.11 to 4313.83) | 3489198 (2744611 to 4342836) | 697.04 (548.29 to 867.57) | -4.64 (-4.96 to -4.31) |
| Indonesia | 1998172 (1684393 to 2408521) | 2284.58 (1925.83 to 2753.75) | 380181 (293631 to 489554) | 421.40 (325.47 to 542.64) | -5.23 (-5.43 to -5.03) |
| Iran (Islamic Republic of) | 377173 (309806 to 517148) | 1197.01 (983.21 to 1641.24) | 14163 (11566 to 17033) | 54.78 (44.74 to 65.88) | -6.78 (-7.81 to -5.74) |
| Iraq | 172775 (134703 to 226527) | 1677.67 (1307.98 to 2199.61) | 36842 (27438 to 48044) | 208.89 (155.57 to 272.41) | -6.21 (-6.70 to -5.72) |
| Ireland | 1193 (1105 to 1283) | 89.79 (83.15 to 96.53) | 105 (93 to 119) | 7.97 (7.05 to 9.02) | -7.79 (-8.56 to -7.01) |
| Israel | 1583 (1453 to 1715) | 78.90 (72.42 to 85.47) | 163 (142 to 185) | 4.82 (4.21 to 5.5) | -8.22 (-8.63 to -7.80) |
| Italy | 8378 (8041 to 8742) | 61.52 (59.04 to 64.18) | 483 (418 to 543) | 4.60 (3.99 to 5.18) | -8.48 (-8.78 to -8.18) |
| Jamaica | 3343 (2953 to 3861) | 305.98 (270.27 to 353.44) | 461 (340 to 609) | 56.48 (41.73 to 74.69) | -5.41 (-5.75 to -5.07) |
| Japan | 21596 (20871 to 22369) | 64.73 (62.56 to 67.05) | 2043 (1907 to 2189) | 9.62 (8.98 to 10.31) | -6.10 (-6.96 to -5.23) |
| Jordan | 19381 (16264 to 23114) | 924.41 (775.73 to 1102.45) | 6307 (5097 to 7803) | 128.05 (103.5 to 158.44) | -6.38 (-6.63 to -6.13) |
| Kazakhstan | 158364 (143138 to 175951) | 2383.88 (2154.69 to 2648.63) | 7405 (6097 to 9027) | 110.13 (90.68 to 134.25) | -9.97 (-10.98 to -8.96) |
| Kenya | 496664 (401245 to 681202) | 3601.15 (2909.29 to 4939.17) | 83436 (67575 to 102869) | 339.16 (274.69 to 418.16) | -6.98 (-7.69 to -6.26) |
| Kiribati | 1276 (965 to 1602) | 3481.54 (2632.85 to 4372.03) | 381 (275 to 509) | 713.77 (513.74 to 952.12) | -5.17 (-5.56 to -4.77) |
| Kuwait | 2849 (2529 to 3208) | 415.36 (368.70 to 467.65) | 714 (584 to 847) | 65.16 (53.32 to 77.33) | -5.42 (-6.04 to -4.79) |
| Kyrgyzstan | 95001 (84192 to 107655) | 4499.45 (3987.50 to 5098.80) | 8610 (7306 to 9995) | 305.01 (258.82 to 354.06) | -8.13 (-8.81 to -7.44) |
| Lao People's Democratic Republic | 290476 (223007 to 383933) | 12804.86 (9830.66 to 16924.66) | 34499 (24352 to 47129) | 1153.24 (814.03 to 1575.41) | -7.74 (-8.12 to -7.35) |
| Latvia | 1964 (1837 to 2096) | 261.07 (244.10 to 278.57) | 83 (70 to 96) | 21.40 (18.13 to 24.82) | -7.47 (-8.27 to -6.67) |
| Lebanon | 12769 (9354 to 17469) | 953.53 (698.48 to 1304.46) | 1647 (1233 to 2269) | 98.86 (73.97 to 136.19) | -7.28 (-7.74 to -6.82) |
| Lesotho | 24591 (20266 to 29897) | 2936.90 (2420.31 to 3570.50) | 12288 (9103 to 15932) | 1464.94 (1085.18 to 1899.29) | -1.80 (-2.5 to -1.09) |
| Liberia | 174385 (123584 to 235181) | 12764.53 (9046.08 to 17214.69) | 18817 (11670 to 29040) | 674.45 (418.26 to 1040.84) | -8.96 (-9.38 to -8.54) |
| Libya | 13666 (10126 to 18028) | 592.05 (438.70 to 781.02) | 2153 (1597 to 2839) | 103.16 (76.49 to 135.97) | -4.06 (-4.46 to -3.66) |
| Lithuania | 2181 (1988 to 2377) | 196.95 (179.47 to 214.64) | 130 (113 to 149) | 24.30 (21.16 to 27.79) | -6.08 (-6.99 to -5.17) |
| Luxembourg | 54 (48 to 60) | 61.07 (54.69 to 68.51) | 6 (5 to 7) | 4.28 (3.60 to 5.18) | -9.57 (-10.20 to -8.93) |
| Madagascar | 411623 (347670 to 475851) | 6119.89 (5169.06 to 7074.82) | 121663 (88410 to 159937) | 814.14 (591.62 to 1070.26) | -6.16 (-6.79 to -5.54) |
| Malawi | 571848 (425953 to 742260) | 10260.51 (7642.75 to 13318.18) | 70943 (47222 to 93907) | 669.85 (445.87 to 886.68) | -8.98 (-9.73 to -8.22) |
| Malaysia | 45787 (35408 to 56159) | 548.81 (424.40 to 673.13) | 11506 (9651 to 13957) | 112.20 (94.11 to 136.10) | -4.48 (-5.08 to -3.89) |
| Maldives | 2249 (1824 to 2836) | 1752.65 (1420.92 to 2209.64) | 174 (132 to 220) | 133.57 (101.18 to 168.87) | -7.19 (-7.82 to -6.56) |
| Mali | 296951 (210697 to 400551) | 6004.02 (4260.06 to 8098.71) | 165152 (117978 to 223761) | 1160.34 (828.90 to 1572.12) | -5.15 (-6.05 to -4.24) |
| Malta | 84 (74 to 95) | 73.28 (64.11 to 82.64) | 21 (17 to 25) | 24.61 (19.67 to 30.09) | -3.60 (-4.01 to -3.19) |
| Marshall Islands | 431 (334 to 533) | 1609.29 (1247.55 to 1988.75) | 157 (109 to 207) | 681.77 (473.85 to 898.13) | -3.44 (-4.27 to -2.60) |
| Mauritania | 41056 (31024 to 51071) | 3620.70 (2735.96 to 4503.94) | 12178 (9214 to 15494) | 522.19 (395.08 to 664.34) | -5.89 (-6.73 to -5.05) |
| Mauritius | 1804 (1673 to 1965) | 418.63 (388.36 to 456.08) | 375 (324 to 426) | 126.36 (109.10 to 143.42) | -3.10 (-3.33 to -2.87) |
| Mexico | 748118 (679905 to 833590) | 1725.08 (1567.79 to 1922.17) | 42368 (31265 to 57282) | 98.19 (72.46 to 132.75) | -9.29 (-9.50 to -9.08) |
| Micronesia (Federated States of) | 1406 (1094 to 1733) | 2445.66 (1902.31 to 3013.28) | 169 (119 to 219) | 408.95 (287.43 to 527.82) | -6.42 (-6.64 to -6.20) |
| Monaco | 3 (2 to 4) | 65.89 (48.57 to 88.46) | 1 (1 to 1) | 14.15 (11.64 to 17.31) | -6.93 (-7.57 to -6.29) |
| Mongolia | 108407 (92138 to 125961) | 9550.27 (8117.03 to 11096.72) | 6862 (5080 to 9053) | 522.75 (386.95 to 689.64) | -8.90 (-9.18 to -8.62) |
| Montenegro | 471 (352 to 622) | 219.35 (164.15 to 289.72) | 36 (26 to 47) | 23.67 (17.37 to 31.17) | -6.94 (-7.59 to -6.27) |
| Morocco | 301330 (234787 to 375789) | 2414.53 (1881.33 to 3011.17) | 11754 (7462 to 16721) | 91.01 (57.77 to 129.46) | -9.85 (-10.67 to -9.03) |
| Mozambique | 587270 (418166 to 855150) | 7757.14 (5523.47 to 11295.51) | 116714 (85963 to 160187) | 657.39 (484.19 to 902.25) | -7.32 (-8.08 to -6.56) |
| Myanmar | 1513328 (1094324 to 1970281) | 7918.52 (5726.07 to 10309.53) | 196420 (146097 to 252474) | 945.78 (703.47 to 1215.68) | -6.72 (-7.34 to -6.10) |
| Namibia | 16690 (12833 to 21081) | 2191.15 (1684.85 to 2767.74) | 5780 (3912 to 8208) | 539.52 (365.09 to 766.14) | -3.79 (-4.50 to -3.08) |
| Nauru | 129 (101 to 160) | 2485.32 (1945.87 to 3064.61) | 73 (52 to 98) | 1424.10 (1018.72 to 1911.78) | -2.34 (-3.50 to -1.16) |
| Nepal | 841740 (663984 to 998283) | 8133.69 (6416.04 to 9646.35) | 53694 (39679 to 72455) | 431.12 (318.59 to 581.76) | -9.01 (-9.45 to -8.56) |
| Netherlands | 2643 (2463 to 2828) | 68.95 (64.27 to 73.77) | 280 (248 to 312) | 7.59 (6.71 to 8.46) | -8.11 (-8.69 to -7.54) |
| New Zealand | 1408 (1293 to 1531) | 127.86 (117.43 to 139.04) | 131 (114 to 147) | 10.03 (8.75 to 11.28) | -8.05 (-8.40 to -7.69) |
| Nicaragua | 55223 (46749 to 64284) | 2454.07 (2077.48 to 2856.73) | 2744 (1953 to 3762) | 105.58 (75.13 to 144.75) | -9.57 (-9.99 to -9.14) |
| Niger | 1014599 (732150 to 1308419) | 20912.69 (15090.92 to 26968.85) | 342344 (221182 to 499553) | 2196.38 (1419.03 to 3204.98) | -7.42 (-7.86 to -6.98) |
| Nigeria | 5456692 (4168087 to 6823830) | 11246.51 (8590.63 to 14064.25) | 3227316 (1991012 to 4573755) | 2525.83 (1558.25 to 3579.61) | -4.22 (-4.71 to -3.73) |
| Niue | 12 (9 to 15) | 1150.11 (917.74 to 1438.99) | 7 (6 to 8) | 1294.75 (1148.80 to 1453.41) | -3.45 (-4.63 to -2.26) |
| North Macedonia | 6563 (5244 to 8236) | 941.73 (752.45 to 1181.75) | 168 (125 to 214) | 37.54 (27.92 to 47.84) | -8.84 (-9.57 to -8.09) |
| Northern Mariana Islands | 56 (43 to 73) | 347.28 (264.16 to 450.52) | 21 (17 to 26) | 140.19 (111.92 to 174.41) | -2.87 (-3.25 to -2.49) |
| Norway | 676 (628 to 723) | 60.80 (56.49 to 65.00) | 53 (48 to 59) | 4.29 (3.83 to 4.76) | -9.02 (-9.43 to -8.61) |
| Oman | 9747 (7159 to 14426) | 972.48 (714.27 to 1439.27) | 851 (691 to 1037) | 57.07 (46.32 to 69.54) | -7.39 (-8.17 to -6.60) |
| Pakistan | 2439842 (2005965 to 2899044) | 4002.15 (3290.45 to 4755.39) | 943353 (712287 to 1229086) | 863.69 (652.14 to 1125.29) | -3.84 (-4.40 to -3.29) |
| Palau | 81 (60 to 105) | 1315.72 (969.10 to 1707.90) | 14 (11 to 17) | 312.19 (247.91 to 387.34) | -5.14 (-5.53 to -4.75) |
| Palestine | 14153 (10661 to 18583) | 1185.80 (893.21 to 1556.91) | 1917 (1495 to 2451) | 79.61 (62.07 to 101.79) | -8.18 (-8.88 to -7.47) |
| Panama | 6467 (5572 to 7505) | 594.95 (512.59 to 690.40) | 2122 (1669 to 2771) | 140.77 (110.72 to 183.82) | -4.25 (-4.88 to -3.61) |
| Papua New Guinea | 132596 (103049 to 169244) | 6229.14 (4841.09 to 7950.80) | 130462 (98212 to 169223) | 2663.00 (2004.71 to 3454.21) | -2.49 (-2.94 to -2.05) |
| Paraguay | 18647 (14704 to 22925) | 901.21 (710.66 to 1107.96) | 2723 (1857 to 3808) | 101.95 (69.55 to 142.60) | -6.90 (-7.58 to -6.21) |
| Peru | 401962 (342164 to 467522) | 3774.39 (3212.89 to 4390.00) | 23589 (16293 to 32320) | 188.55 (130.24 to 258.35) | -9.12 (-9.36 to -8.88) |
| Philippines | 998942 (841260 to 1217242) | 3116.57 (2624.62 to 3797.64) | 221699 (178658 to 274317) | 495.11 (398.99 to 612.62) | -4.93 (-5.31 to -4.55) |
| Poland | 25363 (23705 to 27075) | 203.73 (190.41 to 217.47) | 2817 (2449 to 3174) | 36.60 (31.81 to 41.23) | -4.19 (-4.65 to -3.73) |
| Portugal | 5050 (4728 to 5366) | 169.40 (158.61 to 180.00) | 283 (247 to 319) | 14.78 (12.89 to 16.63) | -8.29 (-8.74 to -7.83) |
| Puerto Rico | 1965 (1788 to 2155) | 147.50 (134.19 to 161.79) | 92 (78 to 111) | 14.10 (11.96 to 16.96) | -8.15 (-8.76 to -7.53) |
| Qatar | 567 (424 to 738) | 370.40 (276.74 to 481.98) | 134 (103 to 171) | 22.37 (17.19 to 28.68) | -8.59 (-8.86 to -8.33) |
| Republic of Korea | 29315 (24676 to 35438) | 184.06 (154.93 to 222.50) | 636 (539 to 771) | 7.59 (6.43 to 9.19) | -9.63 (-9.88 to -9.37) |
| Republic of Moldova | 18260 (16097 to 20790) | 1154.78 (1017.95 to 1314.75) | 890 (688 to 1140) | 127.77 (98.75 to 163.80) | -6.00 (-6.43 to -5.57) |
| Romania | 153757 (143561 to 162844) | 2049.31 (1913.41 to 2170.42) | 6832 (5977 to 7754) | 169.07 (147.92 to 191.89) | -6.90 (-7.4 to -6.41) |
| Russian Federation | 229268 (221292 to 237603) | 507.40 (489.75 to 525.84) | 20727 (19018 to 22265) | 61.34 (56.28 to 65.89) | -5.73 (-6.57 to -4.89) |
| Rwanda | 402428 (282293 to 523076) | 9723.58 (6820.85 to 12638.73) | 28981 (21422 to 37131) | 450.14 (332.74 to 576.74) | -11.06 (-12.07 to -10.04) |
| Saint Kitts and Nevis | 91 (81 to 103) | 495.45 (440.06 to 560.39) | 26 (20 to 33) | 183.54 (142.59 to 236.07) | -3.47 (-3.77 to -3.16) |
| Saint Lucia | 240 (197 to 285) | 359.39 (294.42 to 426.83) | 46 (33 to 62) | 108.51 (78.47 to 148.28) | -4.05 (-4.21 to -3.88) |
| Saint Vincent and the Grenadines | 231 (188 to 280) | 430.24 (350.34 to 521.81) | 44 (35 to 57) | 130.64 (102.59 to 168.43) | -3.96 (-4.25 to -3.66) |
| Samoa | 1449 (1037 to 1923) | 1581.91 (1132.55 to 2099.20) | 595 (387 to 855) | 588.96 (383.10 to 847.34) | -3.36 (-3.50 to -3.22) |
| San Marino | 3 (2 to 4) | 52.12 (40.00 to 69.89) | 0 (0 to 0) | 4.77 (3.33 to 6.59) | -7.35 (-7.60 to -7.10) |
| Sao Tome and Principe | 3391 (2605 to 4135) | 4852.99 (3728.15 to 5916.24) | 208 (145 to 280) | 204.29 (142.22 to 275.27) | -9.97 (-10.64 to -9.29) |
| Saudi Arabia | 81598 (61356 to 114449) | 993.72 (747.21 to 1393.78) | 2528 (1920 to 3236) | 24.95 (18.95 to 31.93) | -11.27 (-11.63 to -10.91) |
| Senegal | 255259 (196911 to 318009) | 5740.11 (4428.02 to 7151.18) | 38639 (30046 to 47677) | 476.13 (370.24 to 587.49) | -7.44 (-8.26 to -6.60) |
| Serbia | 12034 (9450 to 16352) | 414.81 (325.72 to 563.64) | 383 (300 to 484) | 20.44 (16.03 to 25.83) | -9.40 (-9.92 to -8.89) |
| Seychelles | 167 (143 to 195) | 536.08 (456.39 to 622.80) | 88 (71 to 111) | 289.11 (231.78 to 364.7) | -0.46 (-0.85 to -0.06) |
| Sierra Leone | 286071 (200757 to 384609) | 12969.59 (9101.70 to 17437.02) | 49083 (34422 to 69244) | 1078.59 (756.43 to 1521.63) | -8.20 (-8.86 to -7.53) |
| Singapore | 2788 (2586 to 2990) | 302.69 (280.77 to 324.64) | 310 (274 to 351) | 29.71 (26.27 to 33.6) | -6.95 (-7.35 to -6.56) |
| Slovakia | 8973 (8091 to 9885) | 511.24 (460.97 to 563.16) | 734 (589 to 885) | 65.51 (52.51 to 78.9) | -6.60 (-7.01 to -6.19) |
| Slovenia | 1261 (1142 to 1381) | 226.50 (205.05 to 247.96) | 140 (120 to 160) | 34.47 (29.72 to 39.55) | -5.81 (-6.03 to -5.58) |
| Solomon Islands | 6406 (4703 to 8407) | 3291.37 (2416.37 to 4319.95) | 2666 (2071 to 3406) | 806.15 (626.15 to 1029.84) | -4.75 (-5.36 to -4.13) |
| Somalia | 379043 (252035 to 521606) | 8009.63 (5325.81 to 11022.17) | 348693 (227849 to 490796) | 2727.49 (1782.24 to 3839.03) | -2.83 (-3.23 to -2.43) |
| South Africa | 554934 (471826 to 670610) | 3149.15 (2677.53 to 3805.59) | 112666 (89425 to 133863) | 565.65 (448.96 to 672.07) | -5.15 (-5.89 to -4.40) |
| South Sudan | 281214 (211361 to 374161) | 8557.76 (6432.03 to 11386.28) | 206317 (143030 to 311880) | 3759.87 (2606.54 to 5683.62) | -2.25 (-2.68 to -1.81) |
| Spain | 6674 (6309 to 7047) | 59.87 (56.59 to 63.22) | 632 (566 to 705) | 7.18 (6.42 to 8.00) | -6.33 (-6.79 to -5.87) |
| Sri Lanka | 28501 (25002 to 31948) | 393.65 (345.32 to 441.26) | 5695 (4197 to 7631) | 82.53 (60.82 to 110.59) | -4.59 (-5.11 to -4.05) |
| Sudan | 706618 (524636 to 1039842) | 6396.97 (4749.50 to 9413.63) | 70057 (50993 to 96643) | 327.75 (238.56 to 452.13) | -9.04 (-9.75 to -8.32) |
| Suriname | 1532 (1168 to 1893) | 895.93 (683.05 to 1107.16) | 484 (341 to 677) | 255.19 (179.69 to 356.76) | -4.10 (-4.30 to -3.90) |
| Sweden | 1022 (948 to 1096) | 48.50 (45.00 to 52.00) | 103 (91 to 117) | 4.27 (3.77 to 4.83) | -7.41 (-7.82 to -6.99) |
| Switzerland | 1294 (1195 to 1410) | 82.07 (75.80 to 89.41) | 128 (112 to 146) | 7.28 (6.37 to 8.27) | -7.78 (-7.92 to -7.63) |
| Syrian Arab Republic | 100957 (75268 to 134164) | 1373.21 (1023.78 to 1824.89) | 9724 (7288 to 12339) | 178.74 (133.97 to 226.82) | -4.52 (-5.34 to -3.69) |
| Taiwan (Province of China) | 17945 (16977 to 18982) | 245.52 (232.28 to 259.70) | 870 (768 to 981) | 21.50 (18.99 to 24.25) | -8.11 (-8.48 to -7.73) |
| Tajikistan | 222226 (189784 to 254876) | 7731.01 (6602.39 to 8866.84) | 84952 (59222 to 113086) | 1906.84 (1329.29 to 2538.34) | -4.51 (-4.85 to -4.16) |
| Thailand | 132841 (103800 to 168826) | 584.00 (456.33 to 742.20) | 24998 (21008 to 29175) | 183.16 (153.93 to 213.77) | -3.58 (-3.81 to -3.35) |
| Timor-Leste | 47708 (39175 to 59646) | 11731.46 (9633.20 to 14667.10) | 10678 (8054 to 13594) | 1557.82 (1175.07 to 1983.32) | -7.03 (-7.43 to -6.62) |
| Togo | 107382 (80944 to 137058) | 4988.20 (3760.06 to 6366.71) | 31671 (21292 to 43219) | 758.51 (509.95 to 1035.09) | -5.51 (-6.30 to -4.72) |
| Tokelau | 11 (8 to 14) | 1465.06 (1091.76 to 1873.28) | 12 (9 to 15) | 2403.01 (1738.59 to 2924.42) | -2.94 (-4.62 to -1.24) |
| Tonga | 732 (569 to 898) | 1372.25 (1066.85 to 1683.35) | 266 (198 to 353) | 536.56 (400.33 to 711.69) | -3.08 (-3.52 to -2.65) |
| Trinidad and Tobago | 1882 (1574 to 2206) | 362.23 (302.97 to 424.65) | 249 (188 to 334) | 68.42 (51.61 to 91.82) | -5.06 (-5.65 to -4.47) |
| Tunisia | 55826 (38354 to 89140) | 1399.14 (961.23 to 2234.06) | 3814 (2531 to 5427) | 106.38 (70.59 to 151.37) | -7.11 (-7.51 to -6.71) |
| Turkmenistan | 124921 (110423 to 142573) | 6626.26 (5857.27 to 7562.60) | 22941 (18818 to 27827) | 1170.11 (959.84 to 1419.32) | -5.31 (-5.70 to -4.92) |
| Tuvalu | 308 (234 to 407) | 7205.40 (5486.26 to 9525.01) | 40 (30 to 51) | 811.23 (605.47 to 1045.98) | -6.92 (-7.24 to -6.60) |
| Türkiye | 624947 (470066 to 858343) | 2342.24 (1761.76 to 3216.98) | 10967 (8788 to 13556) | 44.38 (35.56 to 54.86) | -12.63 (-13.12 to -12.13) |
| Uganda | 520051 (344716 to 768226) | 5050.12 (3347.48 to 7460.11) | 168843 (110249 to 237196) | 676.90 (441.99 to 950.93) | -6.48 (-7.05 to -5.91) |
| Ukraine | 44545 (38480 to 50780) | 295.95 (255.65 to 337.37) | 5922 (4995 to 6886) | 70.13 (59.15 to 81.54) | -3.81 (-4.21 to -3.41) |
| United Arab Emirates | 3789 (2889 to 4863) | 535.90 (408.56 to 687.88) | 470 (379 to 581) | 27.76 (22.33 to 34.29) | -8.01 (-8.64 to -7.38) |
| United Kingdom | 13722 (13250 to 14216) | 92.50 (89.32 to 95.83) | 1491 (1338 to 1622) | 9.52 (8.54 to 10.36) | -7.89 (-8.41 to -7.36) |
| United Republic of Tanzania | 1301583 (965801 to 1654771) | 8721.86 (6471.80 to 11088.56) | 231976 (164753 to 307331) | 751.75 (533.90 to 995.94) | -7.53 (-8.17 to -6.88) |
| United States Virgin Islands | 77 (57 to 98) | 183.61 (136.96 to 235.05) | 3 (2 to 5) | 18.91 (13.01 to 25.8) | -7.21 (-7.44 to -6.99) |
| United States of America | 58480 (56372 to 60754) | 78.98 (76.13 to 82.05) | 8073 (7374 to 8774) | 9.93 (9.07 to 10.79) | -7.01 (-7.37 to -6.64) |
| Uruguay | 3573 (3264 to 3896) | 331.75 (303.06 to 361.72) | 308 (256 to 365) | 34.07 (28.32 to 40.38) | -8.03 (-8.60 to -7.46) |
| Uzbekistan | 535641 (494125 to 584492) | 5017.34 (4628.46 to 5474.92) | 92123 (75605 to 112031) | 730.54 (599.56 to 888.42) | -5.66 (-6.44 to -4.87) |
| Vanuatu | 1755 (1278 to 2269) | 2102.78 (1530.97 to 2719.03) | 1314 (946 to 1741) | 890.88 (641.69 to 1181.04) | -2.60 (-3.23 to -1.98) |
| Venezuela (Bolivarian Republic of) | 76058 (71285 to 81022) | 838.01 (785.41 to 892.70) | 21706 (15966 to 29210) | 248.01 (182.44 to 333.76) | -2.76 (-3.33 to -2.19) |
| Viet Nam | 532988 (413235 to 648672) | 1583.12 (1227.42 to 1926.73) | 77431 (57683 to 100300) | 244.89 (182.43 to 317.22) | -5.07 (-5.45 to -4.68) |
| Yemen | 426982 (312686 to 632580) | 5078.23 (3718.87 to 7523.46) | 68469 (49186 to 90396) | 391.87 (281.51 to 517.37) | -8.06 (-8.55 to -7.58) |
| Zambia | 419633 (327729 to 550657) | 8927.42 (6972.22 to 11714.88) | 65061 (43342 to 87060) | 622.34 (414.59 to 832.77) | -8.31 (-9.18 to -7.44) |
| Zimbabwe | 170056 (143953 to 198565) | 2823.74 (2390.30 to 3297.13) | 105598 (81130 to 130123) | 1322.58 (1016.13 to 1629.75) | -2.06 (-2.78 to -1.33) |
| The EAPC was calculated using 31 years of data from 1990-2021. The table only includes data for 1990 and 2021 for comparative analysis. UI, uncertainty interval. ASR, age-standardized rate per 100,000. EAPC, estimated annual percentage change of ASR. CI, confidence interval. | | | | | |

**Supplementary Table S3. Global estimates of deaths and DALYs from pneumococcal disease among children and adolescents aged <20 years by age group in 1990 and 2021, including counts, age-standardized rates per 100,000 population, and EAPCs.**

|  | **1990** | | **2021** | |  |
| --- | --- | --- | --- | --- | --- |
| **location** | **Counts (95% *UI*)** | **ASR per 100,000 (95% *UI*)** | **Counts (95% *UI*)** | **ASR per 100,000 (95% *UI*)** | **EAPC_95%*CI*** |
| **Deaths** |  |  |  |  |  |
| <5 years | 764491 (654612 to 886053) | 123.32 (105.59 to 142.93) | 154773 (119980 to 189742) | 23.52 (18.23 to 28.83) | -5.02 (-5.40 to 4.63) |
| 5-9 years | 30445 (25371 to 34634) | 5.22 (4.35 to 5.94) | 11076 (9250 to 12975) | 1.61 (1.35 to 1.89) | -3.53 (-3.83 to 3.22) |
| 10-14 years | 12757 (10851 to 13931) | 2.38 (2.03 to 2.60) | 7191 (6264 to 8051) | 1.08 (0.94 to 1.21) | -2.37 (-2.59 to 2.15) |
| 15-19 years | 9525 (8635 to 10256) | 1.83 (1.66 to 1.97) | 6314 (5557 to 7074) | 1.01 (0.89 to 1.13) | -2.04 (-2.22 to 1.87) |
| **DALYs** |  |  |  |  |  |
| <5 years | 67956332 (58256175 to 78699803) | 10961.80 (9397.10 to 12694.79) | 13769828 (10694852 to 16868531) | 2092.13 (1624.93 to 2562.94) | -5.02 (-5.40 to 4.63) |
| 5-9 years | 2557326 (2134096 to 2911429) | 438.25 (365.72 to 498.93) | 939006 (789260 to 1095433) | 136.67 (114.88 to 159.44) | -3.51 (-3.81 to 3.20) |
| 10-14 years | 1011831 (864541 to 1104663) | 188.89 (161.39 to 206.22) | 575155 (501740 to 641662) | 86.28 (75.26 to 96.25) | -2.35 (-2.57 to 2.14) |
| 15-19 years | 710092 (647130 to 764523) | 136.71 (124.59 to 147.19) | 473839 (418419 to 529359) | 75.94 (67.06 to 84.84) | -2.03 (-2.20 to 1.86) |
| The EAPC was calculated using 31 years of data from 1990-2021. The table only includes data for 1990 and 2021 for comparative analysis. UI, uncertainty interval. ASR, age-standardized rate per 100,000. EAPC, estimated annual percentage change of ASR. CI, confidence interval. | | | | | |

**Supplementary Table S4. Projected ASMRs and ASDRs of pneumococcal disease among children and adolescents aged <20 years globally by sex, 1990–2036.**

| **Year** | **Mean ASR** | **SD** | **95% *UI* Lower** | **95% *UI* Upper** | **Metric** | **Sex** |
| --- | --- | --- | --- | --- | --- | --- |
| **Death** |  |  |  |  |  |  |
| 1990 | 33.20 | 0.04 | 33.12 | 33.27 | ASR | Both |
| 1991 | 32.33 | 0.04 | 32.26 | 32.40 | ASR | Both |
| 1992 | 31.61 | 0.04 | 31.54 | 31.68 | ASR | Both |
| 1993 | 30.85 | 0.04 | 30.78 | 30.92 | ASR | Both |
| 1994 | 30.05 | 0.03 | 29.98 | 30.12 | ASR | Both |
| 1995 | 29.45 | 0.03 | 29.38 | 29.51 | ASR | Both |
| 1996 | 28.53 | 0.03 | 28.47 | 28.60 | ASR | Both |
| 1997 | 27.63 | 0.03 | 27.56 | 27.69 | ASR | Both |
| 1998 | 26.62 | 0.03 | 26.56 | 26.69 | ASR | Both |
| 1999 | 25.69 | 0.03 | 25.62 | 25.75 | ASR | Both |
| 2000 | 24.90 | 0.03 | 24.84 | 24.96 | ASR | Both |
| 2001 | 24.01 | 0.03 | 23.95 | 24.07 | ASR | Both |
| 2002 | 23.15 | 0.03 | 23.09 | 23.21 | ASR | Both |
| 2003 | 22.19 | 0.03 | 22.13 | 22.25 | ASR | Both |
| 2004 | 21.27 | 0.03 | 21.21 | 21.33 | ASR | Both |
| 2005 | 20.46 | 0.03 | 20.40 | 20.51 | ASR | Both |
| 2006 | 19.66 | 0.03 | 19.61 | 19.72 | ASR | Both |
| 2007 | 18.80 | 0.03 | 18.75 | 18.85 | ASR | Both |
| 2008 | 17.88 | 0.03 | 17.83 | 17.93 | ASR | Both |
| 2009 | 16.97 | 0.03 | 16.92 | 17.02 | ASR | Both |
| 2010 | 16.06 | 0.02 | 16.01 | 16.10 | ASR | Both |
| 2011 | 15.13 | 0.02 | 15.09 | 15.18 | ASR | Both |
| 2012 | 14.31 | 0.02 | 14.26 | 14.36 | ASR | Both |
| 2013 | 13.55 | 0.02 | 13.50 | 13.59 | ASR | Both |
| 2014 | 12.73 | 0.02 | 12.69 | 12.78 | ASR | Both |
| 2015 | 11.60 | 0.02 | 11.55 | 11.64 | ASR | Both |
| 2016 | 10.62 | 0.02 | 10.59 | 10.66 | ASR | Both |
| 2017 | 9.67 | 0.02 | 9.64 | 9.71 | ASR | Both |
| 2018 | 8.78 | 0.02 | 8.75 | 8.82 | ASR | Both |
| 2019 | 8.03 | 0.02 | 8.00 | 8.06 | ASR | Both |
| 2020 | 7.24 | 0.02 | 7.21 | 7.27 | ASR | Both |
| 2021 | 6.80 | 0.02 | 6.77 | 6.83 | ASR | Both |
| 2022 | 6.14 | 0.19 | 5.77 | 6.51 | ASR | Both |
| 2023 | 5.59 | 0.21 | 5.17 | 6.01 | ASR | Both |
| 2024 | 5.08 | 0.24 | 4.61 | 5.55 | ASR | Both |
| 2025 | 4.62 | 0.27 | 4.09 | 5.14 | ASR | Both |
| 2026 | 4.19 | 0.29 | 3.62 | 4.77 | ASR | Both |
| 2027 | 3.81 | 0.32 | 3.19 | 4.43 | ASR | Both |
| 2028 | 3.46 | 0.34 | 2.80 | 4.12 | ASR | Both |
| 2029 | 3.14 | 0.36 | 2.44 | 3.84 | ASR | Both |
| 2030 | 2.85 | 0.37 | 2.13 | 3.57 | ASR | Both |
| 2031 | 2.59 | 0.38 | 1.84 | 3.33 | ASR | Both |
| 2032 | 2.35 | 0.39 | 1.59 | 3.10 | ASR | Both |
| 2033 | 2.13 | 0.39 | 1.36 | 2.90 | ASR | Both |
| 2034 | 1.93 | 0.39 | 1.16 | 2.70 | ASR | Both |
| 2035 | 1.75 | 0.39 | 0.98 | 2.52 | ASR | Both |
| 2036 | 1.59 | 0.39 | 0.82 | 2.35 | ASR | Both |
| 1990 | 32.79 | 0.05 | 32.69 | 32.89 | ASR | Male |
| 1991 | 31.94 | 0.05 | 31.85 | 32.04 | ASR | Male |
| 1992 | 31.24 | 0.05 | 31.15 | 31.34 | ASR | Male |
| 1993 | 30.54 | 0.05 | 30.45 | 30.64 | ASR | Male |
| 1994 | 29.80 | 0.05 | 29.70 | 29.89 | ASR | Male |
| 1995 | 29.18 | 0.05 | 29.09 | 29.28 | ASR | Male |
| 1996 | 28.27 | 0.05 | 28.18 | 28.36 | ASR | Male |
| 1997 | 27.37 | 0.05 | 27.27 | 27.46 | ASR | Male |
| 1998 | 26.35 | 0.05 | 26.26 | 26.44 | ASR | Male |
| 1999 | 25.38 | 0.04 | 25.29 | 25.47 | ASR | Male |
| 2000 | 24.61 | 0.04 | 24.53 | 24.70 | ASR | Male |
| 2001 | 23.77 | 0.04 | 23.69 | 23.86 | ASR | Male |
| 2002 | 22.92 | 0.04 | 22.84 | 23.00 | ASR | Male |
| 2003 | 21.96 | 0.04 | 21.88 | 22.04 | ASR | Male |
| 2004 | 21.05 | 0.04 | 20.97 | 21.13 | ASR | Male |
| 2005 | 20.25 | 0.04 | 20.18 | 20.33 | ASR | Male |
| 2006 | 19.46 | 0.04 | 19.39 | 19.54 | ASR | Male |
| 2007 | 18.61 | 0.04 | 18.54 | 18.69 | ASR | Male |
| 2008 | 17.69 | 0.04 | 17.61 | 17.76 | ASR | Male |
| 2009 | 16.78 | 0.04 | 16.71 | 16.85 | ASR | Male |
| 2010 | 15.88 | 0.03 | 15.81 | 15.95 | ASR | Male |
| 2011 | 14.97 | 0.03 | 14.90 | 15.03 | ASR | Male |
| 2012 | 14.18 | 0.03 | 14.12 | 14.25 | ASR | Male |
| 2013 | 13.43 | 0.03 | 13.36 | 13.49 | ASR | Male |
| 2014 | 12.63 | 0.03 | 12.57 | 12.68 | ASR | Male |
| 2015 | 11.52 | 0.03 | 11.46 | 11.57 | ASR | Male |
| 2016 | 10.56 | 0.03 | 10.51 | 10.61 | ASR | Male |
| 2017 | 9.63 | 0.03 | 9.58 | 9.68 | ASR | Male |
| 2018 | 8.75 | 0.02 | 8.70 | 8.80 | ASR | Male |
| 2019 | 8.01 | 0.02 | 7.97 | 8.06 | ASR | Male |
| 2020 | 7.41 | 0.02 | 7.37 | 7.45 | ASR | Male |
| 2021 | 6.97 | 0.02 | 6.92 | 7.01 | ASR | Male |
| 2022 | 6.32 | 0.20 | 5.93 | 6.71 | ASR | Male |
| 2023 | 5.78 | 0.23 | 5.34 | 6.23 | ASR | Male |
| 2024 | 5.29 | 0.25 | 4.80 | 5.79 | ASR | Male |
| 2025 | 4.84 | 0.28 | 4.29 | 5.40 | ASR | Male |
| 2026 | 4.43 | 0.31 | 3.82 | 5.04 | ASR | Male |
| 2027 | 4.05 | 0.34 | 3.39 | 4.72 | ASR | Male |
| 2028 | 3.71 | 0.36 | 3.00 | 4.41 | ASR | Male |
| 2029 | 3.39 | 0.38 | 2.64 | 4.14 | ASR | Male |
| 2030 | 3.10 | 0.40 | 2.32 | 3.88 | ASR | Male |
| 2031 | 2.83 | 0.41 | 2.02 | 3.64 | ASR | Male |
| 2032 | 2.59 | 0.42 | 1.76 | 3.42 | ASR | Male |
| 2033 | 2.36 | 0.43 | 1.52 | 3.21 | ASR | Male |
| 2034 | 2.16 | 0.43 | 1.31 | 3.01 | ASR | Male |
| 2035 | 1.97 | 0.44 | 1.12 | 2.83 | ASR | Male |
| 2036 | 1.80 | 0.44 | 0.95 | 2.66 | ASR | Male |
| 1990 | 33.63 | 0.05 | 33.52 | 33.73 | ASR | Female |
| 1991 | 32.74 | 0.05 | 32.63 | 32.84 | ASR | Female |
| 1992 | 32.01 | 0.05 | 31.91 | 32.11 | ASR | Female |
| 1993 | 31.17 | 0.05 | 31.07 | 31.27 | ASR | Female |
| 1994 | 30.33 | 0.05 | 30.23 | 30.43 | ASR | Female |
| 1995 | 29.72 | 0.05 | 29.63 | 29.82 | ASR | Female |
| 1996 | 28.81 | 0.05 | 28.72 | 28.91 | ASR | Female |
| 1997 | 27.91 | 0.05 | 27.81 | 28.00 | ASR | Female |
| 1998 | 26.91 | 0.05 | 26.82 | 27.01 | ASR | Female |
| 1999 | 26.01 | 0.05 | 25.92 | 26.11 | ASR | Female |
| 2000 | 25.20 | 0.05 | 25.11 | 25.29 | ASR | Female |
| 2001 | 24.27 | 0.05 | 24.18 | 24.36 | ASR | Female |
| 2002 | 23.41 | 0.04 | 23.32 | 23.49 | ASR | Female |
| 2003 | 22.45 | 0.04 | 22.36 | 22.53 | ASR | Female |
| 2004 | 21.50 | 0.04 | 21.42 | 21.59 | ASR | Female |
| 2005 | 20.68 | 0.04 | 20.60 | 20.76 | ASR | Female |
| 2006 | 19.88 | 0.04 | 19.80 | 19.95 | ASR | Female |
| 2007 | 19.00 | 0.04 | 18.93 | 19.08 | ASR | Female |
| 2008 | 18.09 | 0.04 | 18.01 | 18.16 | ASR | Female |
| 2009 | 17.18 | 0.04 | 17.11 | 17.25 | ASR | Female |
| 2010 | 16.24 | 0.04 | 16.17 | 16.31 | ASR | Female |
| 2011 | 15.31 | 0.03 | 15.24 | 15.38 | ASR | Female |
| 2012 | 14.44 | 0.03 | 14.38 | 14.51 | ASR | Female |
| 2013 | 13.68 | 0.03 | 13.61 | 13.74 | ASR | Female |
| 2014 | 12.85 | 0.03 | 12.78 | 12.91 | ASR | Female |
| 2015 | 11.68 | 0.03 | 11.62 | 11.74 | ASR | Female |
| 2016 | 10.69 | 0.03 | 10.64 | 10.75 | ASR | Female |
| 2017 | 9.72 | 0.03 | 9.67 | 9.78 | ASR | Female |
| 2018 | 8.82 | 0.03 | 8.77 | 8.87 | ASR | Female |
| 2019 | 8.05 | 0.02 | 8.01 | 8.10 | ASR | Female |
| 2020 | 7.07 | 0.02 | 7.02 | 7.11 | ASR | Female |
| 2021 | 6.62 | 0.02 | 6.57 | 6.66 | ASR | Female |
| 2022 | 5.95 | 0.19 | 5.58 | 6.32 | ASR | Female |
| 2023 | 5.37 | 0.21 | 4.96 | 5.79 | ASR | Female |
| 2024 | 4.85 | 0.24 | 4.38 | 5.32 | ASR | Female |
| 2025 | 4.37 | 0.27 | 3.85 | 4.90 | ASR | Female |
| 2026 | 3.95 | 0.29 | 3.37 | 4.52 | ASR | Female |
| 2027 | 3.56 | 0.31 | 2.94 | 4.17 | ASR | Female |
| 2028 | 3.21 | 0.33 | 2.56 | 3.86 | ASR | Female |
| 2029 | 2.89 | 0.35 | 2.21 | 3.57 | ASR | Female |
| 2030 | 2.60 | 0.36 | 1.90 | 3.30 | ASR | Female |
| 2031 | 2.34 | 0.36 | 1.63 | 3.06 | ASR | Female |
| 2032 | 2.11 | 0.37 | 1.38 | 2.83 | ASR | Female |
| 2033 | 1.90 | 0.37 | 1.17 | 2.62 | ASR | Female |
| 2034 | 1.71 | 0.37 | 0.98 | 2.43 | ASR | Female |
| 2035 | 1.53 | 0.37 | 0.81 | 2.25 | ASR | Female |
| 2036 | 1.38 | 0.36 | 0.67 | 2.09 | ASR | Female |
| **DALYs** |  |  |  |  |  |  |
| 1990 | 2932.09 | 0.35 | 2931.41 | 2932.77 | ASR | Both |
| 1991 | 2854.94 | 0.34 | 2854.28 | 2855.61 | ASR | Both |
| 1992 | 2791.63 | 0.34 | 2790.97 | 2792.29 | ASR | Both |
| 1993 | 2723.72 | 0.33 | 2723.07 | 2724.38 | ASR | Both |
| 1994 | 2653.45 | 0.33 | 2652.81 | 2654.09 | ASR | Both |
| 1995 | 2599.76 | 0.33 | 2599.13 | 2600.40 | ASR | Both |
| 1996 | 2519.13 | 0.32 | 2518.50 | 2519.76 | ASR | Both |
| 1997 | 2438.93 | 0.32 | 2438.31 | 2439.55 | ASR | Both |
| 1998 | 2350.11 | 0.31 | 2349.51 | 2350.72 | ASR | Both |
| 1999 | 2267.31 | 0.31 | 2266.71 | 2267.91 | ASR | Both |
| 2000 | 2197.71 | 0.30 | 2197.12 | 2198.30 | ASR | Both |
| 2001 | 2119.72 | 0.30 | 2119.14 | 2120.30 | ASR | Both |
| 2002 | 2044.01 | 0.29 | 2043.44 | 2044.58 | ASR | Both |
| 2003 | 1959.21 | 0.28 | 1958.65 | 1959.76 | ASR | Both |
| 2004 | 1877.30 | 0.28 | 1876.76 | 1877.84 | ASR | Both |
| 2005 | 1805.51 | 0.27 | 1804.98 | 1806.04 | ASR | Both |
| 2006 | 1734.96 | 0.26 | 1734.44 | 1735.48 | ASR | Both |
| 2007 | 1658.82 | 0.26 | 1658.32 | 1659.32 | ASR | Both |
| 2008 | 1577.29 | 0.25 | 1576.80 | 1577.78 | ASR | Both |
| 2009 | 1496.96 | 0.24 | 1496.49 | 1497.43 | ASR | Both |
| 2010 | 1415.72 | 0.23 | 1415.26 | 1416.17 | ASR | Both |
| 2011 | 1334.15 | 0.23 | 1333.71 | 1334.59 | ASR | Both |
| 2012 | 1261.18 | 0.22 | 1260.75 | 1261.60 | ASR | Both |
| 2013 | 1193.71 | 0.21 | 1193.3 | 1194.13 | ASR | Both |
| 2014 | 1121.75 | 0.21 | 1121.34 | 1122.15 | ASR | Both |
| 2015 | 1021.06 | 0.20 | 1020.68 | 1021.45 | ASR | Both |
| 2016 | 935.20 | 0.19 | 934.84 | 935.56 | ASR | Both |
| 2017 | 851.38 | 0.18 | 851.03 | 851.72 | ASR | Both |
| 2018 | 772.60 | 0.17 | 772.27 | 772.93 | ASR | Both |
| 2019 | 706.44 | 0.16 | 706.12 | 706.76 | ASR | Both |
| 2020 | 636.23 | 0.15 | 635.93 | 636.53 | ASR | Both |
| 2021 | 597.93 | 0.15 | 597.63 | 598.22 | ASR | Both |
| 2022 | 539.38 | 17.54 | 505.01 | 573.75 | ASR | Both |
| 2023 | 489.96 | 19.62 | 451.51 | 528.41 | ASR | Both |
| 2024 | 444.91 | 21.98 | 401.83 | 487.99 | ASR | Both |
| 2025 | 403.90 | 24.37 | 356.14 | 451.66 | ASR | Both |
| 2026 | 366.58 | 26.63 | 314.38 | 418.77 | ASR | Both |
| 2027 | 332.58 | 28.67 | 276.39 | 388.78 | ASR | Both |
| 2028 | 301.66 | 30.44 | 242.00 | 361.31 | ASR | Both |
| 2029 | 273.54 | 31.90 | 211.01 | 336.07 | ASR | Both |
| 2030 | 247.99 | 33.07 | 183.18 | 312.80 | ASR | Both |
| 2031 | 224.80 | 33.94 | 158.26 | 291.33 | ASR | Both |
| 2032 | 203.73 | 34.55 | 136.02 | 271.45 | ASR | Both |
| 2033 | 184.63 | 34.90 | 116.22 | 253.03 | ASR | Both |
| 2034 | 167.30 | 35.03 | 98.65 | 235.95 | ASR | Both |
| 2035 | 151.60 | 34.94 | 83.11 | 220.08 | ASR | Both |
| 2036 | 137.37 | 34.68 | 69.41 | 205.33 | ASR | Both |
| 1990 | 2897.63 | 0.48 | 2896.7 | 2898.57 | ASR | Male |
| 1991 | 2822.49 | 0.47 | 2821.57 | 2823.42 | ASR | Male |
| 1992 | 2760.31 | 0.47 | 2759.40 | 2761.22 | ASR | Male |
| 1993 | 2698.24 | 0.46 | 2697.34 | 2699.15 | ASR | Male |
| 1994 | 2632.29 | 0.46 | 2631.39 | 2633.18 | ASR | Male |
| 1995 | 2578.04 | 0.45 | 2577.16 | 2578.92 | ASR | Male |
| 1996 | 2497.52 | 0.44 | 2496.65 | 2498.39 | ASR | Male |
| 1997 | 2417.20 | 0.44 | 2416.34 | 2418.06 | ASR | Male |
| 1998 | 2327.55 | 0.43 | 2326.71 | 2328.39 | ASR | Male |
| 1999 | 2241.44 | 0.42 | 2240.62 | 2242.27 | ASR | Male |
| 2000 | 2173.54 | 0.42 | 2172.72 | 2174.35 | ASR | Male |
| 2001 | 2099.71 | 0.41 | 2098.91 | 2100.51 | ASR | Male |
| 2002 | 2024.35 | 0.4 | 2023.56 | 2025.13 | ASR | Male |
| 2003 | 1939.43 | 0.39 | 1938.67 | 1940.20 | ASR | Male |
| 2004 | 1858.90 | 0.38 | 1858.15 | 1859.65 | ASR | Male |
| 2005 | 1788.18 | 0.37 | 1787.45 | 1788.92 | ASR | Male |
| 2006 | 1717.89 | 0.36 | 1717.17 | 1718.60 | ASR | Male |
| 2007 | 1642.86 | 0.35 | 1642.17 | 1643.56 | ASR | Male |
| 2008 | 1560.63 | 0.34 | 1559.96 | 1561.30 | ASR | Male |
| 2009 | 1480.48 | 0.33 | 1479.83 | 1481.13 | ASR | Male |
| 2010 | 1400.83 | 0.32 | 1400.20 | 1401.46 | ASR | Male |
| 2011 | 1319.74 | 0.31 | 1319.13 | 1320.35 | ASR | Male |
| 2012 | 1250.38 | 0.30 | 1249.79 | 1250.98 | ASR | Male |
| 2013 | 1183.30 | 0.29 | 1182.72 | 1183.88 | ASR | Male |
| 2014 | 1112.61 | 0.28 | 1112.05 | 1113.16 | ASR | Male |
| 2015 | 1014.39 | 0.27 | 1013.85 | 1014.92 | ASR | Male |
| 2016 | 929.61 | 0.26 | 929.10 | 930.11 | ASR | Male |
| 2017 | 847.46 | 0.25 | 846.98 | 847.94 | ASR | Male |
| 2018 | 769.97 | 0.23 | 769.51 | 770.43 | ASR | Male |
| 2019 | 704.78 | 0.22 | 704.34 | 705.22 | ASR | Male |
| 2020 | 651.40 | 0.22 | 650.98 | 651.83 | ASR | Male |
| 2021 | 613.34 | 0.21 | 612.92 | 613.75 | ASR | Male |
| 2022 | 555.01 | 18.88 | 518.01 | 592.00 | ASR | Male |
| 2023 | 507.43 | 20.99 | 466.28 | 548.58 | ASR | Male |
| 2024 | 463.82 | 23.42 | 417.92 | 509.71 | ASR | Male |
| 2025 | 423.87 | 25.91 | 373.09 | 474.65 | ASR | Male |
| 2026 | 387.28 | 28.31 | 331.78 | 442.77 | ASR | Male |
| 2027 | 353.74 | 30.53 | 293.90 | 413.58 | ASR | Male |
| 2028 | 323.04 | 32.50 | 259.34 | 386.74 | ASR | Male |
| 2029 | 294.96 | 34.19 | 227.94 | 361.97 | ASR | Male |
| 2030 | 269.28 | 35.60 | 199.51 | 339.04 | ASR | Male |
| 2031 | 245.79 | 36.71 | 173.83 | 317.75 | ASR | Male |
| 2032 | 224.32 | 37.56 | 150.70 | 297.94 | ASR | Male |
| 2033 | 204.71 | 38.15 | 129.93 | 279.49 | ASR | Male |
| 2034 | 186.81 | 38.50 | 111.35 | 262.27 | ASR | Male |
| 2035 | 170.48 | 38.63 | 94.76 | 246.20 | ASR | Male |
| 2036 | 155.58 | 38.57 | 79.99 | 231.17 | ASR | Male |
| 1990 | 2968.83 | 0.50 | 2967.85 | 2969.80 | ASR | Female |
| 1991 | 2889.60 | 0.49 | 2888.64 | 2890.57 | ASR | Female |
| 1992 | 2825.12 | 0.49 | 2824.17 | 2826.07 | ASR | Female |
| 1993 | 2751.01 | 0.48 | 2750.07 | 2751.95 | ASR | Female |
| 1994 | 2676.14 | 0.47 | 2675.21 | 2677.07 | ASR | Female |
| 1995 | 2623.06 | 0.47 | 2622.14 | 2623.99 | ASR | Female |
| 1996 | 2542.31 | 0.46 | 2541.40 | 2543.22 | ASR | Female |
| 1997 | 2462.24 | 0.46 | 2461.35 | 2463.14 | ASR | Female |
| 1998 | 2374.33 | 0.45 | 2373.45 | 2375.21 | ASR | Female |
| 1999 | 2295.05 | 0.44 | 2294.19 | 2295.92 | ASR | Female |
| 2000 | 2223.63 | 0.44 | 2222.77 | 2224.48 | ASR | Female |
| 2001 | 2141.16 | 0.43 | 2140.33 | 2142.00 | ASR | Female |
| 2002 | 2065.10 | 0.42 | 2064.28 | 2065.92 | ASR | Female |
| 2003 | 1980.40 | 0.41 | 1979.60 | 1981.20 | ASR | Female |
| 2004 | 1897.03 | 0.40 | 1896.25 | 1897.81 | ASR | Female |
| 2005 | 1824.08 | 0.39 | 1823.32 | 1824.85 | ASR | Female |
| 2006 | 1753.26 | 0.38 | 1752.51 | 1754.00 | ASR | Female |
| 2007 | 1675.91 | 0.37 | 1675.18 | 1676.64 | ASR | Female |
| 2008 | 1595.13 | 0.36 | 1594.42 | 1595.83 | ASR | Female |
| 2009 | 1514.60 | 0.35 | 1513.92 | 1515.28 | ASR | Female |
| 2010 | 1431.64 | 0.34 | 1430.98 | 1432.30 | ASR | Female |
| 2011 | 1349.55 | 0.33 | 1348.91 | 1350.19 | ASR | Female |
| 2012 | 1272.70 | 0.32 | 1272.08 | 1273.32 | ASR | Female |
| 2013 | 1204.83 | 0.31 | 1204.23 | 1205.43 | ASR | Female |
| 2014 | 1131.51 | 0.30 | 1130.93 | 1132.09 | ASR | Female |
| 2015 | 1028.20 | 0.28 | 1027.64 | 1028.75 | ASR | Female |
| 2016 | 941.18 | 0.27 | 940.65 | 941.70 | ASR | Female |
| 2017 | 855.56 | 0.26 | 855.06 | 856.06 | ASR | Female |
| 2018 | 775.42 | 0.24 | 774.94 | 775.90 | ASR | Female |
| 2019 | 708.22 | 0.23 | 707.76 | 708.67 | ASR | Female |
| 2020 | 620.01 | 0.22 | 619.59 | 620.44 | ASR | Female |
| 2021 | 581.45 | 0.21 | 581.03 | 581.87 | ASR | Female |
| 2022 | 522.82 | 18.20 | 487.14 | 558.50 | ASR | Female |
| 2023 | 471.63 | 20.22 | 431.99 | 511.27 | ASR | Female |
| 2024 | 425.26 | 22.51 | 381.14 | 469.39 | ASR | Female |
| 2025 | 383.32 | 24.80 | 334.71 | 431.92 | ASR | Female |
| 2026 | 345.41 | 26.92 | 292.65 | 398.18 | ASR | Female |
| 2027 | 311.13 | 28.79 | 254.71 | 367.55 | ASR | Female |
| 2028 | 280.14 | 30.35 | 220.66 | 339.62 | ASR | Female |
| 2029 | 252.15 | 31.58 | 190.25 | 314.06 | ASR | Female |
| 2030 | 226.90 | 32.51 | 163.18 | 290.61 | ASR | Female |
| 2031 | 204.14 | 33.13 | 139.21 | 269.07 | ASR | Female |
| 2032 | 183.63 | 33.48 | 118.02 | 249.24 | ASR | Female |
| 2033 | 165.16 | 33.57 | 99.35 | 230.96 | ASR | Female |
| 2034 | 148.53 | 33.45 | 82.97 | 214.08 | ASR | Female |
| 2035 | 133.56 | 33.12 | 68.64 | 198.49 | ASR | Female |
| 2036 | 120.10 | 32.63 | 56.14 | 184.06 | ASR | Female |
| The BAPC model was applied using observed data from 1990-2021 to project estimates from 2022-2036. UI = uncertainty interval; ASR = age-standardized rate per 100,000. | | | | | | |
